# Implementation of Cloud based Next Generation Sequencing data analysis in a clinical laboratory

**DOI:** 10.1186/1756-0500-7-314

**Published:** 2014-05-23

**Authors:** Getiria Onsongo, Jesse Erdmann, Michael D Spears, John Chilton, Kenneth B Beckman, Adam Hauge, Sophia Yohe, Matthew Schomaker, Matthew Bower, Kevin A T Silverstein, Bharat Thyagarajan

**Affiliations:** 1 Research Informatics Support Systems, Minnesota Supercomputing Institute, University of Minnesota, Room 599 Walter Library 117 Pleasant St SE, Minneapolis, MN 55455, USA; 2 Department of Laboratory Medicine and Pathology, University of Minnesota, Minneapolis, USA; 3 Applications Development, Minnesota Supercomputing Institute, University of Minnesota, Minneapolis, USA; 4 University of Minnesota Genomics Center, University of Minnesota, Minneapolis, USA; 5 Molecular Diagnostics Laboratory, University of Minnesota Medical Center Fairview, Minneapolis, USA

**Keywords:** Next generation sequencing, Cloud computing, Variant detection, Molecular diagnostics

## Abstract

**Background:**

The introduction of next generation sequencing (NGS) has revolutionized molecular diagnostics, though several challenges remain limiting the widespread adoption of NGS testing into clinical practice. One such difficulty includes the development of a robust bioinformatics pipeline that can handle the volume of data generated by high-throughput sequencing in a cost-effective manner. Analysis of sequencing data typically requires a substantial level of computing power that is often cost-prohibitive to most clinical diagnostics laboratories.

**Findings:**

To address this challenge, our institution has developed a Galaxy-based data analysis pipeline which relies on a web-based, cloud-computing infrastructure to process NGS data and identify genetic variants. It provides additional flexibility, needed to control storage costs, resulting in a pipeline that is cost-effective on a per-sample basis. It does not require the usage of EBS disk to run a sample.

**Conclusions:**

We demonstrate the validation and feasibility of implementing this bioinformatics pipeline in a molecular diagnostics laboratory. Four samples were analyzed in duplicate pairs and showed 100% concordance in mutations identified. This pipeline is currently being used in the clinic and all identified pathogenic variants confirmed using Sanger sequencing further validating the software.

## Findings

### Background

Next generation sequencing (NGS) technology has recently been introduced into clinical diagnostics, having already profoundly changed the nature and scope of genomic research in prior years. The large volume of data generated at relatively low cost makes NGS an ideal platform for comprehensive mutation analysis of both constitutional disorders (i.e. germline alterations) and cancer diagnostics (i.e. somatic alterations). Sequencing single genes in the work-up of suspected inherited conditions using traditional Sanger sequencing methods have historically been time consuming and costly. However, with the advent of NGS, clinical laboratories can offer cost-effective comprehensive gene panels for several complex inherited disorders [1,2]. Likewise, the field of cancer biology has rapidly evolved with the identification of a finite number of oncogenes and tumor-suppressor genes commonly implicated in a variety of tumors [3]. As we enter the era of personalized medicine, NGS offers the opportunity to conduct comprehensive genetic analysis of cancers as part of routine companion diagnostic testing to guide appropriate therapy [4,5]. More recently, NGS testing has even entered the realm of clinical infectious disease testing, primarily with sequencing of the bacterial 16S rRNA gene region [6]. Thus, NGS testing has broad clinical applicability and is ideally suited to address diagnostic issues across multiple medical specialties.

One major challenge to implementation of NGS based technology in clinical laboratories is the development of scalable and robust bioinformatics infrastructure (human and computational resources) to effectively handle the volume of data produced. While many tools are available for various aspects of refining NGS sequence data to actionable information, they usually require expert users to put them together into an effective workflow. Processing frameworks such as Galaxy [7-9] have made many of these tools much more accessible to less technically savvy users, but still require a great deal of bioinformatics expertise to set up a fully-operational data analysis pipeline. Though NGS has been incorporated into clinical testing at several reference laboratories, purchasing the computing power necessary to perform NGS testing is cost-prohibitive for the vast majority of clinical diagnostics laboratories. The Galaxy team addressed this limitation by providing CloudMan [10]. CloudMan is available as an Amazon Web Services (AWS) public Amazon Machine Image (AMI). CloudMan is set up such that anyone with an AWS account can log into Amazon’s infrastructure and start any size compute cluster they need for their data at a relatively low per hour rate. Building onto the initial CloudMan release, several other tools such as Cloud BioLinux [11], BioCloudCentral [12] and CloVR [13] have been created to improve various operational aspects and to address specific use cases not originally handled by CloudMan. However, none of these solutions met the needs of our clinical application since the storage needs for these solutions did not give us the flexibility we needed to control storage costs and obtain a pipeline that was cost-effective on a per-sample basis. CloudMan for instance requires the usage of EBS disk while running analyses, which can be substantial. Our solution does not require the usage of EBS disk to run a sample - it only uses the transient storage associated with the compute instances that the pipelines are running. In addition to saving money - this makes it easier to predict costs and simplifies the startup and shutdown of these instances.

Our software makes it possible to control storage costs providing a solution that is cost-effective for clinical applications. In addition, another advantage of our software is that it was implemented using libcloud and hence is amenable to any other cloud infrastructure libcloud can target such as VMware vCloud (http://vcloud.vmware.com/) or OpenStack (http://www.openstack.org/).

As required by the Clinical Laboratory Improvement Amendment of 1988 (CLIA) [14], transitioning these technologies from research to clinical applications requires development of detailed standard operating procedures and validation of these tools to demonstrate precision and reproducibility in identifying the pathogenic genetic variants [15]. We describe our approach to clinical validation and implementation of a bioinformatics processing pipeline in a molecular diagnostics laboratory using cloud computing infrastructure to help manage per-sample analytical costs.

### Implementation

One of the key considerations of this bioinformatics pipeline beyond the obvious concerns of security and accuracy was its ease of use for physicians, rather than IT professionals. Galaxy is a tool dedicated to making command line tools more accessible via a common web interface. Any command line tool can be readily wrapped and displayed in Galaxy. Similarly, these tools can be linked together in workflows that will reproduce an analysis on other inputs.

The Cloud Variant Calling system allows a user to start a preconfigured Galaxy server as a virtual machine on AWS. The preconfigured Galaxy server is created by including a subset of tools from the main Galaxy server together with a few tools developed at the University of Minnesota. These additional tools, not in the main Galaxy distribution, are under “MSI” or “Masonic Cancer Center” on the tools pane. The user specifies sequence data to be uploaded to the machine as part of the configuration of the virtual machine. Once complete, the user is able to run prebuilt workflows to process the data and produce a set of variations from the hg19 reference genome present in the selected genes in the sample.

To use the software package, the user executes a secure shell (SSH) script with references to a sample sheet describing the dataset and the raw, unprocessed FASTQ files produced by the sequencer. The shell script launches a Python process that uses Python virtualenv to request several Python libraries, the most noteworthy of these libraries being libcloud and the Minnesota Supercomputing Institute’s (MSI) Galaxy Virtual Machine (VM) Launcher. Galaxy VM Launcher uses libcloud and the AWS credentials provided to create a new VM from the selected, preconfigured Galaxy AMI. Galaxy VM Launcher connects to the VM via SSH for further runtime configuration and to upload the sample files.

Since the secure copy (SCP) connection between the hosts broke at a surprisingly high frequency during initial testing, we implemented a system of dividing the file into small pieces and retrying whenever there is a failure. The size of file chunks is configurable, but defaults to 10 MB. Additionally, file chunks may be compressed, but depending on connection speed and disk speed, transfer without compression may be faster than compressing before transferring.

The Cloud Variant Calling AMI contains a static version of Galaxy with open source tools linked in pre-configured workflows. Each VM generated from the AMI will be identical with the exception of the data uploaded for analysis. The tools and workflows included in the published version require an instance type of ‘m2.xlarge’ (http://aws.amazon.com/ec2/instance-types/) due to relatively high memory requirements for the GATK Unified Genotyper. By running one VM per sample, several samples can be analyzed in parallel without impacting each other in any way. The only limit to the number of parallel analyses is the number of VMs allowed on the AWS account. By default, this is 20, but this can be expanded upon request. All communication between systems is done via SSH. Every AWS account has an SSH certificate that is used by the client to connect to AWS and the VM. For backups and e-mails, the server side user must have an SSH certificate that allows access to the server machine.

## Results

### From the user perspective

**Figure 1:**
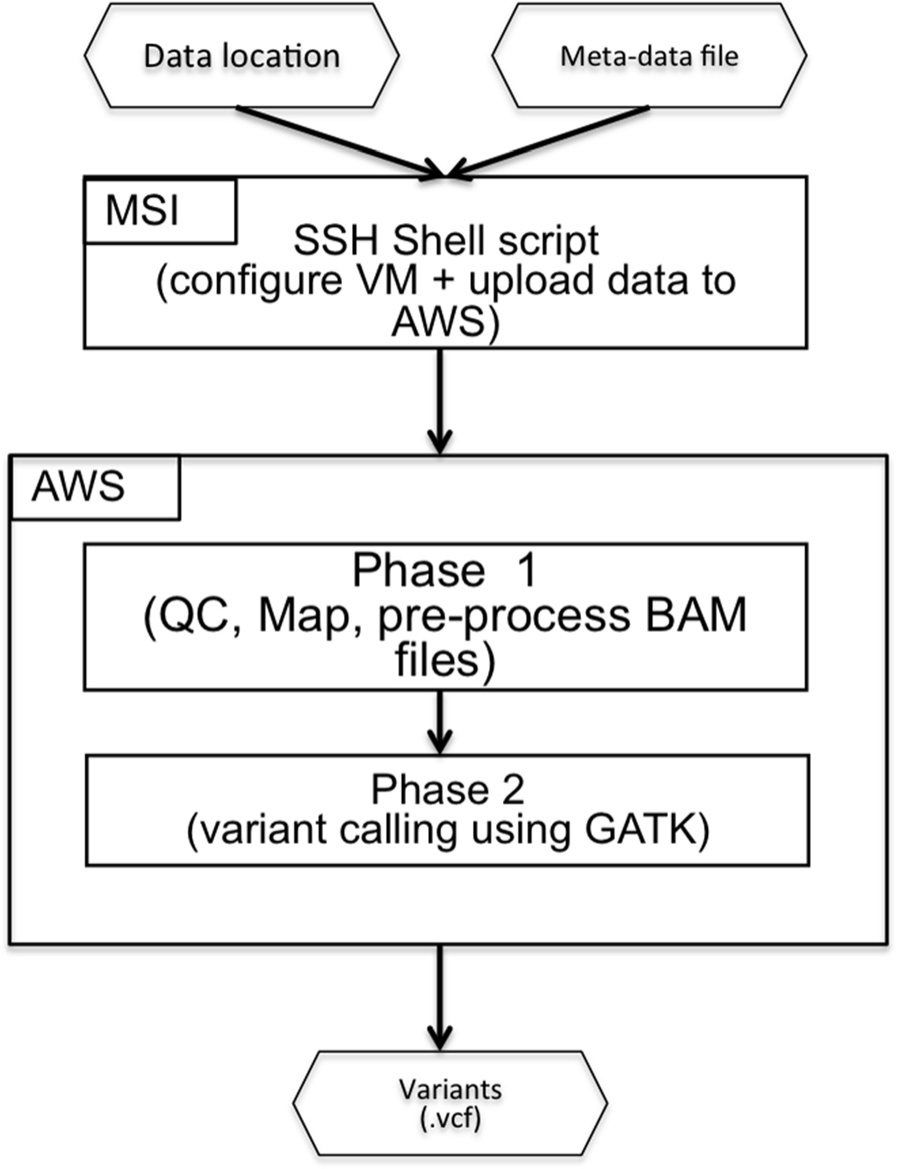
**Flowchart for analysis pipeline.** A metadata file describing the sequence data being uploaded for analysis together with the location of the files are passed as input to a shell script. The shell script configures the VM on Amazons AWS and uploads data to the VM. A Galaxy workflow is used for Phase 1 of the analysis. QC results are examined to verify data meets quality thresholds. A second Galaxy workflow is used for Phase 2 of the analyses producing a VCF file containing variants.

The Cloud Variant Calling software package, from an end-user perspective, consisted of a command line tool that launches a customized virtual machine with Galaxy running and their data loaded for analysis. The user(s) executed workflows to perform the analysis and downloaded the resulting outputs for manual review of the Variant Call Formatted (VCF) variants, along with the supporting alignments, which were inspected using the Integrative genome viewer (IGV) [16,17]. In our customized version, the workflow also created backup copies of the relevant inputs and outputs. A flowchart summarizing the main steps of the pipeline is presented in Figure 1.

Launching a VM required two inputs: a metadata file describing the sequence data being uploaded for analysis and the location of the files to upload (“*Meta-data*” file and “*Data location*” in Figure 1). A project directory was created to store the inputs, and if configured, download the key outputs of each workflow. Once the VM was configured and files were uploaded, the hostname was created to capture the URL of the VM. The custom installation at MSI notified users via e-mail when the VM was ready.

**Figure 2:**
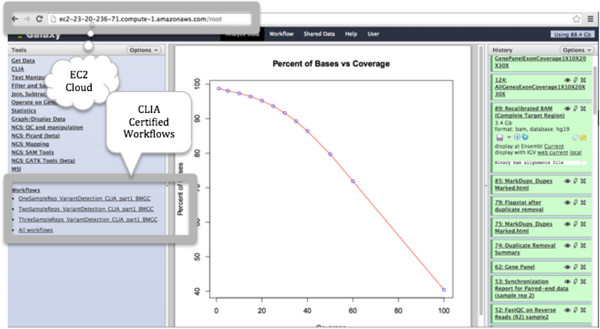
**The Galaxy analysis pipeline.** The URL gives a link to a virtual machine on the amazon cloud that runs the analysis pipeline. Galaxy interface is configured to make the CLIA certified workflows accessible as tools under the tools pane (left pane). The center pane shows results for one of the QC analyses (coverage plot outlining percent of bases with different levels of coverage). The right pane is a history of all the tools and the order in which they were executed by the pipeline together their results.

By default, files were made accessible to the user via a shared data library called “Uploaded data”. The configuration files made it possible to create a name history for a specific user and link the uploaded data to this history. Hence, the users had fewer steps to follow before launching the workflow. Likewise, workflows could explicitly be linked in the users tool menu, or accessed under the workflow menu at the top of the Galaxy window (Figure 2).

The provided workflows were constructed to work in two phases. The first phase (“*Phase 1” in *Figure 1) performed several quality control steps and aligned the data to a reduced human genome (to speed up computation time and reduce analysis cost without affecting accuracy) that matches the 568 gene panel used at the University of Minnesota along with homologous regions that match at least one of the 120 bp capture baits with a minimum of 75% identity for at least 48 bp in length. Workflows were available for samples sequenced on one, two or three lanes of an Illumina Hi-seq 2000 to account for variations in number of clinical samples being analyzed on a particular sequencing run. In a majority of the runs, we multiplexed samples across three lanes to reduce the likelihood of needing to resequence in the event of a failure in one lane of a run. The second phase (“*Phase 2” in* Figure 1) used the alignments from the first phase to generate known variants from the reference genome and reduced them to the specific panel requested by the physician. Resulting variants (“*Variants” in *Figure 1) were downloaded and reviewed by genetic counselors and pathologists to generate patient reports. For a detailed explanation and guide to using the workflows, see Additional file 1.

### Amazon Web Services (AWS) costs

Runtime costs at AWS for the current deployment averaged around $30-$40 per sample analyzed. This cost included all compute time from launch including uploading 20 GB of data per sample, running both workflows, downloading results and human response time to the completion of each of these steps. Uploading data took approximately two hours, the first workflow ten hours and the second workflow an additional two hours. Due to the memory requirements of the process, the hourly rate for a VM was $0.45. We averaged the cost of using the pipeline for actual clinical data sets over a period of three months. A total of 110 samples with fastq files ranging from 27 GB to 42 GB in size (uncompressed) were analyzed. The average total cost for the three months was $34.60.

### Clinical validation

To meet clinical validation standards, four samples were analyzed in duplicate through the entire system including the analysis pipeline in the cloud to ensure that the identical variations in specific exons were detected in the duplicate samples. The four duplicate pairs showed 100% concordance in the mutations that were identified during these analyses. Furthermore, this pipeline is currently being used in the clinic and all identified pathogenic variants were confirmed using Sanger sequencing further validating the software [18]. By freezing the entire virtual machine, from operating system through the Galaxy server to the individual tool versions, we were able to ensure consistent results across multiple analyses. Periodic reanalysis of these samples were used for continued validation that the system was working properly due to the elements at both AWS and MSI, which may change outside the control of this particular project.

## Discussion

We demonstrate the clinical validation and feasibility of implementing a cloud based bioinformatics pipeline for analysis of NGS data for use in a clinical molecular diagnostics laboratory.

The analysis workflow described in this manuscript can be completely customized for analysis of any panel of genes. The workflow described for analysis of the 568 genes can be expanded or use an entirely separate set of exons by replacing the reduced genome, exon interval file and exon with 30 bp flanks BED file in the launcher/common_files directory. To create a customized reduced genome use the “Create Reduced Genome” workflow included on the Cloud Variant Calling VM. The contents of the launcher/common_files are uploaded to every VM during initialization and the files are selected where appropriate when running the other workflows. Several options exist with regards to how to develop a customized Galaxy for deployment as an AMI. Workflows can be created on an existing local version of Galaxy then exported. The exported workflow can be included in the AMI during the AMI creation step. Alternatively, a local development Galaxy can be created using Galaxy VM Launcher on Virtual Box or Open Stack VM environments, or a development Galaxy can be deployed directly to AWS. In any of these three cases, the development VM will have to run from a local copy of the Galaxy repository to be deployed on the final AMI. Any changes made to the running system simply need to be committed to the repository before the AMI is built. During construction of the workflow it is important to consider the tools to be used. Open source tools that are already available for Ubuntu, the operating system on the AMI, are used as the starting point for the Cloud Variant Calling AMI. Deploying a tool that exists in the Ubuntu ecosystem can be as simple as adding the package name to a configuration file. However, it is possible to script the installation of other tools during AMI creation via the Galaxy VM Launcher.

When the workflow and associated tools are ready to be packaged into an AMI, the packaging script, package.sh, included with the downloaded software can be run to build a clean VM to create the AMI. Once the VM is running, the user can connect via SSH and run through the three steps presented in the AWS documentation on creating a custom AMI. The resulting AMI ID from the final registration step will be copied into the Galaxy VM Launcher configuration for use in the deployment.

Much of the cost so far has been due to human and other errors that have led to re-analysis of several samples. The most common cause so far has been discrepancies in the gene names produced from the ordering form used by the sequencing center and the exon interval files used to filter results. The discrepancies and user input validation have been resolved and eliminated from our SOP so that costs are expected to decline to the estimated compute time cost of approximately $10 per sample. In addition to these, other factors that could be addressed are increased automation and performance enhancements, both in terms of compute time and memory requirements. Automation via BioBlend [19] and the Galaxy API could reduce or eliminate any time accumulated due to human response time. Streamlining the published workflow would directly reduce the amount of time required to process a sample. Finally, the per hour rate for compute time could be lowered if reductions in the memory requirement can be made. The hourly rate is based on which EC2 instance type is selected for the VM. Once changes to any aspect of the system are ready to be deployed, the validation procedure can be applied prior to deploying the changes.

Galaxy VM Launcher is now a part of CloudBioLinux [11] and BioCloudCentral [12] and is no longer under active development. All three projects have similar goals and requirements. The best parts of each continue on as part of the remaining two projects. Future versions of the Cloud Variant Calling application will use CloudBioLinux [11] and be available for use via BioCloudCentral [12]. From a security standpoint, the data is encrypted in transit, but the temporary storage used at Amazon is not currently encrypted. While there is no protected health information (PHI) associated with the data we use and the sequence data is not a complete genome, setting up an encrypted file system on each VM’s instance specific file system would provide end-to-end encryption during the analysis process.

## Conclusions

Several efforts are ongoing involving the analysis of NGS data in the cloud [10-12,19,20]. The successful implementation of cloud based bioinformatics analysis pipeline in a clinical laboratory demonstrates feasibility of using cloud based resources in clinical settings. With end-to-end encryption and the requisite care for encryption keys and PHI in combination with the ability to control the tools used, cloud based computing can be safely and reasonably used for appropriate clinical use. The effort that is currently needed is bioinformatics to determine the most common clinical applications and provide standardized workflows via this or similar methods. Commercial companies could also leverage this method to provide low cost analysis as an add-on to sequencing data for clinicians.

## Availability and requirements

**Project Name:** Cloud Variant Calling

**Project Home Page:**https://bitbucket.org/riss/cloud-variant-calling

**Operating System:** Linux (tested on CentOS)

**Programming Language:** Python

**License:** GNU GPL

**Other requirements:** Amazon Web Services account

**Guide on using Galaxy workflow for variant detection:** Additional file 1

## Abbreviations

AWS: Amazon web services; EC2: AWS Elastic compute cloud; NGS: Next generation sequencing; SCP: Secure copy; SSH: Secure shell; PHI: Protected health information; VM: Virtual machine; MSI: Minnesota supercomputing institute; AMI: Amazon machine image; IGV: Integrative genome viewer; VCF: Variant call formatted.

## Competing interests

The authors declare that they have no competing interests.

## Authors’ contributions

GO: Workflow design and implementation, manuscript draft and editing. JE: Architecture, software implementation, testing, deployment, training, preparing initial draft of the manuscript. MDS: Analysis, user testing, preparing draft of the manuscript. JC: Software implementation of galaxy_vm_launcher and subsequent merge with other projects. KB: Director of the University of Minnesota Genomics Center, user testing and feature specification. AH: Sequencing, user testing. SY: Design of clinical validation, feature specification and user testing. MS: User testing, analysis. MB: User testing, feature specification and analysis. KATS: Bioinformatics support, workflow design and implementation and software coding for reduced genome construction, manuscript editing. BT: PI, head of Molecular Diagnostics Lab, user testing and feature specification, manuscript editing. All authors read and approved the final manuscript.

## Supplementary Material

Additional file 1 Galaxy workflow Guide for Variant detection. Click here for file
