# Supplementary material for: Implementation of Cloud based Next Generation Sequencing data analysis in a clinical laboratory
Source: BMC Res Notes. 2014 May 23;7:314. doi: 10.1186/1756-0500-7-314 (PMC4036707; doi:10.1186/1756-0500-7-314)

Galaxy workflow guide for variant detection

1 Introduction 4
1.1 Reference materials 5
1.2 Outline of tutorial 5
2 Getting Started 6
2.1 Accessing Galaxy 6
2.2 Run "pe-sync: Paired-end synchronization" 7
2.3 Review Run "pe-sync: Paired-end synchronization" output 8
2.4 Run FastQC 9
3 Mapping with BWA 11
3.1 Map with BWA for illumina 12
4 Quality Assessment / Quality Control 16
4.1 Determine insert size distribution 17
4.2 Review insert size distribution plot 18
5 GATK Phase 1: Data Pre-processing 19
5.1 Removal of ambiguously-mapped and low-quality reads 22
5.2 Sorting Reads and updating mate-pair information 24
5.3 Removal of duplicates 25
INDEL Realignment 26
5.4 Create Targets for Realignment 26
5.5 Realign reads around INDELS 29
5.6 Remove duplicates after INDEL realignment 30
Base Quality Recalibration 31
5.7 Count Covariates (before base recalibration) 31
5.8 Analyze Covariates (before base recalibration) 33
5.9 Review Covariate plots (before base quality recalibration) 34
5.10 Recalibrate base quality scores 35
5.11 Count Covariates (after base recalibration) 36
5.12 Analyze Covariates (after base recalibration) 38
5.13 Review Covariate plots (after base quality recalibration) 39
6 GATK Phase 2: Variant Discovery 42
6.1 Variant detection using Unified Genotyper 43
6.2 Review Unified Genotyper results (Raw Variants) 47
7 GATK Phase 3: Preliminary Analysis 50
Variant Recalibration 52
7.1 Select SNPs 52
7.2 Recalibrate SNPs 55
7.3 Apply recalibration 61
7.4 Review Variant Recalibration Models 62

7.5 Review Recalibrated Variants (SNPs) 63
7.6 Select INDELs 66
7.7 Recalibrate INDELs 68
7.8 Apply recalibration 72
7.9 Review Recalibrated Variants (INDELs) 73
7.10 Combine SNPs and INDELs 76
Variant Annotation 77
7.11 Annotate variants using SnpEff 77
7.12 Review Annotated Variants 79

1 Introduction

This guide was prepared using DNA sample from a European CEPH family (NA10858) obtained from the Coriell Cell Repository (Camden, NJ, USA). It outlines a typical variant detection analysis using GATK's best practices on the Galaxy framework. The sample dataset was obtained using Hybrid-capture with RNA baits. Some of the GATK tools such as VariantRecalibrator take, as part of the input, VCF files containing known common variants "*true sites*", utilized for statistical training purposes. Data from 1000 Genomes project is used for training. It is publicly available on the 1000Genomes website (http://www.1000genomes.org/data#DataAccess) .

The following input files were used:

Sequence data: fastq files

L7_R1_CAGATC_Index_7_groomed.fastq
L7_R2_CAGATC_Index_7_groomed.fastq

Exon data: bed files

tutorial_exons.bed

Recalibration data (1000G data): vcf files

dbsnp_137.hg19.vcf
Mills_and_1000G_gold_standard.indels.hg19.vcf
hapmap_3.3.hg19.vcf
1000G_omni2.5.hg19.vcf
1000G_phase1.snps.high_confidence.hg19.vcf

★ FASTQ Files

FASTQ format is a tex-based format for storing a biological sequence and its corresponding quality scores. See http://en.wikipedia.org/wiki/FASTQ_format.

★ Bed Files

BED format provides a way for defining genomic regions. We will use BED format to define target regions e.g., exons being targeted for sequence capture. The first three required fields specify: name of chromosome, start position and end position. For more information on BED format see http://genome.ucsc.edu/FAQ/FAQformat.html#format1.

★ VCF Files

Variant Call Format (VCF), not to be confused with the standard file format for storing contact information, is a specification for storing sequence variations. For more information on VCF format see http://en.wikipedia.org/wiki/Variant_Call_Format.

1.1 Reference materials

- M. A. DePristo *et al.*, A framework for variation discovery and genotyping using next-generation DNA sequencing data. *Nat. Genet.* **43**, 491(2011).
- Genome Analysis Toolkit (GATK) website: http://www.broadinstitute.org/gatk/
- Summary of best practices for variant detection: http://www.broadinstitute.org/gatk/guide/best-practices
- BWA manual: http://bio-bwa.sourceforge.net/
- SAMtools: http://samtools.sourceforge.net/
- Picard-Tools: http://picard.sourceforge.net/
- SNP effect predictor: http://snpeff.sourceforge.net/
- Galaxy screencasts: galaxycast.org

1.2 Outline of tutorial

1 Introduction
2 Getting Started
3 Mapping with BWA
4 Quality Assessment / Quality Control
5 GATK Phase 1: Data Pre-processing
INDEL Realignment
Base Quality Recalibration
6 GATK Phase 2: Variant Discovery
7 GATK Phase 3: Preliminary Analysis
Variant Recalibration
Variant Annotation

2 Getting Started

2.1 Accessing Galaxy

a) Log in with your username and password

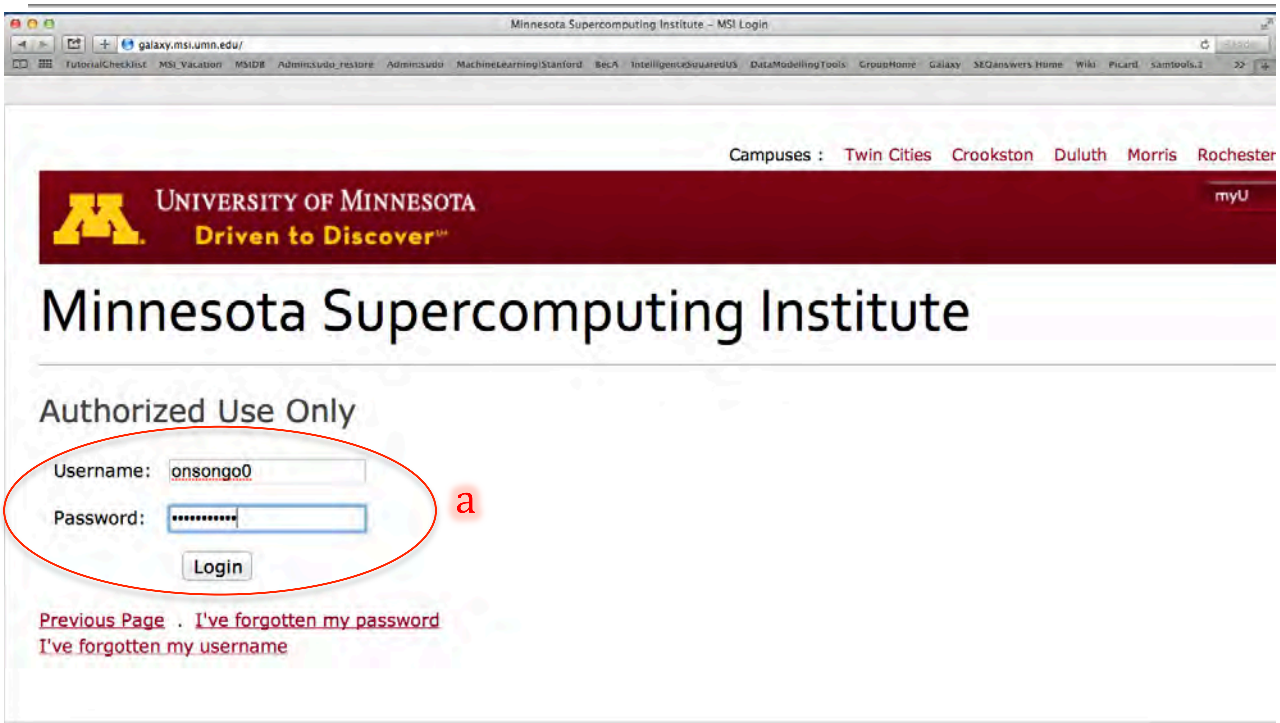

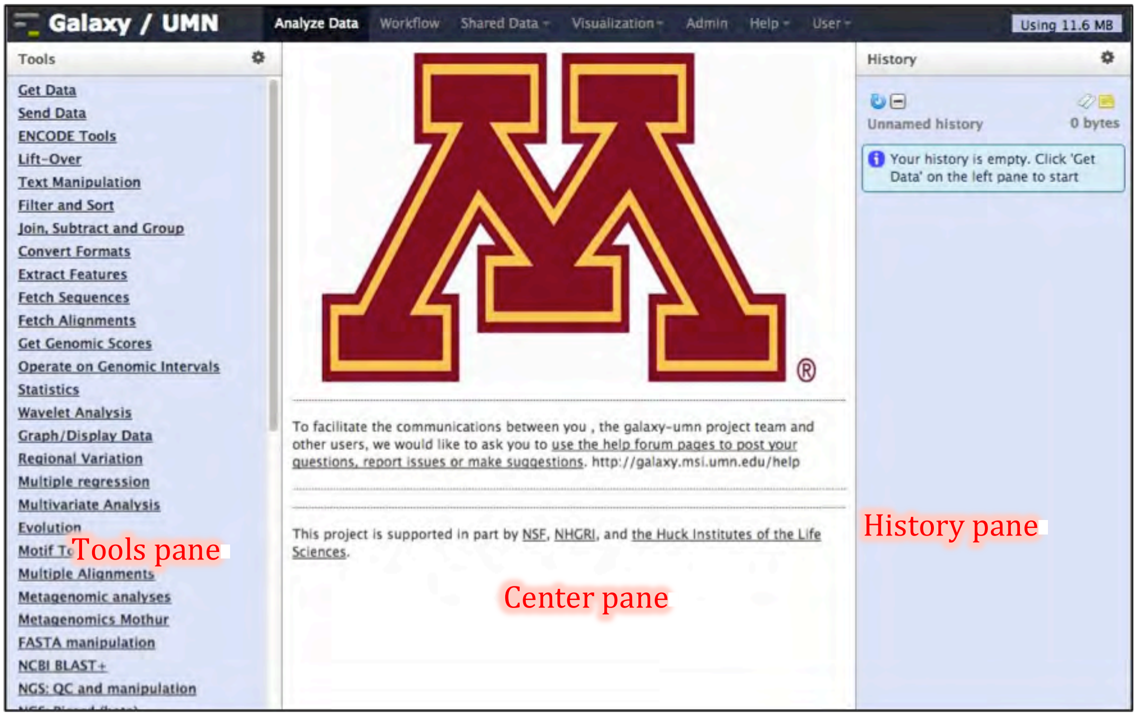

2.2 Run "pe-sync: Paired-end synchronization"

The *pe-sync* tool checks to make sure the forward and reverse reads are synchronized.

a) Load the pe-sync tool from the tool pane: "MSI -> pe-sync: Paired-end synchronization check"
b) Set input files: From the dropdown menu under
   Input 1: select "L7_R1_CAGATC_Index_7_groomed.fastq"
   Input 2: select "L7_R2_CAGATC_Index_7_groomed.fastq"
c) Click "Execute"

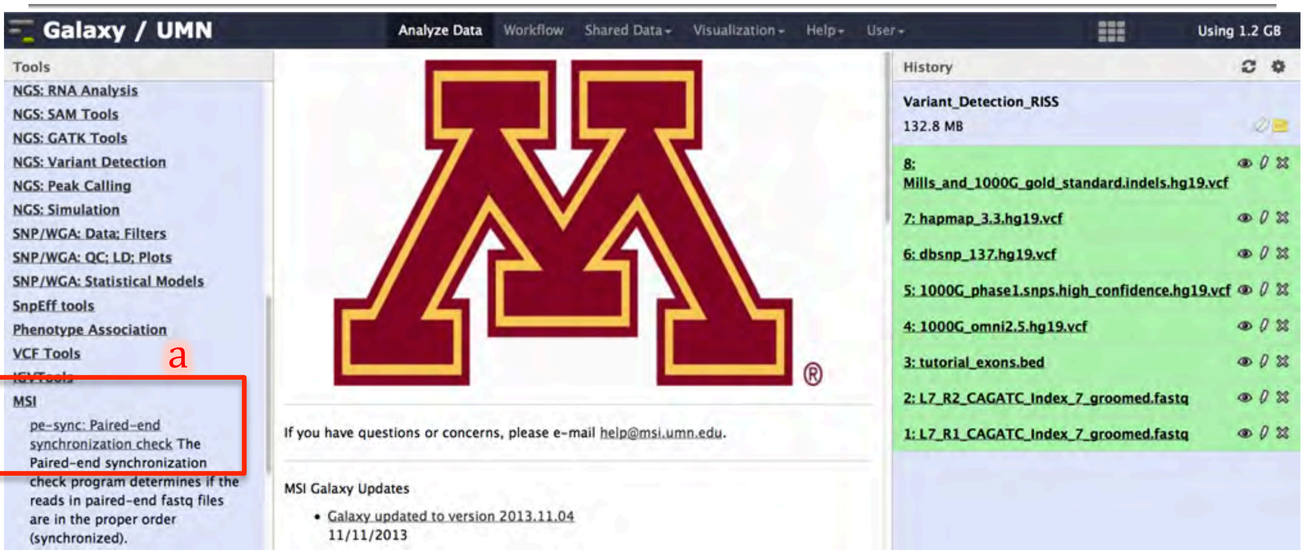

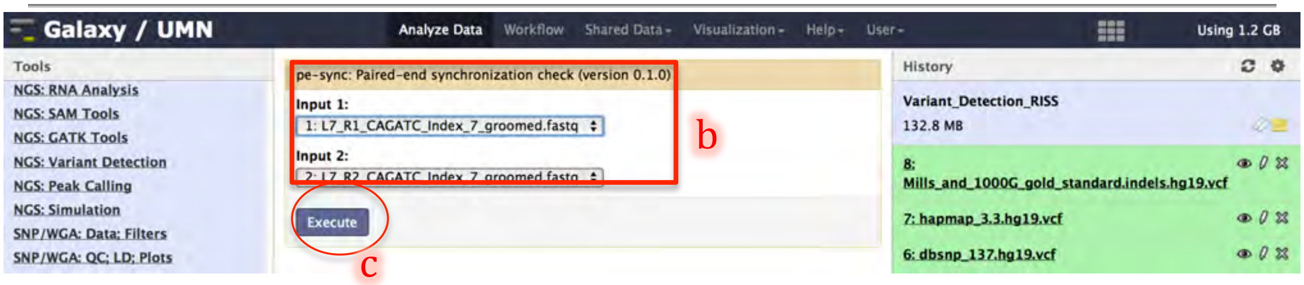

2.3 Review Run "pe-sync: Paired-end synchronization" output

a) In the history pane click on the *eye icon* next to the *pe-sync* output file to display output on the center pane
b) Verify data is synchronized

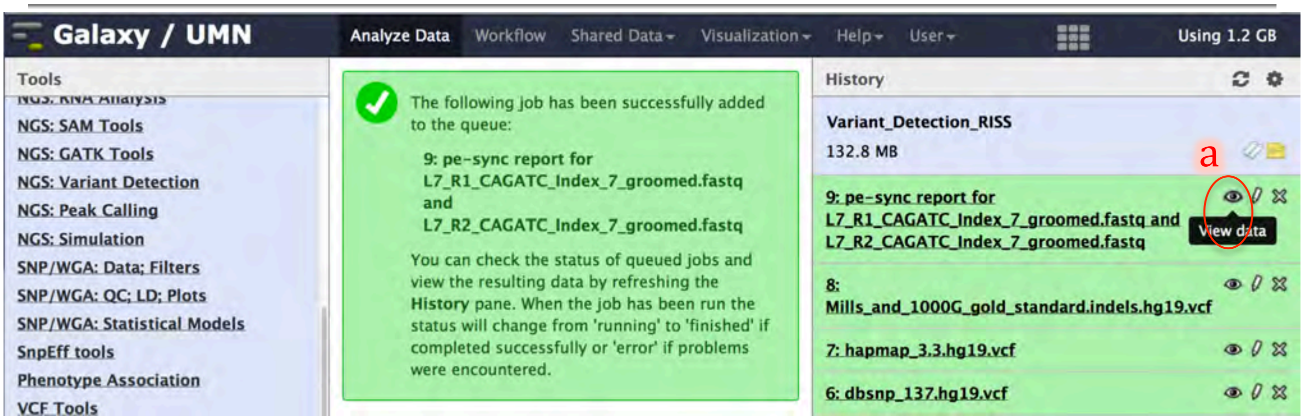

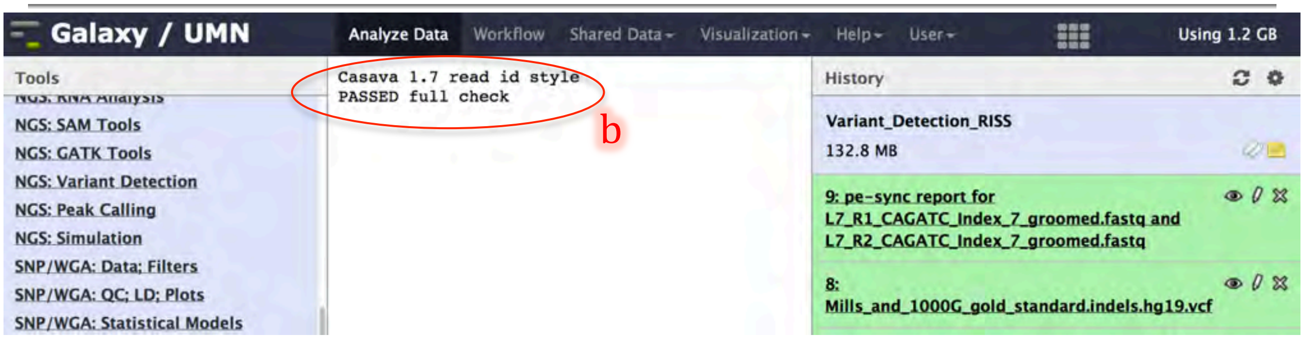

2.4 Run FastQC

a) Load the FastQC tool from the tool pane: "NGS: QC and manipulation -> FastQC: Read QC…"
b) Set the input file to select "L7_R1_CAGATC_Index_7_groomed.fastq" from the dropdown menu under "Short read data from your current history"
c) Click "Execute"

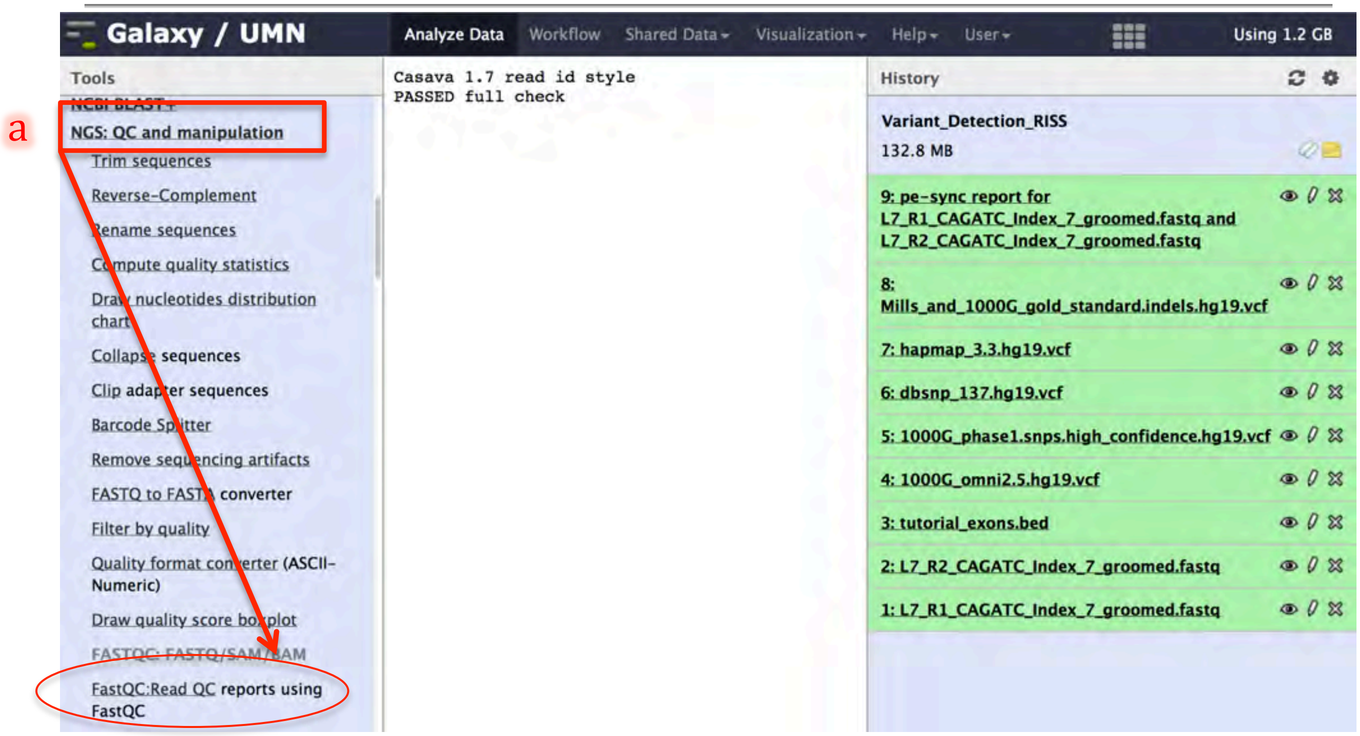

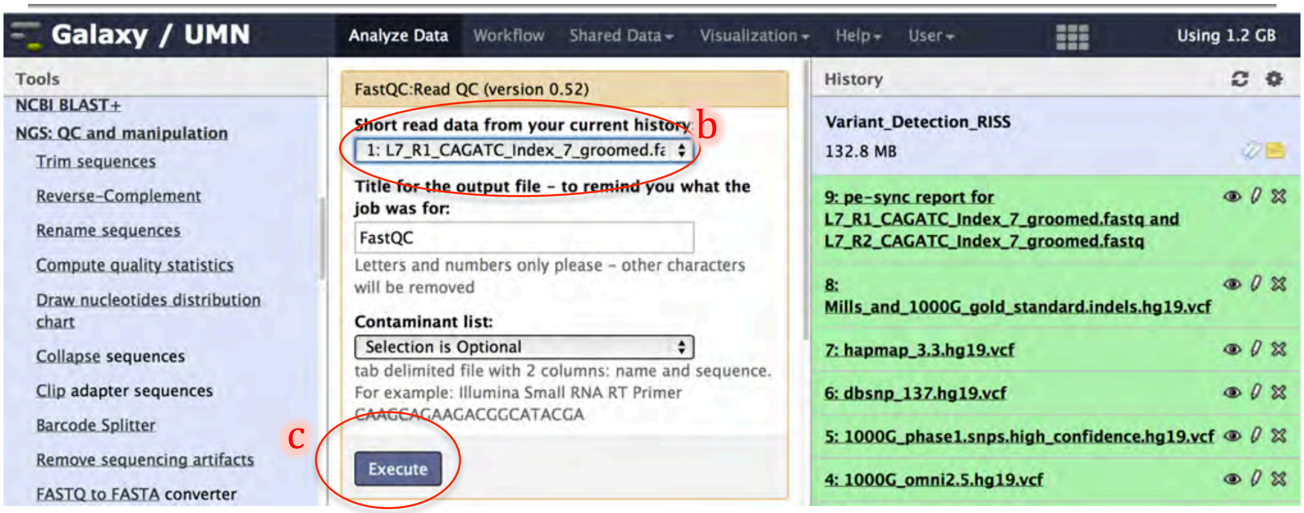

d) In the history pane click on the name of the *FastQC* output file to expand its box and show more options
e) Click the blue arrowed circle to display the *FastQC* tool in the center window to re-run the program
f) Set the input file: select "L7_R2_CAGATC_Index_7_groomed.fastq" from the dropdown menu under "Short read data from your current history"
g) Click "Execute"

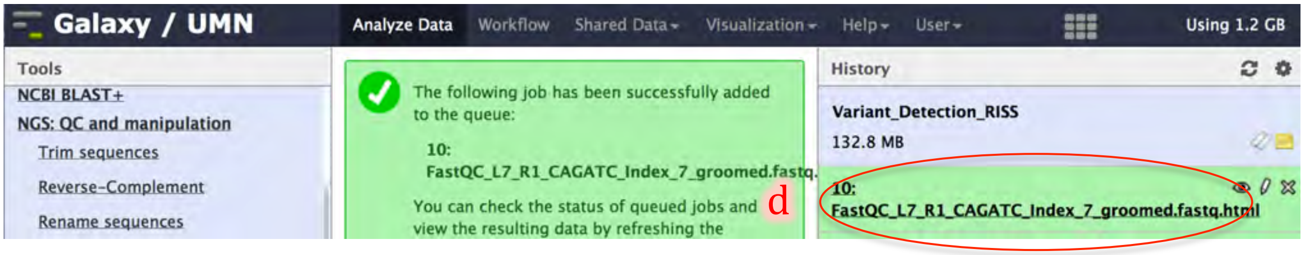

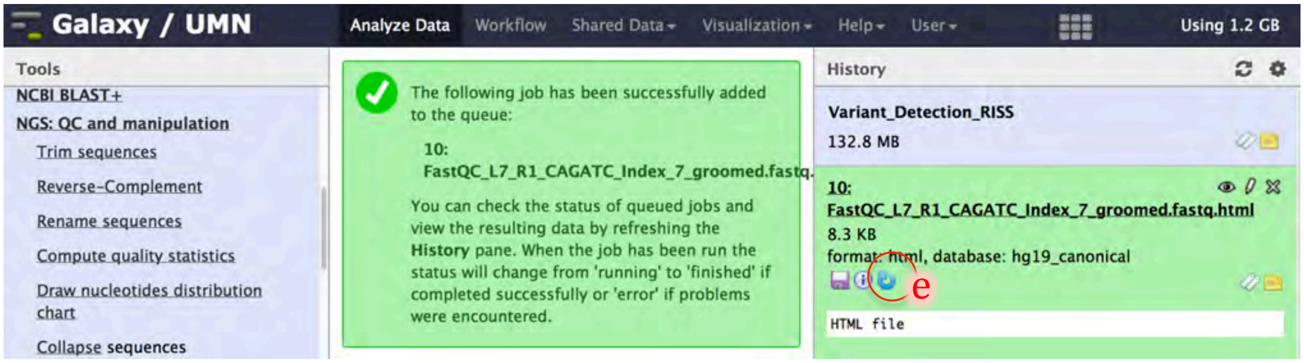

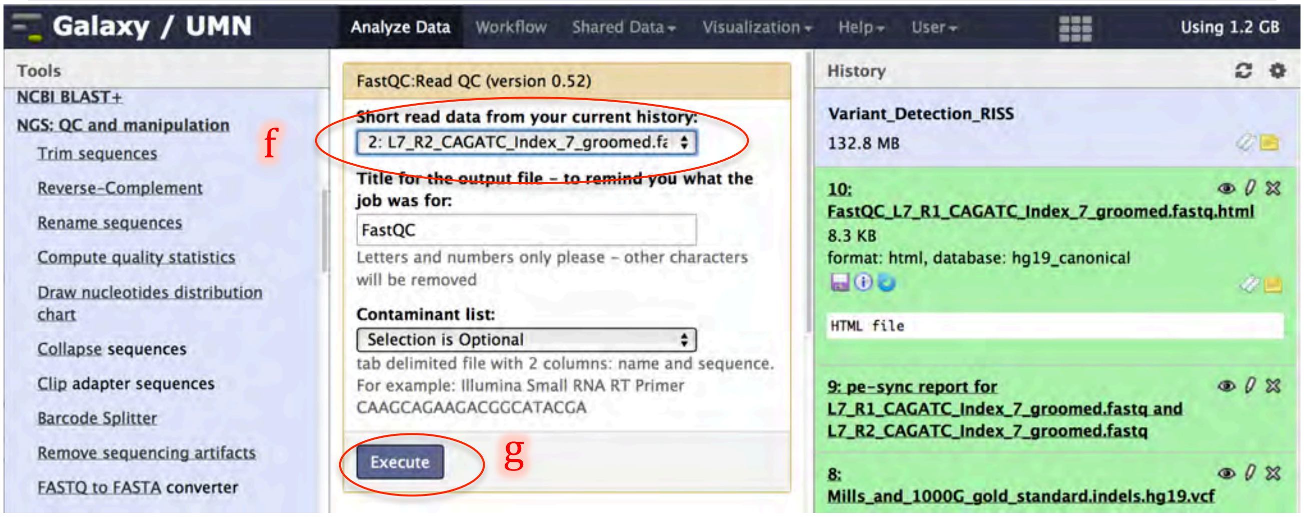

3 Mapping with BWA

For mapping illumina data to a reference genome, BWA is the recommended aligner. Among its many advantages such as accuracy and speed, it emits BAM files natively. GATK only supports the BAM format for mapped reads. For more information on BAM file format see http://samtools.sourceforge.net/SAM1.pdf.
Other aligners besides BWA can be used, provided that the BAM files satisfy the GATK's formatting requirements (see the GATK's website for more details). This section illustrates how to specify the required fields to ensure that your BAM files adhere to the GATK's formatting requirements.

★ **Reference sequence**
Reference sequences must be sorted in the order of one of the official b3x (e.g., b36, b37) or hg1x (e.g., hg18, hg19) references. A reference sequence adhering to this requirement is available in Galaxy (hg19_canonical).

★**Insert Size**
When using BWA, users will need to specify a maximum expected insert size and median insert size. The maximum expected insert size is used to determine if a read pair is mapped properly. BWA should be able to infer this information from aligned reads and only uses the specified field if there are not enough good alignments. For this tutorial data, we will use 1000 for maximum insert size and 400 for median insert size. This information can sometimes be obtained from the sequencing center but later in the tutorial we will show how to plot an insert size distribution histogram from mapped reads to determine these values. Below is a typical insert size distribution histogram.

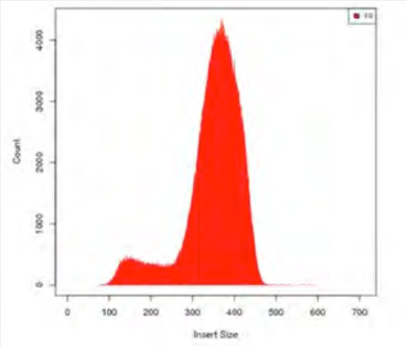

★ **Read Groups**
Many downstream processes can take in multiple alignment files, or merged alignment files, that mix reads that were run in different lanes, on different sequencers, on different dates, etc. In order to detect systematic biases that may be introduced by any of these factors, and know which reads came from where, the BAM file specification allows the assignment of "Read Groups" to any collection of reads that logically were produced together. The GATK requires that all BAM files must list the read groups with sample names in the header and every read must belong to a read group. Consequently, when running BWA the full parameters list must be used with read groups, library name, sample name and platform used to produce the reads specified. Even though not required, we encourage you to specify optional parameters such as *sequencing center (CN)* and *date run was produced (DT).* If these features are not specified, there will be no way to go back and determine if systematic biases occurred at a given center or on a given date.

3.1 Map with BWA for illumina

a) Load BWA tool from the tool pane: "NGS: Mapping -> Map with BWA for illumina"
b) Select a reference genome -> hg19_canonical
c) Is this library mate-paired? -> Paired-end
d) Forward FASTQ file, forward reads -> L7_R1_CAGATC_Index_7_groomed.fastq
e) Reverse FASTQ file, reverse reads -> L7_R2_CAGATC_Index_7_groomed.fastq
f) BWA settings to use -> Full Parameter List

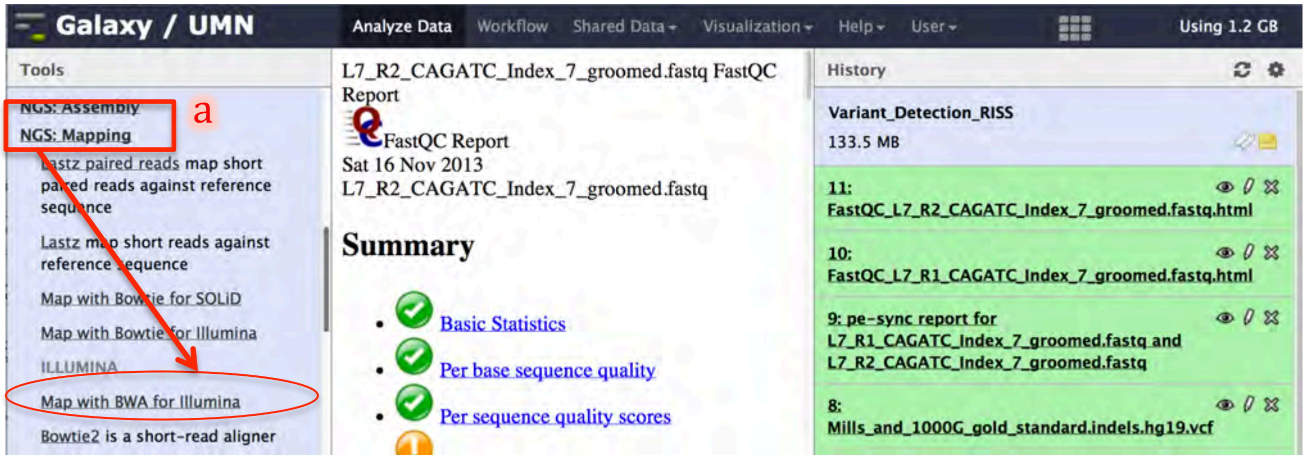

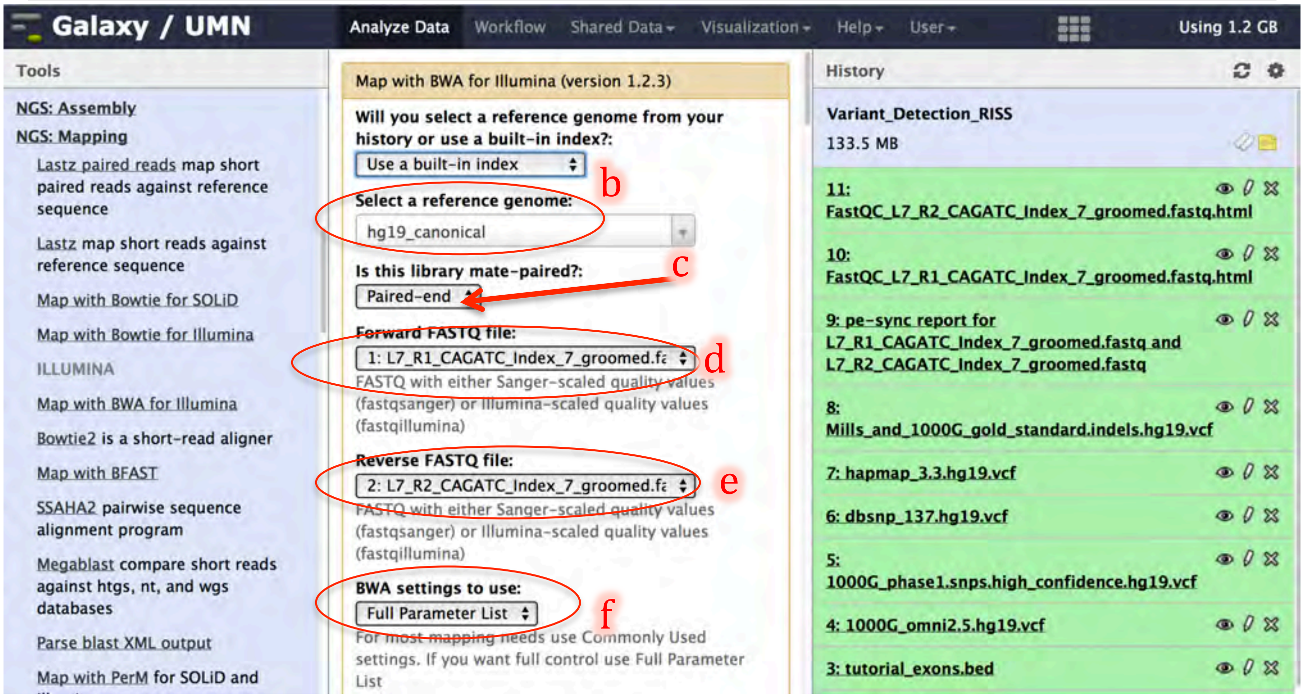

g) Maximum insert size for a read to be considered as being mapped properly (sampe –a): -> 1000
h) Specify the read group for this file -> Yes
i) Read group identifier (ID). -> NA_10858_400
j) Sequencing center that produced the read (CN): -> UMGC

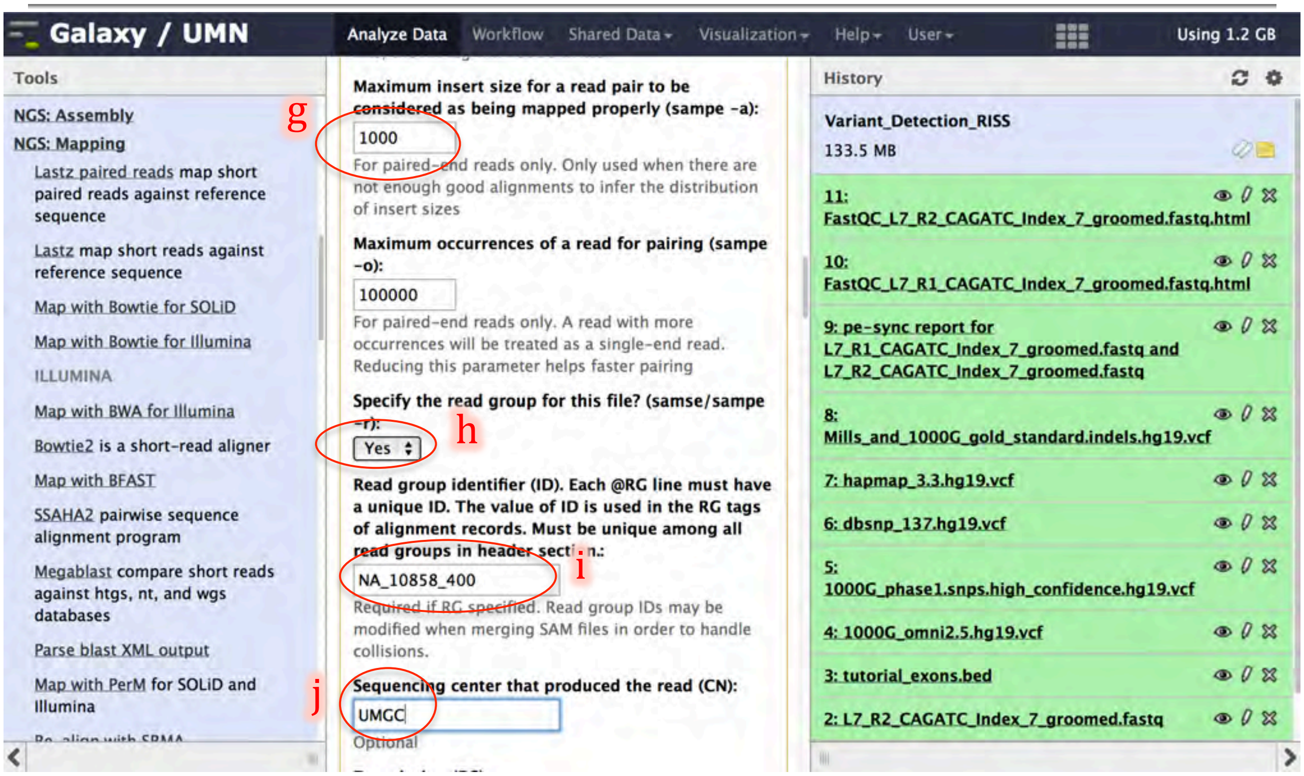

k) Description (DS): -> Coriell _HapMap_400bpInsert
l) Date that run was produced (DT): -> 2013-11-20

WARNING!! When entering the date, DO NOT use any other characters between the year, date and month e.g., 2013_11_20 WILL NOT WORK. GATK expects a DATE **data type** specified using dashes as shown (2011-05-11). Using any other characters will result in GATK producing an error message.

m) Library name (LB): -> NA_10858

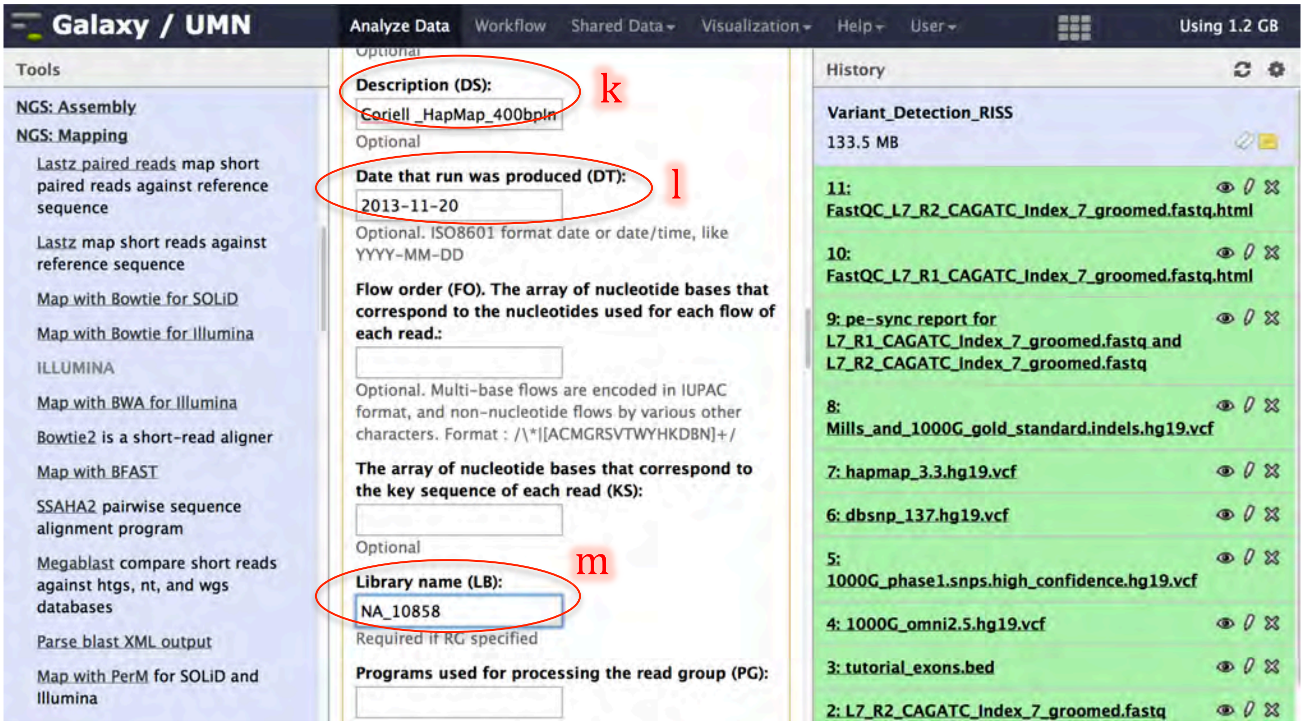

n) Predicted median insert size (PI): -> 400
o) Platform/technology used to produce the reads (PL): -> ILLUMINA
p) Platform unit (PU): -> HWUSI-EAS1737:7
q) Sample (SM): -> NA_10858
r) Click "Execute"

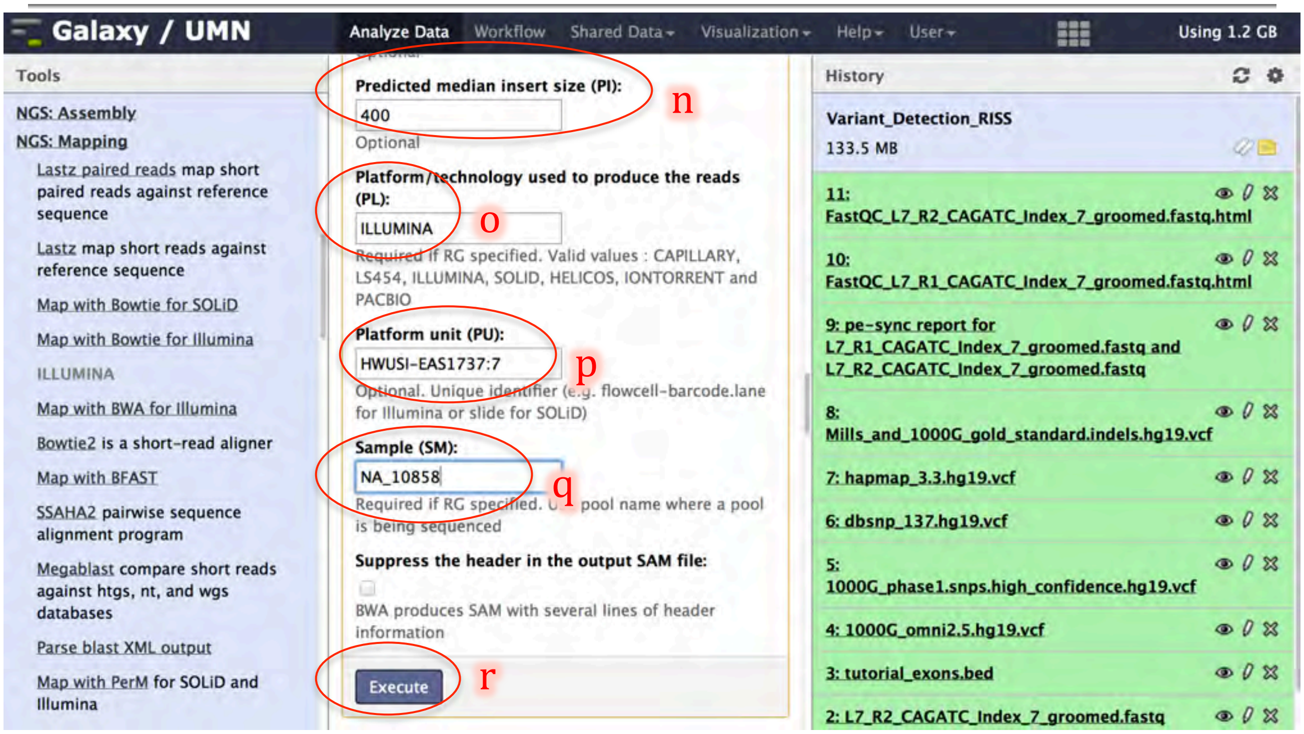

4 Quality Assessment / Quality Control

★ **Insert Size Distribution**

In section 3, we had to input, as parameters, the maximum insert size and median insert size into BWA when mapping reads to a reference genome. These values can be obtained from the sequencing center. Alternatively, one can first map reads with BWA using "Commonly Used" parameters and use the resultant output as input to a tool in Galaxy (available under Picard-Tools) to plot an insert size distribution histogram.

In addition to providing input parameter values to BWA, the insert size distribution histogram serves as an addition verification step for data integrity. Recall, BWA should be able to infer insert sizes from aligned reads and only uses supplied information if there are not enough good alignments. Generating this insert size distribution plot thus provides additional confirmation that the appropriate insert size was used. A distribution histogram differing widely from the expect insert size distribution should serve as a red flag.

4.1 Determine insert size distribution

★

a) Load insert size metrics tool from the tool pane: “NGS: Picard (beta)-> Insertion size metrics for PAIRED data”
b) SAM/BAM dataset to generate statistics for: -> “…Map with BWA for Illumina on data ……”
c) Click “Execute”

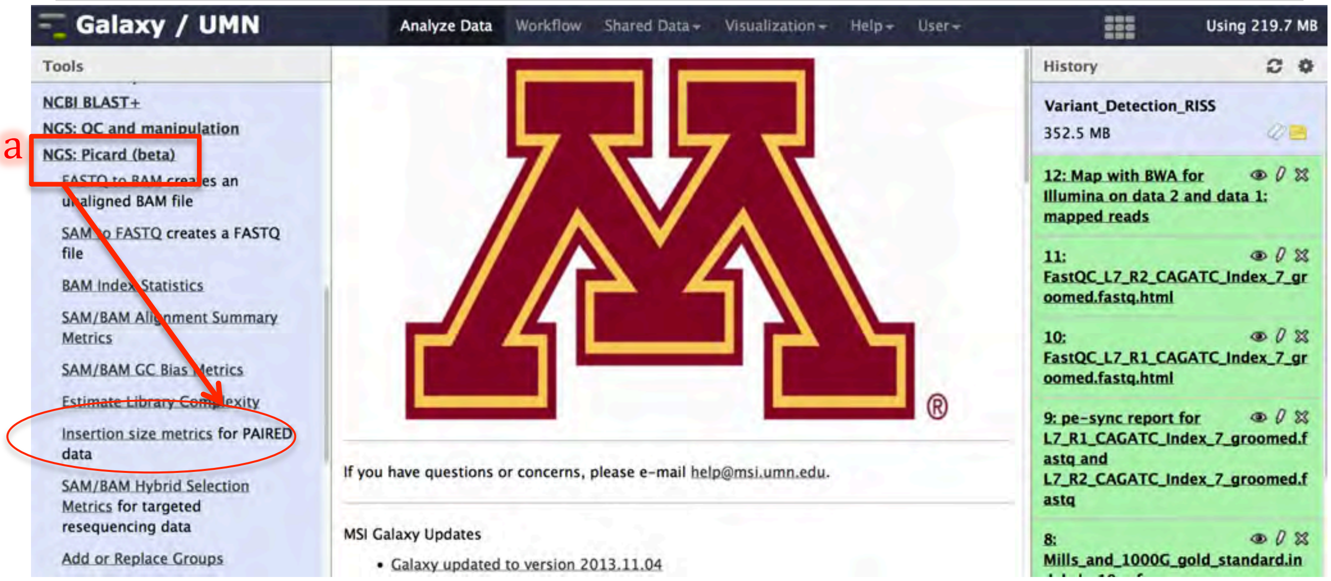

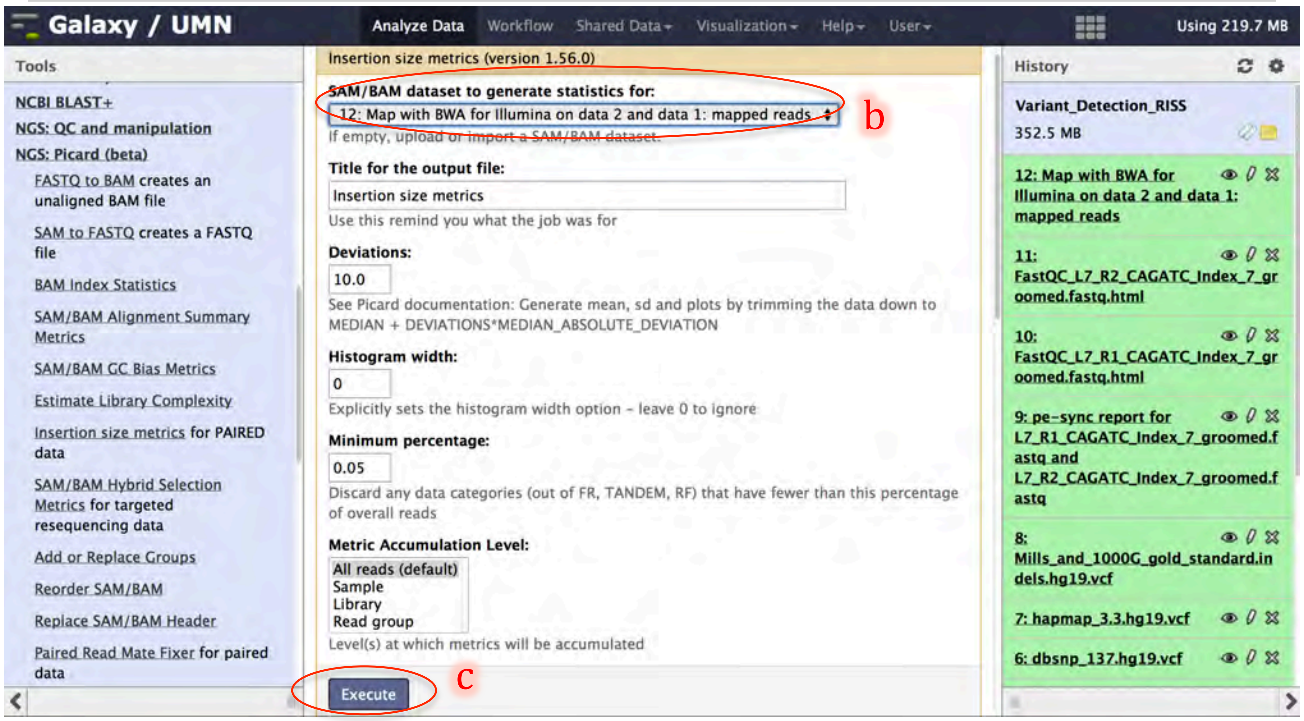

4.2 Review insert size distribution plot

a) In the history pane click on the “eye” next to the name of the insert size metric tool output file
b) If desired: right click on the image plot to download it as an image

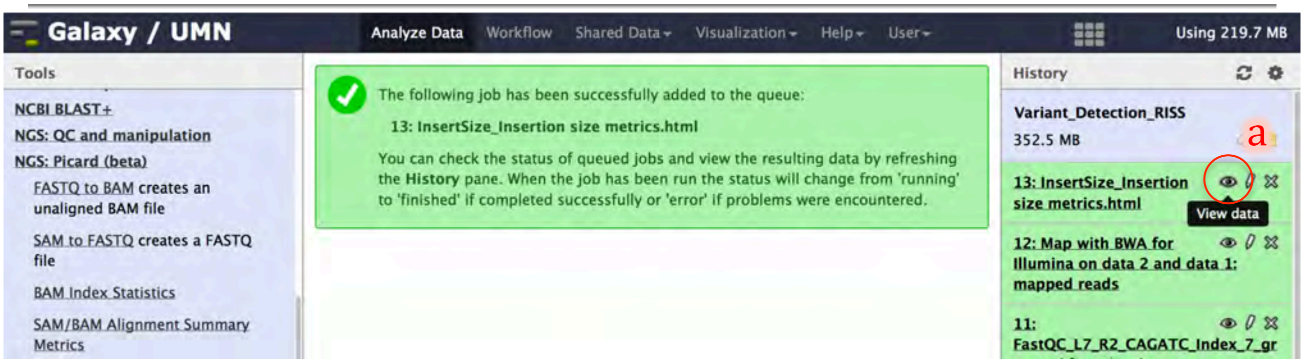

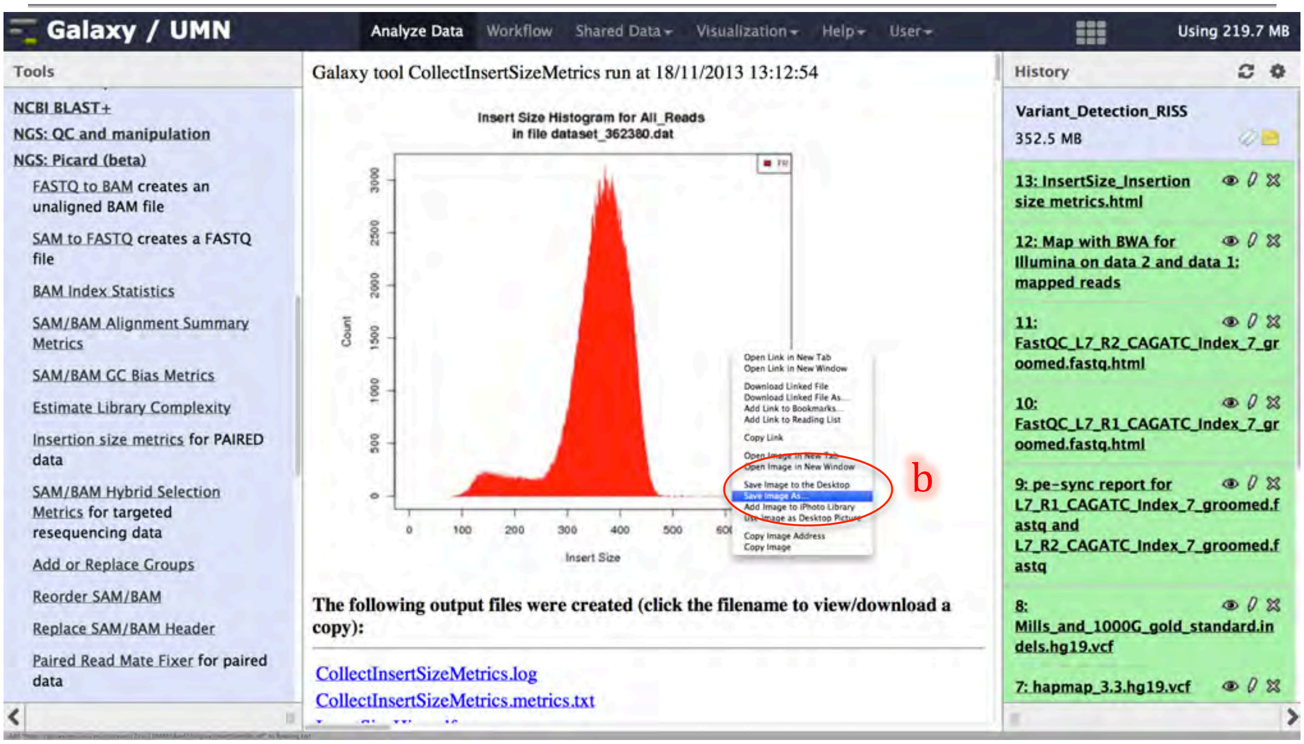

5 GATK Phase 1: Data Pre-processing

The Genome Analysis ToolKit (GATK) is an open source java programming suite for NGS data handling and variant detection that was created in support of the 1000 Genomes Project. A best-practices pipeline for variant calling based on the GATK was published by MA DePristo et al. in *Nat. Genet.* **43**, 491(2011). Updates regularly appear on the Broad's website: http://www.broadinstitute.org/gatk/. It contains three main phases: Data pre-processing, variant discovery and preliminary analysis.

★ GATK Phase 1: Data Pre-processing

In order to identify variants relative to a reference, mapping of the reads to the reference must be performed. We have already discussed mapping raw reads to the genome using BWA. However, the raw alignments generated by BWA, or by any genome mapping algorithm, are not of sufficient quality to identify real biological variants because they contain numerous systematic errors that must be filtered. In this phase, several steps are taken to clean up raw BAM files to get them ready for genotype calling.

★ GATK Phase 2: Variant Discovery

Once BAM files are cleaned of systematic artifacts, we can proceed with calling SNPs and Indels. (Structural Variants are outside the scope of this tutorial). The GATK's Unified Genotyper is the current industry standard, but is limited to diploid organisms in un-pooled samples. Accomodation of polyploid genomes and pooled samples has recently been introduced to newer versions of the GATK.

★ GATK Phase 3: Preliminary Analysis

Even when using filtered BAM files, some systematic machine artifacts pollute the raw variant calls produced by NGS pipelines. External verification tests have revealed profiles of unreliable calls (e.g., calls in regions of unusually high local depth of coverage that probably represent collapsed repeat regions, calls only supported by reads on one strand and not the other, or calls violating Hardy-Weinberg Equilibrium). Hence, in this phase, the GATK attempts to partition the raw calls into confidence classes or tranches, based on their inherent characteristics.

★ **GATK Phase 1 details**

The types of systematic biases that must be corrected in raw BAM files include:

• **Removal of ambiguously-mapped and low-quality reads**

BWA assigns a mapping quality (MAPQ) value of 0 to non-uniquely mapped reads. Read pairs that map equally well to multiple locations have *at best* a 50% chance of being mapped to the correct location, and hence are not typically suitable for variant detection. Additionally, reads that don't map in proper pairs (possibly involved in large structural rearrangements) also may reduce confidence for SNP and small indel calls. We use SAMTools (see http://samtools.sourceforge.net/) to remove these classes of problematic reads.

• **Sorting Reads and updating mate-pair information**

In addition to the input constraints by GATK, read files must be sorted in coordinate order with respect to the reference. We will use Picard-Tools to sort reads and ensure all the mate-pair information is in sync between each read and it's mate pair.

• **Removal of PCR duplicates**

Most library preps, especially those that involve sequence capture, involve several rounds of PCR. Allele frequencies and genotype calls can be skewed if certain individual sequence fragments are preferentially amplified relative to others, as shown below. Hence all paired end fragments mapping to the exact same genomic coordinates should be reduced to one copy. We use Picard-Tools instead of SAMTools to remove duplicates because it considers both reads in the pair.

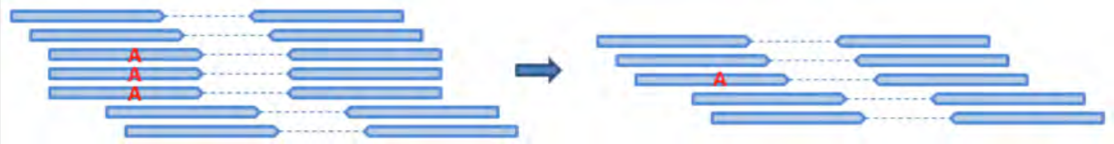

• **Indel Realignment**

Indels pose difficult challenges to mapping algorithms, especially in sequence regions with simple sequence repeats. Incorrect mapping across indels often leads to false-postitve SNPs nearby as the image below illustrates. The GATK realigner target creator systematically goes through a BAM file and identifies all sequence positions where at least one read has an indel. The GATK indel realigner will then check every read at each flagged position and determine through a liklihood ratio test whether it better matches the reference sequence or the alternate indel call, resulting in a cleaned up BAM file as below. NOTE: inclusion of known indel sites (e.g., from the 1000 Genomes Project) in addition to novel sites will improve performance.

• **Base quality recalibration**

Sequencing machines simply do not report accurate base call qualities. The phred-scale quality they report for all bases in a run is directly testable empirically, when running on a well-characterized population. Since it is estimated that 99% of all variants in the Caucasian population have been deposited in dbSNP, the vast majority of mapping differences *not in dbSNP* should simply be sequencing errors. So, if we check base calls that had a raw quality of Q20, we would expect to find about 1 novel mapping discrepancy approximately every 100 such basecalls checked – but the real numbers often reveal systematic biases.

• Base quality recalibration across covariates
The GATK allows one to explore a breakdown of empirical vs. reported quality values across many covariates. For example, an Illumina run may systematically *differentially under- or over-estimate* base quality across the length of the read as shown below. As is typical in these runs, the reported base call qualities are least accurate in the beginning and the end of each read. The GATK's base quality recalibration routines can simultaneously correct for several different covariates at once (e.g., cycle, dinucleotides, homopolymer runs), but simultaneous optimization of many covariates can be difficult in practice. (i.e., If there are any dependencies among the covariates entered, "fixing" or over-fitting one may have an adverse effect on the others.)

Phred Quality Scores

The base call quality scores (Q values) being recalibrated should not be confused with the other two Q scores used to assess mapping quality and variant call quality. The Phred quality score was developed by the program **Phred** to help in automation of DNA sequencing (see http://en.wikipedia.org/wiki/Phred_quality_score for more details). It is a logarithmic link to error probabilities ($Q = -10 \log_{10}P$) and can be used to assess the quality of any measure with error probabilities. In addition to being used to report base call qualities, Q scores is also used to measure mapping quality and variant call quality in NGS based variant detection.

• Base Quality
The base call quality Q score is a measure of how confident a sequencing machine is that the correct base call was made. For example, if 1 in 10 base calls are wrong, the probability of error is 1 in 10 (P = 0.1). Recall, Q score = $-10 \log_{10}(0.1) = -10 \log_{10}(10^{-1}) = -10(-1) = 10$. So the Q score for a base call with a 1 in 10 chance of being wrong is Q10. In a similar manner, if P=0.01 (i.e., 1 error in 100 bases) this implies Q20.

• Mapping Quality
Mapping quality Q score is a measure of how well a sequenced read maps to a reference genome. A read that uniquely maps to a reference genome will have a higher Q score value relative to a read that maps equally well to several locations in the genome. Factors that contribute to the likelihood of mapping error and hence reduce reported mapping quality include: (1) the number of alternative equal-scoring mappings in the genome and (2) the number of high-quality basecall mismatches with the reference.

• Variant Call Quality
Variant call quality Q score is measure of the likelihood of the variant call being correct. A heterozygous (A/T) call with 200 bases matching the reference allele and 199 bases matching the alternate allele is expected to have a higher Q score value relative to a variant call with 1 base matching the reference allele and 1 base matching the alternate allele. A single wrong base call will completely change the second variant call (with only 2 supporting bases, 1 for the reference allele and the 1 for the alternate allele).

Using the "Operate on Genomic intervals" (-L) analysis option
The GATK offers a –L analysis option ("Operate on Genomic intervals") that restricts analysis to a specific part of the genome. This option can be very useful in reducing computation time when users have large datasets but are only interested in a small part of the genome. For example, a user with Whole Genome Sequence data might be interested in variants in a few genes. Restricting analysis to the genes of interest will significantly reduce computation time. You generally might NOT want to restrict analysis to the region of interest. For certain tools such as the *base quality score recalibrator* and *variant call recalibrator,* addition data from other regions of the genome will improve accuracy

5.1 Removal of ambiguously-mapped and low-quality reads

a) In the history pane click on the Options wheel at the top (on the right side of the word "History") and click on "Saved Histories"
b) Switch to the History "Variant_Detection_RISS"
c) Load *Filter SAM* tool from the tool pane: "NGS: SAM Tools -> Filter SAM or BAM files on FLAG MAPQ RG LN or by region"

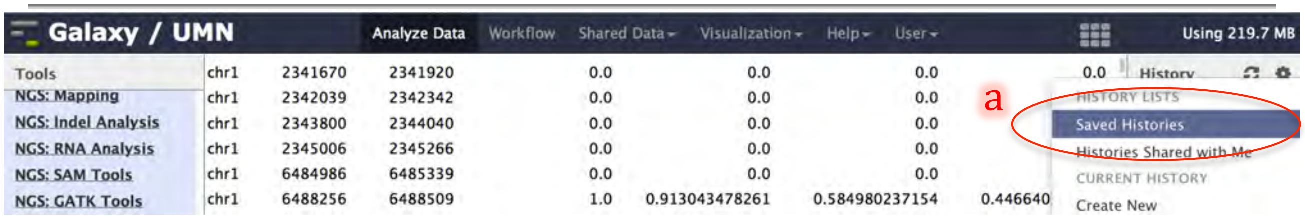

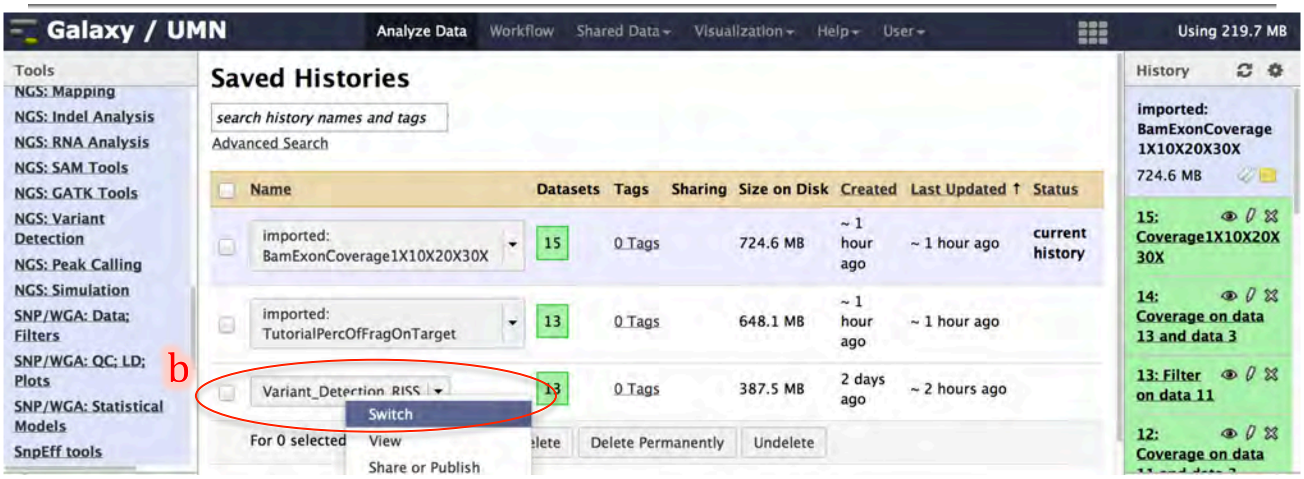

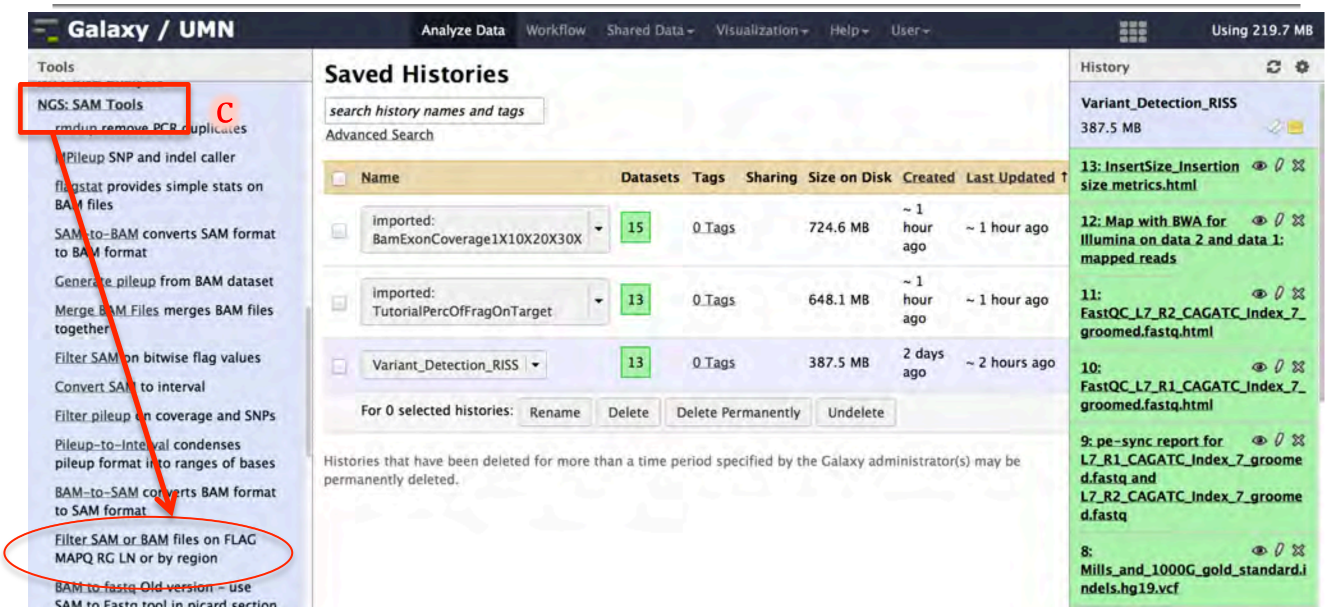

d) SAM/BAM dataset to generate statistics for: -> "....Map with BWA for Illumina on data ......"
e) Minimum MAPQ quality score: -> 1
f) Filter on bitwise flag: -> "yes"
g) In the *center pane*, scroll down to the section "**Only output alignments with all of these flag bits set:** and check boxes next to
    ✓ The read is mapped in a proper pair
h) Click "Execute"

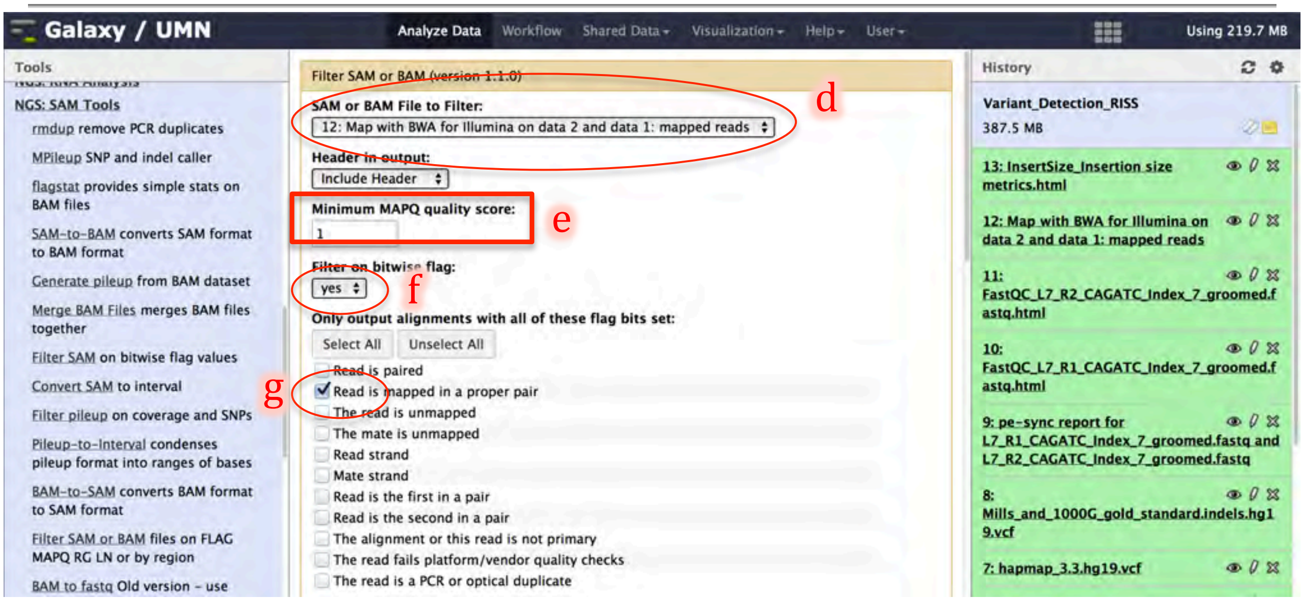

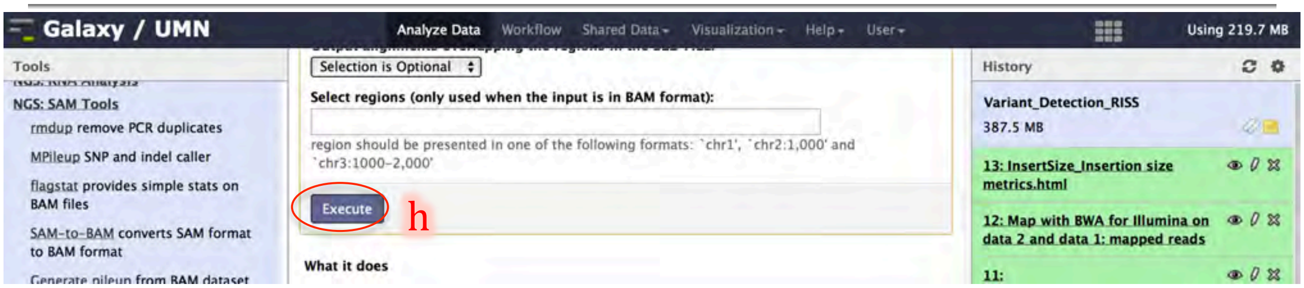

5.2 Sorting Reads and updating mate-pair information

a) Load *paired-read mate fixer* tool from the tool pane: "NGS: Picard (beta) -> Paired Read Mate Fixer for paired data"
b) SAM/BAM dataset to fix: -> "....Filter SAM or BAM ..."
c) Sort order: -> Coordinate sort
d) Output BAM instead of SAM: -> check (✓)
e) Click "Execute"

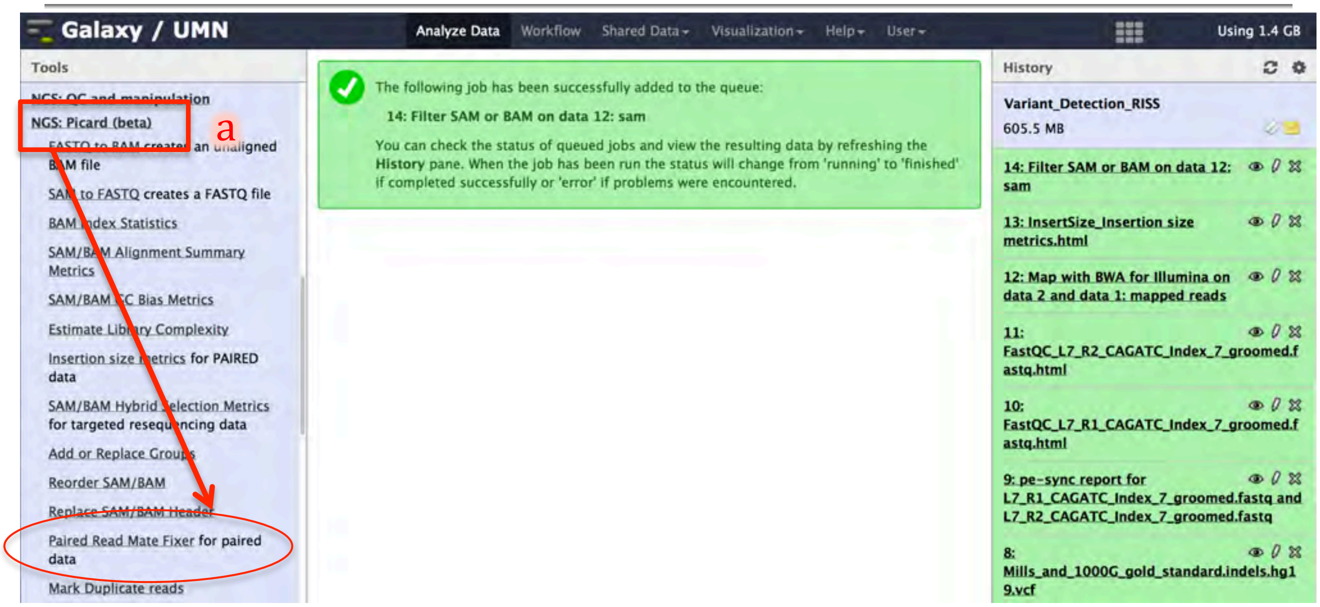

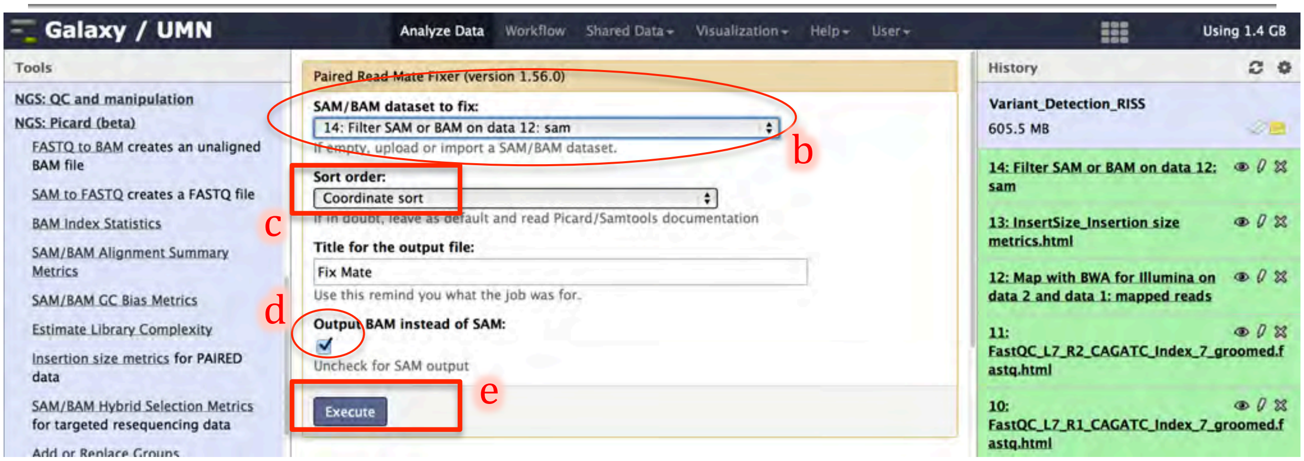

5.3 Removal of duplicates

a) Load *mark duplicates* tool from the tool pane: "NGS: Picard (beta) -> Mark Duplicate reads"
b) SAM/BAM dataset to mark duplicates in: -> ".....Paired Read Mate Fixer on data...."
c) Remove duplicates from output file: -> check (✓)
d) Assume reads are already ordered: -> check (✓)
e) Click "Execute"

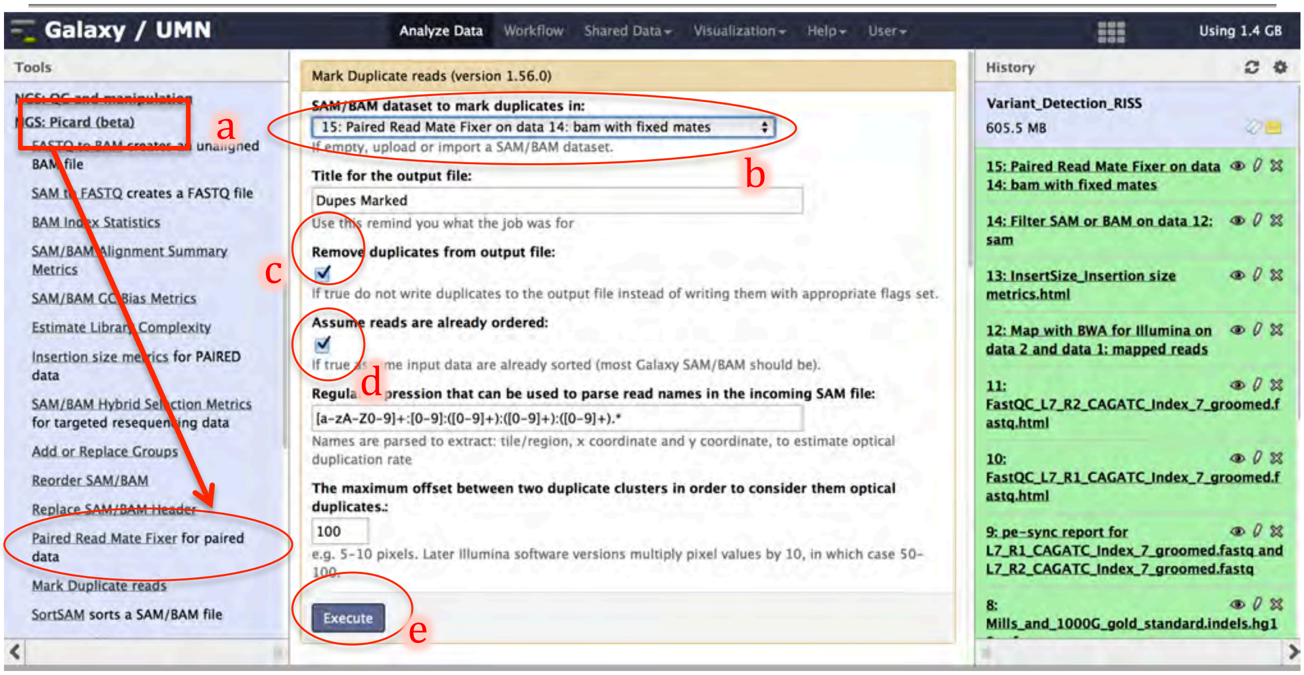

INDEL Realignment

5.4 Create Targets for Realignment

a) Load *realigner target creator* tool from the tool pane: “NGS: GATK Tools -> Realigner Target Creator for use in local realignment”
b) BAM file: -> “....MarkDups_Dupes Marked.bam”
c) Using reference genome: -> Homo sapiens hg19_canonical (GATK)
d) Click the “Add new Binding for reference-ordered data” button

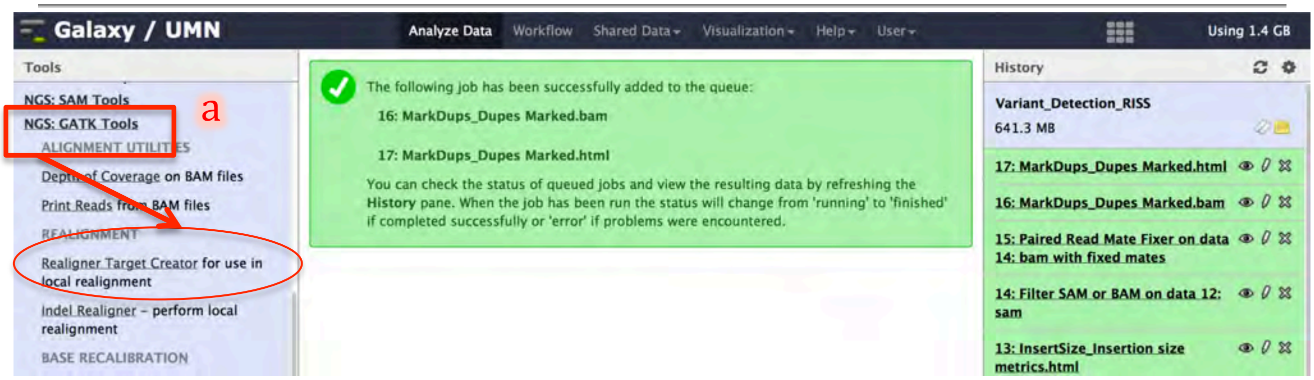

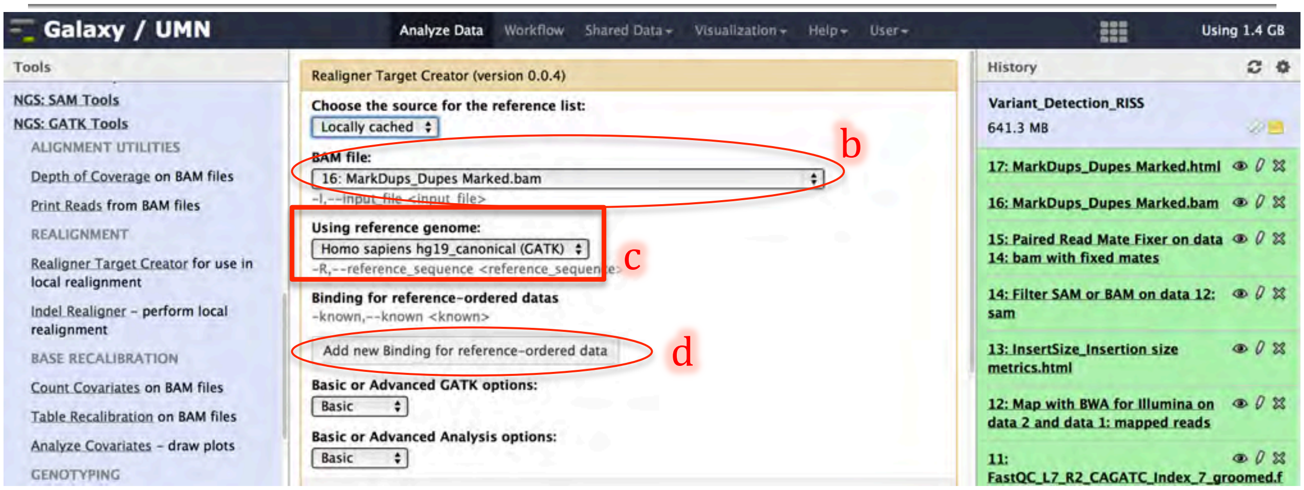

e) Under “**Binding Type:**” select “dbSNP”
f) Under “**ROD file:**” select “...dbsnp_137.hg19.vcf”
g) Click the “Add new Binding for reference-ordered data” button again
h) Under “**Binding Type:**” select “INDELs”
i) Under “**ROD file:**” select “...Mills_and_1000G_gold_standard.indels.hg19.vcf”
j) On the drop down menu below “**Basic or Advanced GATK options:**” select “Advanced”
k) Click the “Add new Operate on Genomic intervals” button

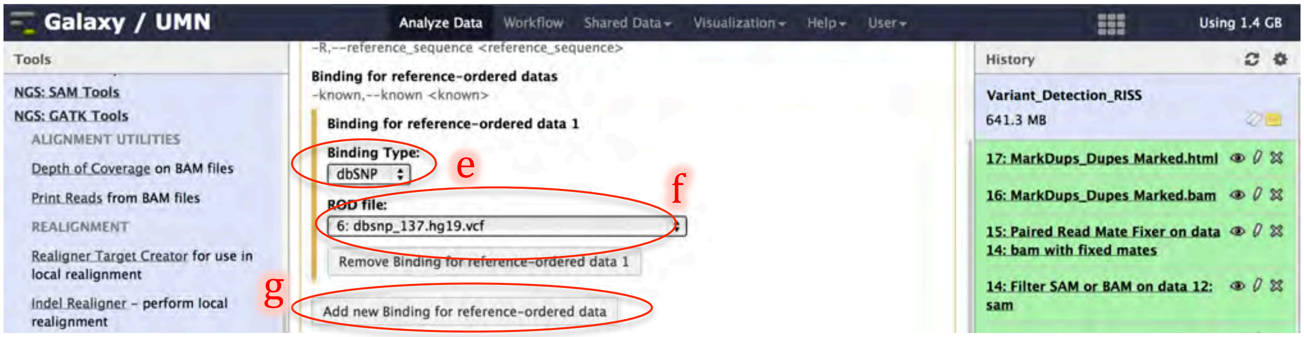

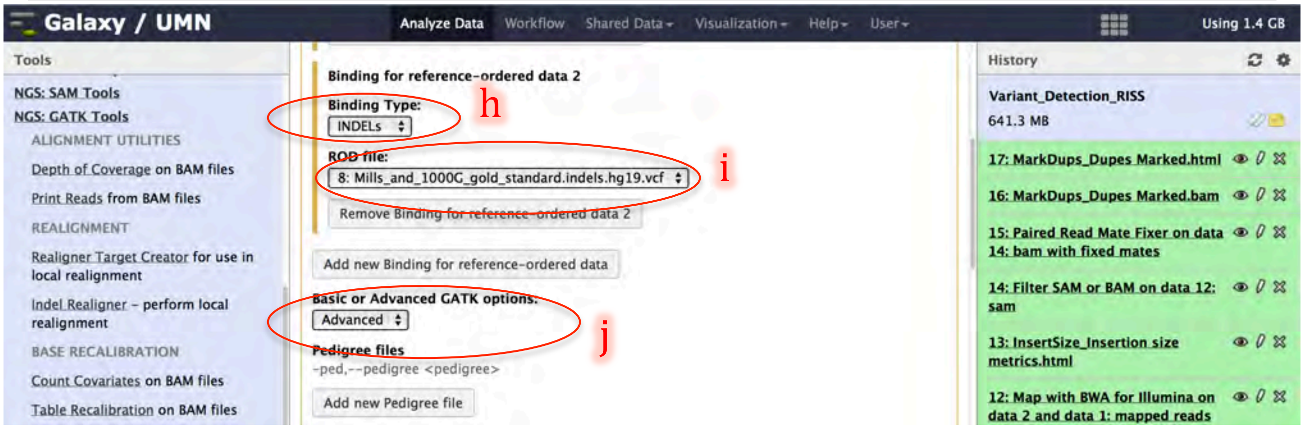

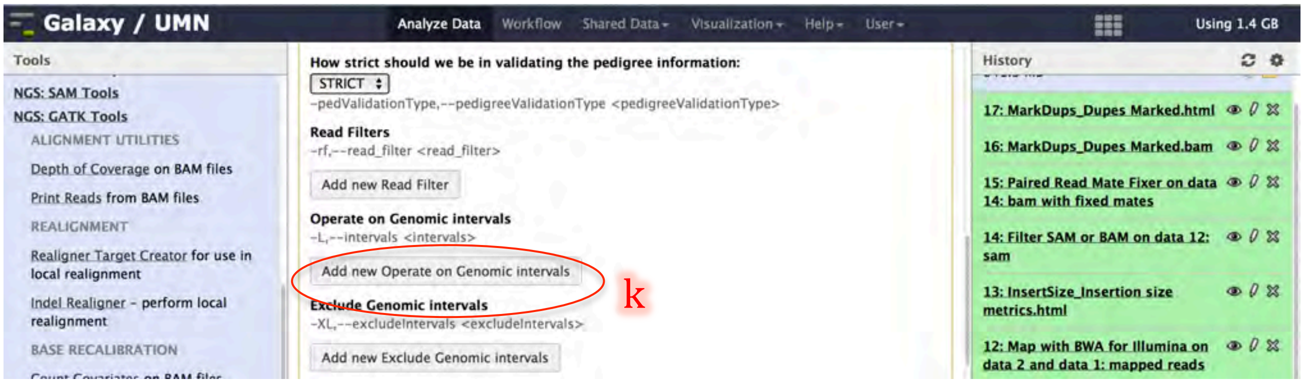

l) Under “**Genomic intervals:**” select the file “tutorial_exons.bed “

m) Scroll down and and the drop down menu below “**Basic or Advanced Analysis options:**” select “Advanced”

n) Fraction of base qualities needing to ……. to have high entropy (mismatchFraction): -> 0

o) Click “Execute”

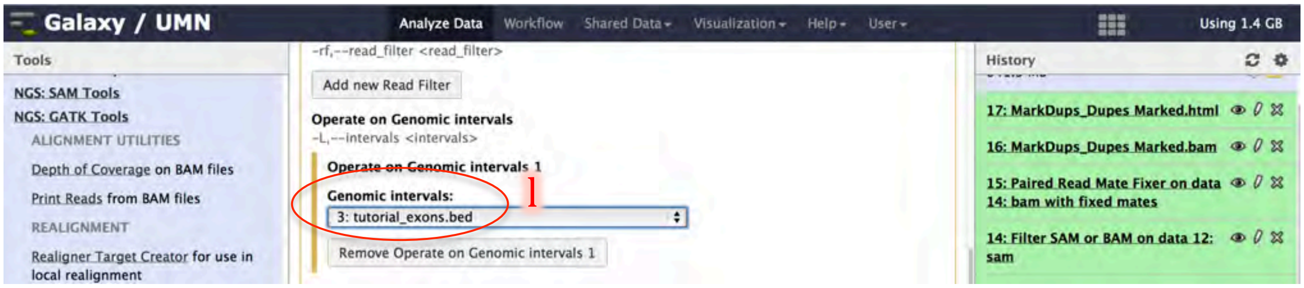

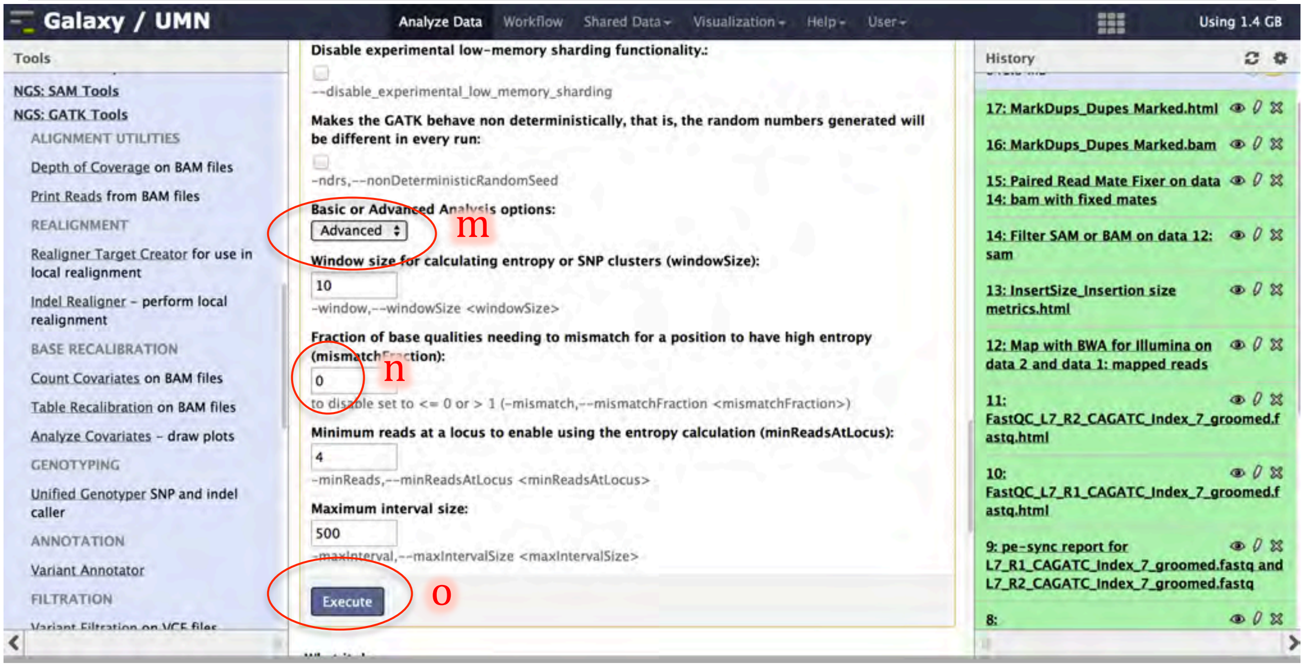

5.5 Realign reads around INDELS

a) Load *indel realigner* tool from the tool pane: "NGS: GATK Tools -> Indel Realigner - perform local realignment"
b) BAM file: -> "....MarkDups_Dupes Marked.bam"
c) Using reference genome: -> Homo sapiens hg19_canonical (GATK)
d) Restrict realignment to provided intervals: -> "....Realigner Target Creator on data....."
e) Click "Execute"

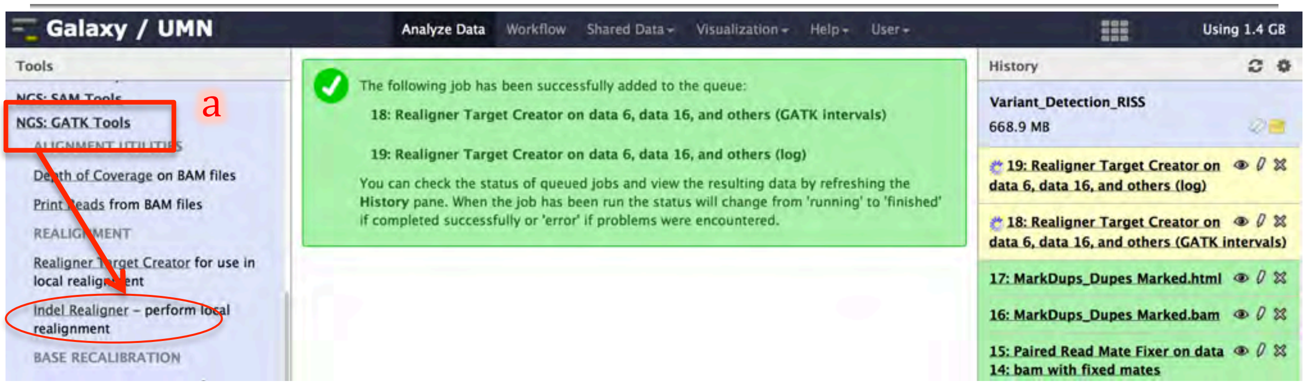

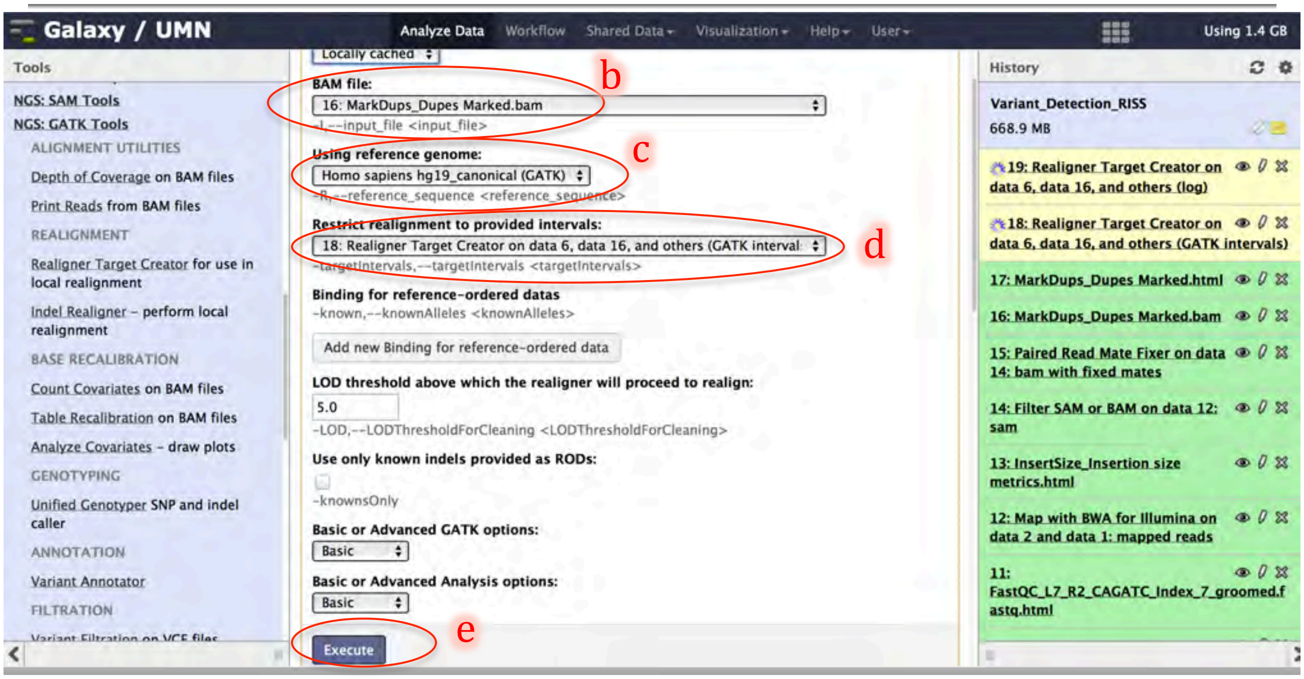

5.6 Remove duplicates after INDEL realignment

a) Load *mark duplicates* tool from the tool pane: “NGS: Picard (beta) -> Mark Duplicate reads”
b) SAM/BAM dataset to mark duplicates in: -> “....Indel Realigner.... (BAM)”
c) Remove duplicates from output file: -> check (✓)
d) Assume reads are already ordered: -> check (✓)
e) Click “Execute”

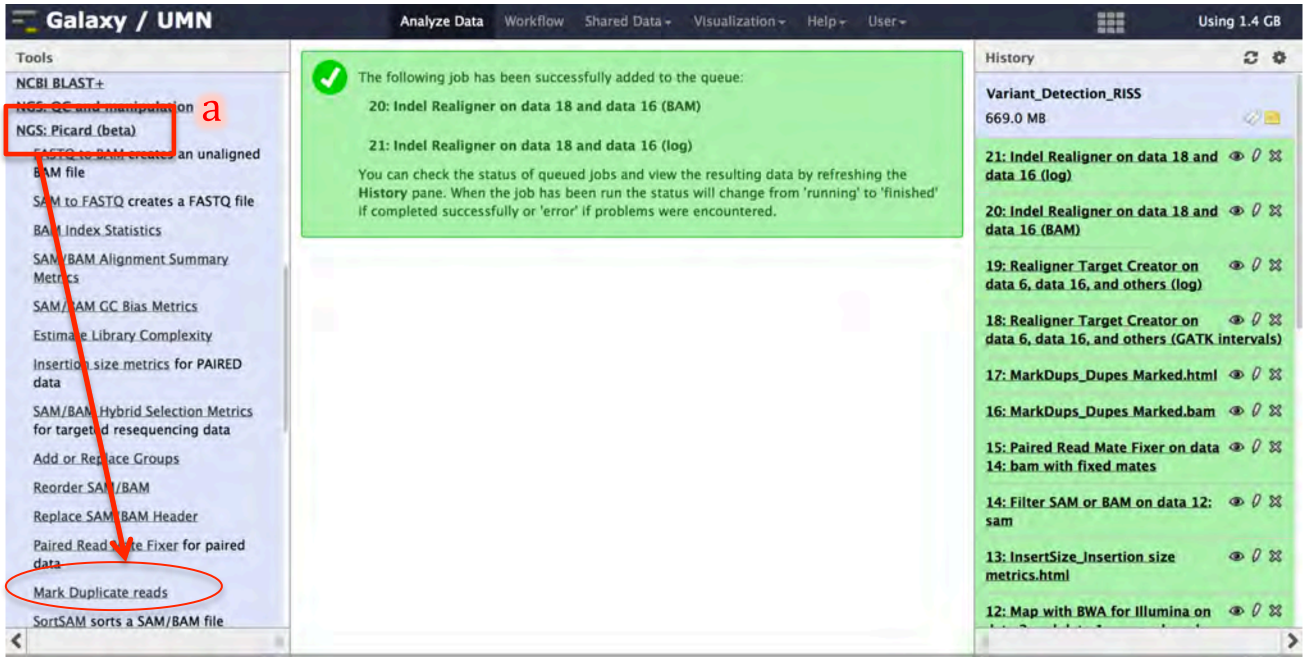

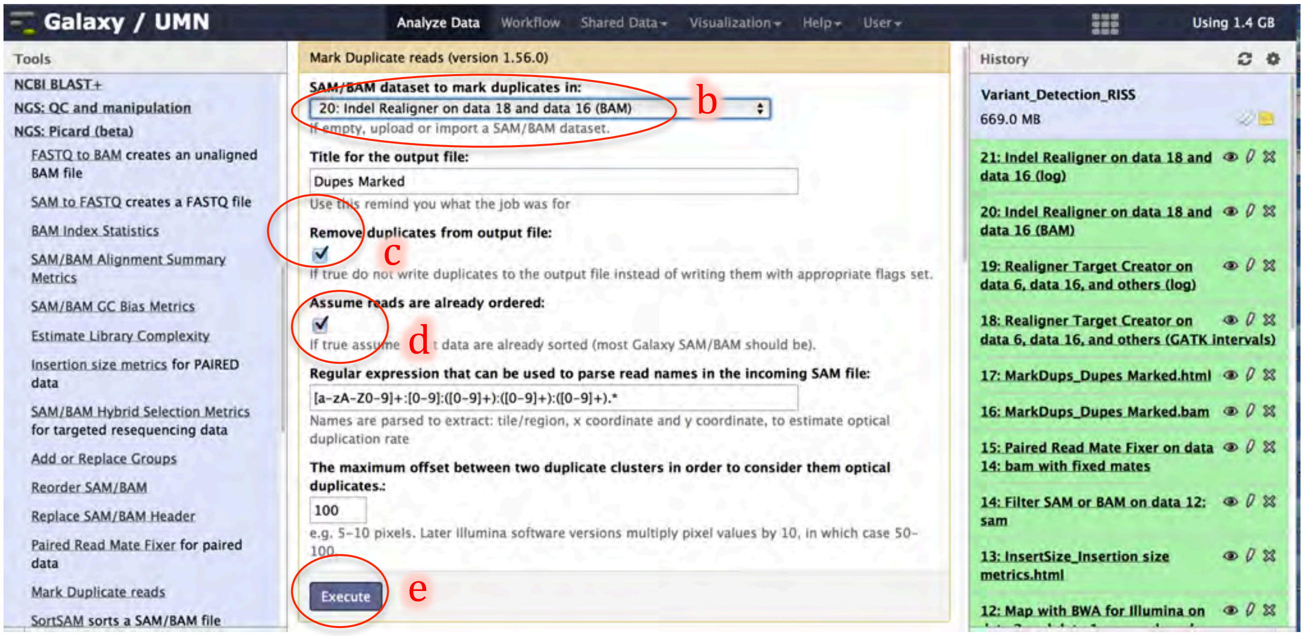

Base Quality Recalibration

5.7 Count Covariates (before base recalibration)

a) Load *count covariates* tool from the tool pane: “NGS: GATK Tools -> Count Covariates on BAM files”
b) BAM file: -> “…MarkDups_Dupes Marked.bam” (be sure to select the file generated after indel realignment)
c) Using reference genome: -> Homo sapiens hg19_canonical (GATK)
d) Covariates to be used in the recalibration: -> check boxes next to
   - ✓ ReadGroupCovariate
   - ✓ QualityScoreCovariate
   - ✓ CycleCovariate
   - ✓ DinucCovariate

e) Click the “Add new Binding for reference-ordered data” button

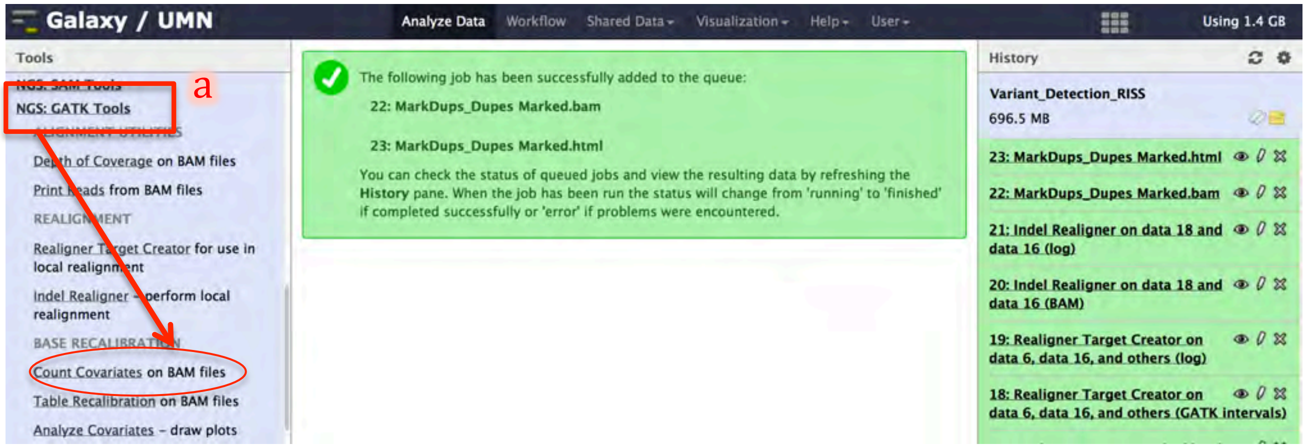

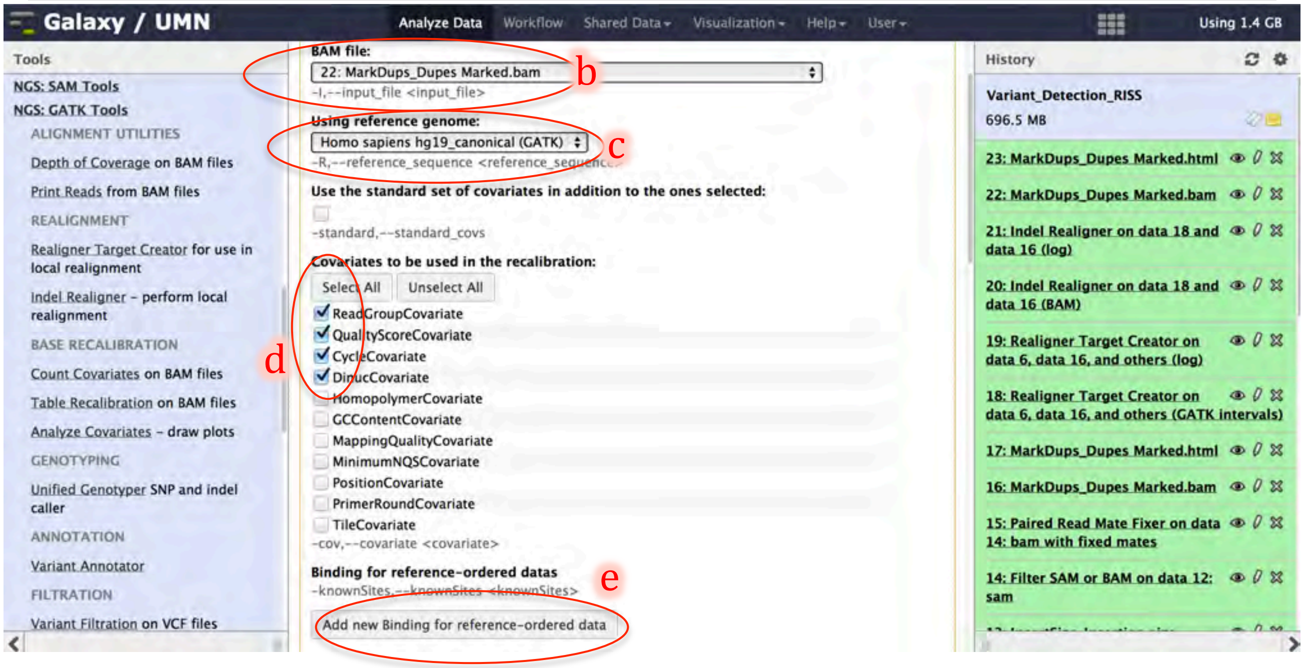

f) Binding Type: -> dbSNP
g) ROD file: -> dbsnp_137.hg19.vcf
h) Click "Execute"

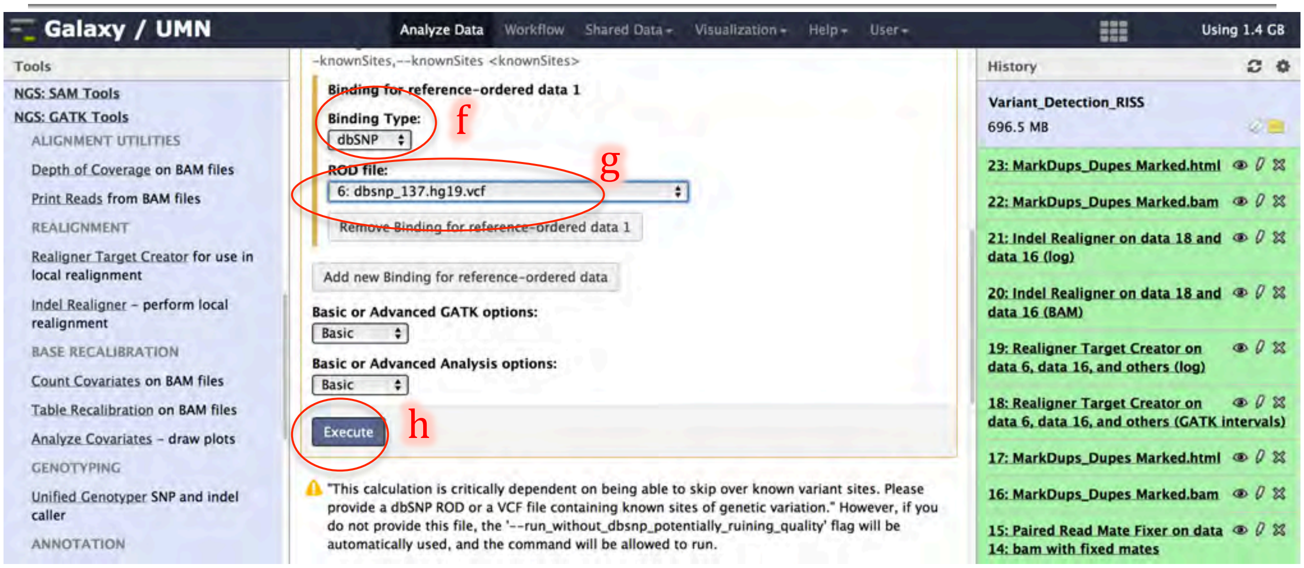

5.8 Analyze Covariates (before base recalibration)

a) Load *analyze covariates* tool from the tool pane: "NGS: GATK Tools -> Analyze Covariates - draw plots"
b) Covariates table recalibration file: -> "Count covariates…."
c) Click "Execute"

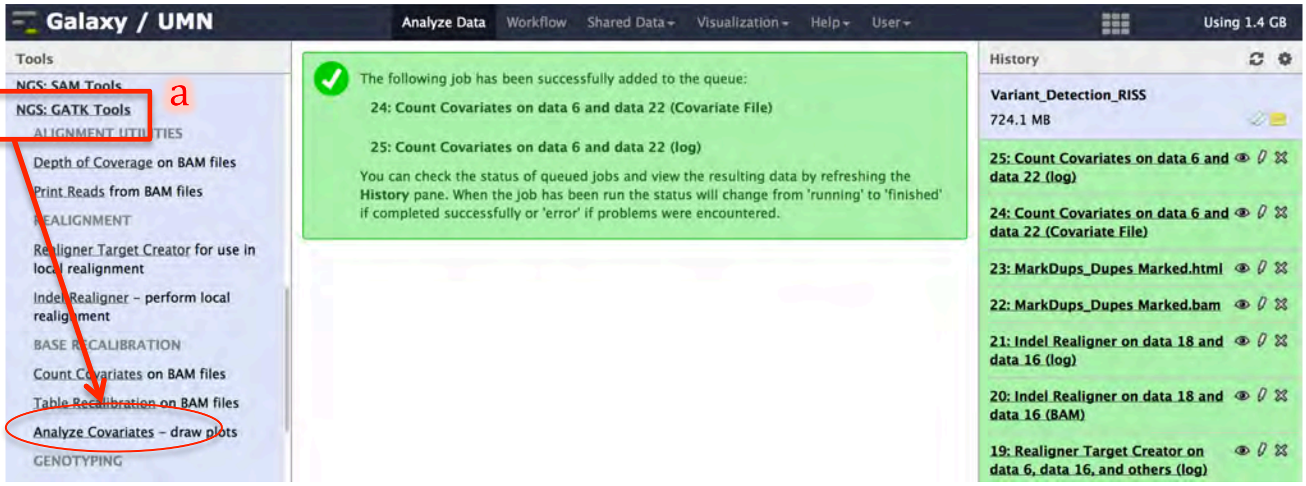

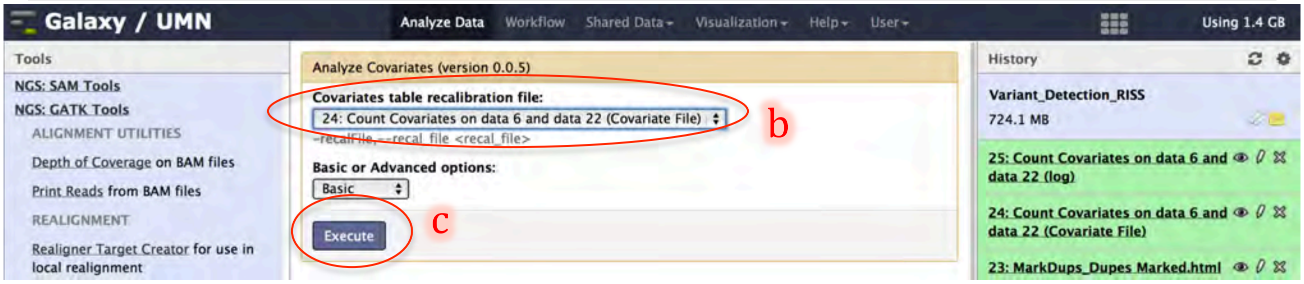

5.9 Review Covariate plots (before base quality recalibration)

Before recalibrating the base quality scores, it is prudent to first examine if reported and empirical scores agree. If they do, there is no need to recalibrate.

a) In the history pane click the eye icon next to the name of the *analyze covariates* HTML output file to display the file in the center pane
b) Click on the pdf output file *"NA_10858_400.QualityScoreCovariate.dat.quality_emp_v_stated. pdf"* showing the difference between reported and empirical base calls as a function of the different covariate
c) Inspect plot to determine if recalibration is necessary

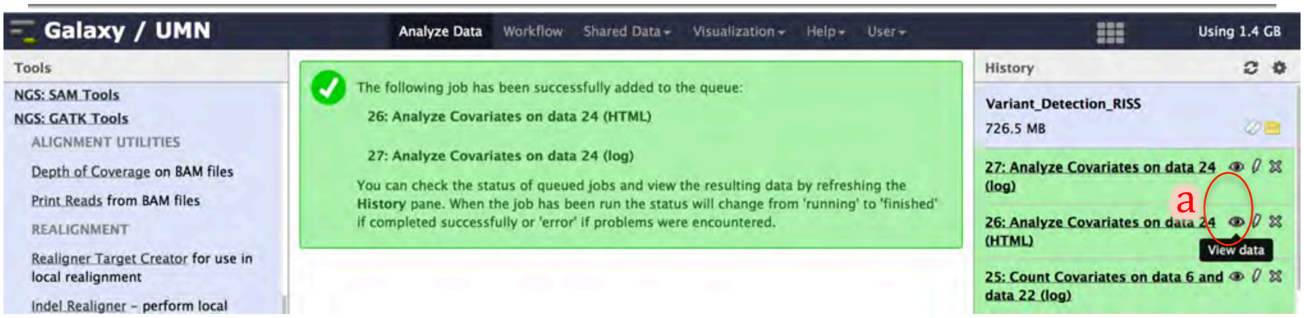

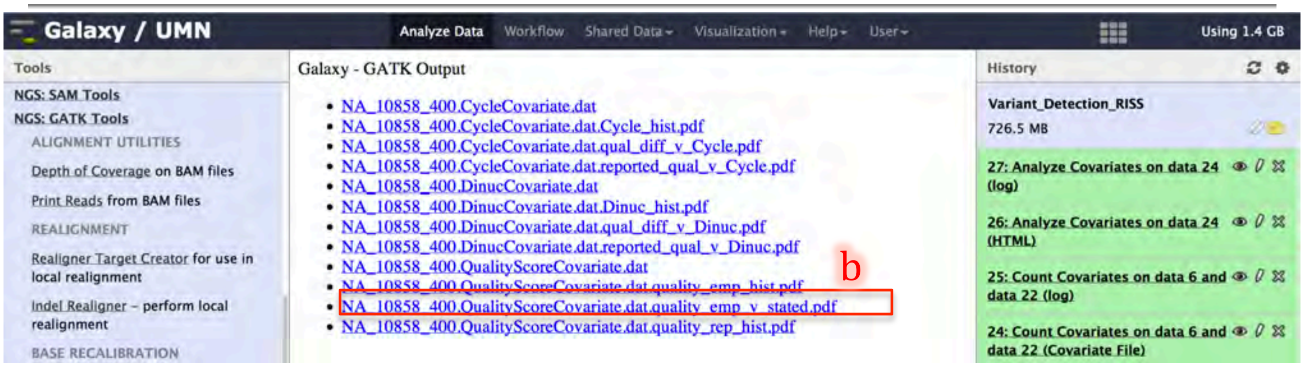

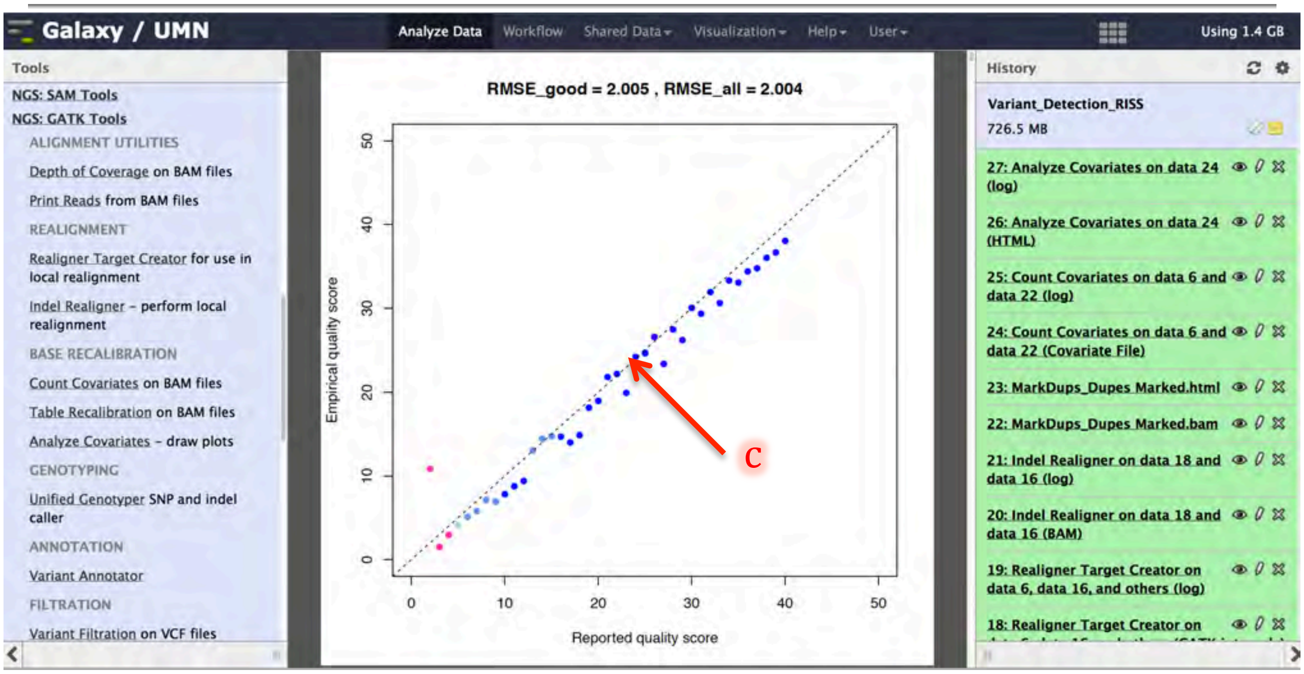

5.10 Recalibrate base quality scores

a) Load *Table Recalibration* tool from the tool pane: “NGS: GATK Tools -> Table Recalibration on BAM files”
b) Covariates table recalibration file: -> “…Count covariates….”
c) BAM file: -> “…MarkDups_Dupes Marked.bam” (be sure to select the file generated after indel realignment)
d) Using reference genome: -> Homo sapiens hg19_canonical (GATK)
e) Click “Execute”

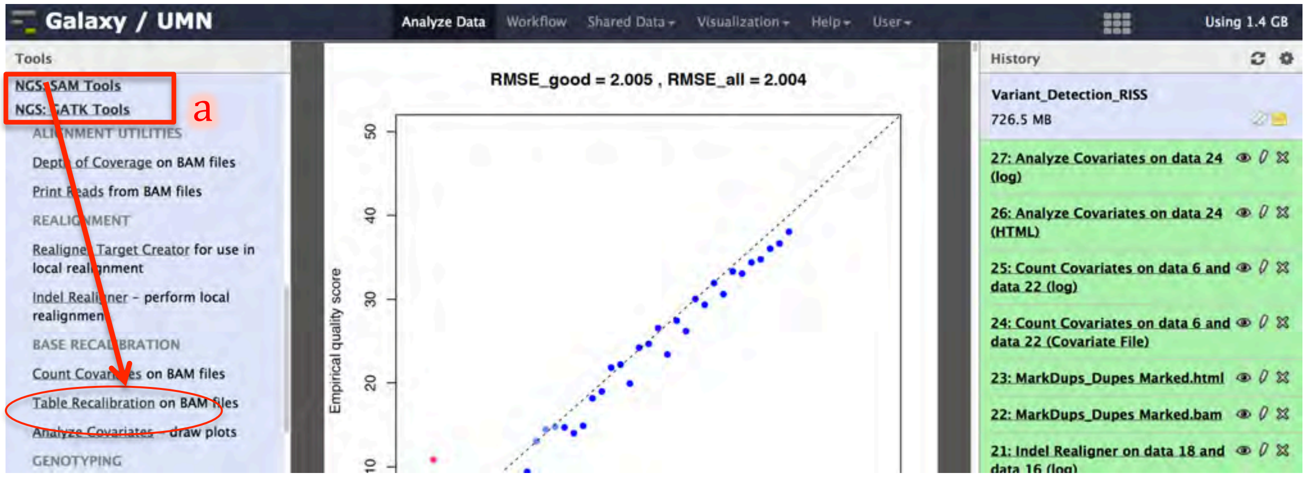

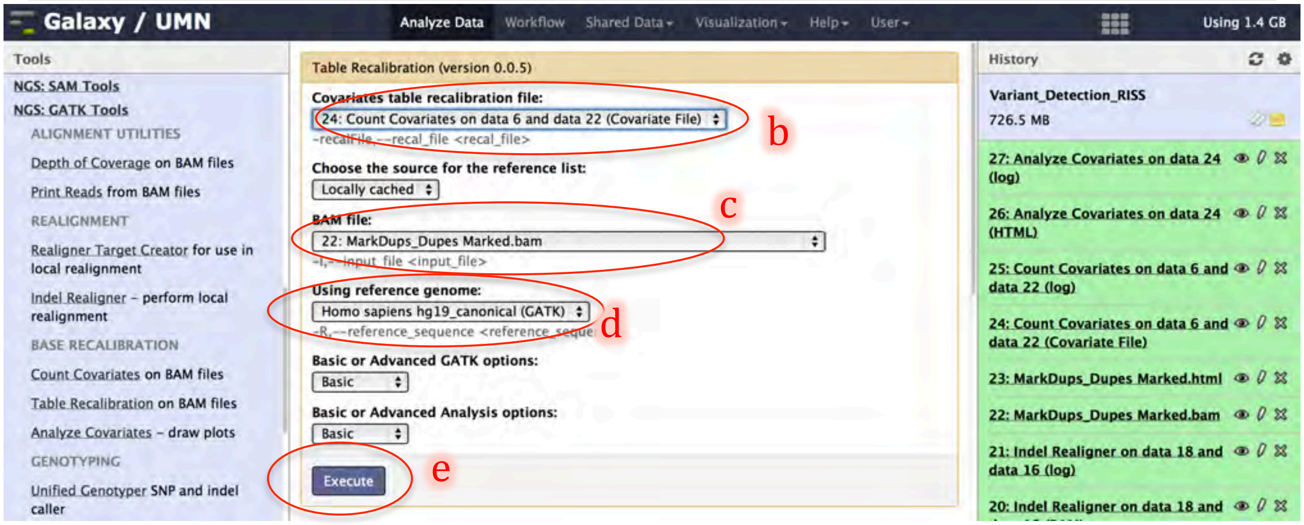

5.11 Count Covariates (after base recalibration)

a) Load *count covariates* tool from the tool pane: “NGS: GATK Tools -> Count Covariates on BAM files”
b) BAM file: -> “…Table Recalibrated…..”
c) Using reference genome: -> Homo sapiens hg19_canonical (GATK)
d) Covariates to be used in the recalibration: -> check boxes next to
   - ✓ ReadGroupCovariate
   - ✓ QualityScoreCovariate
   - ✓ CycleCovariate
   - ✓ DinucCovariate
e) Click on the “Add new Binding for reference-ordered data” button

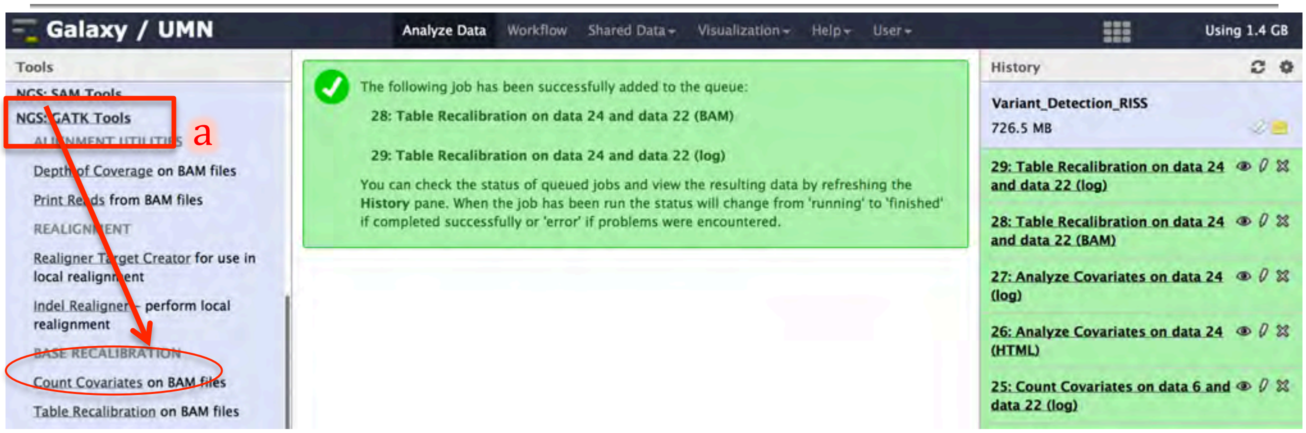

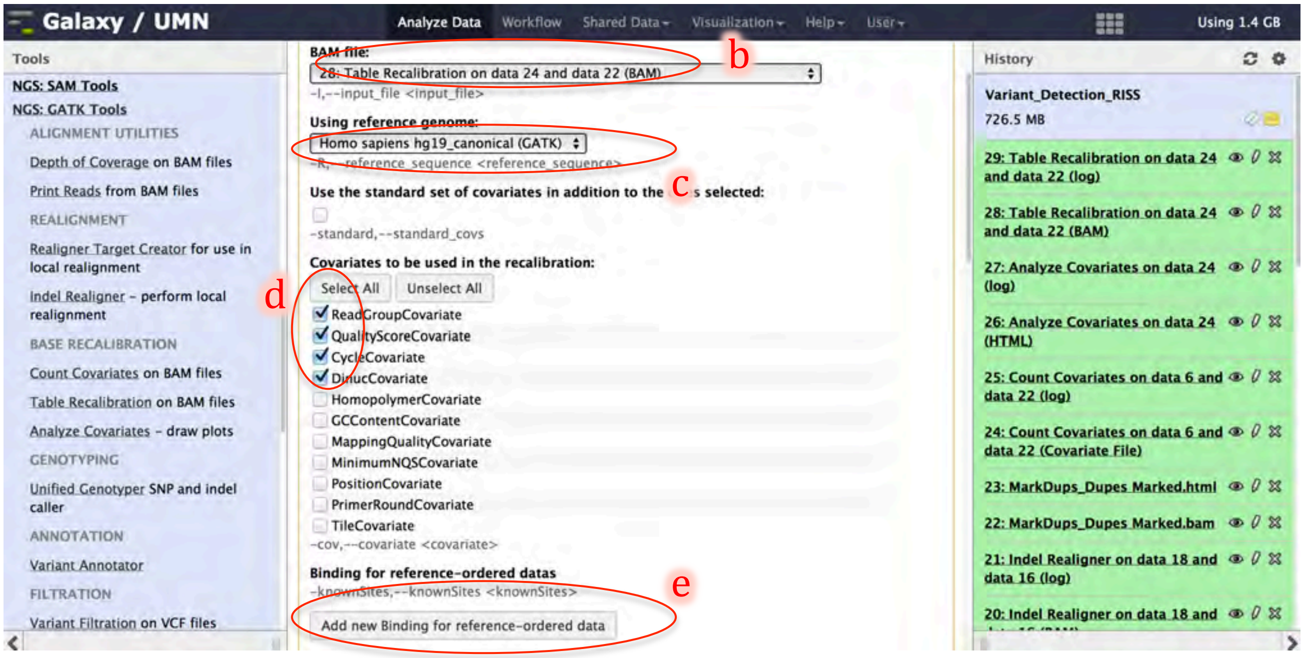

f) Binding Type: -> dbSNP
g) ROD file: -> dbsnp_137.hg19.vcf
h) Click "Execute"

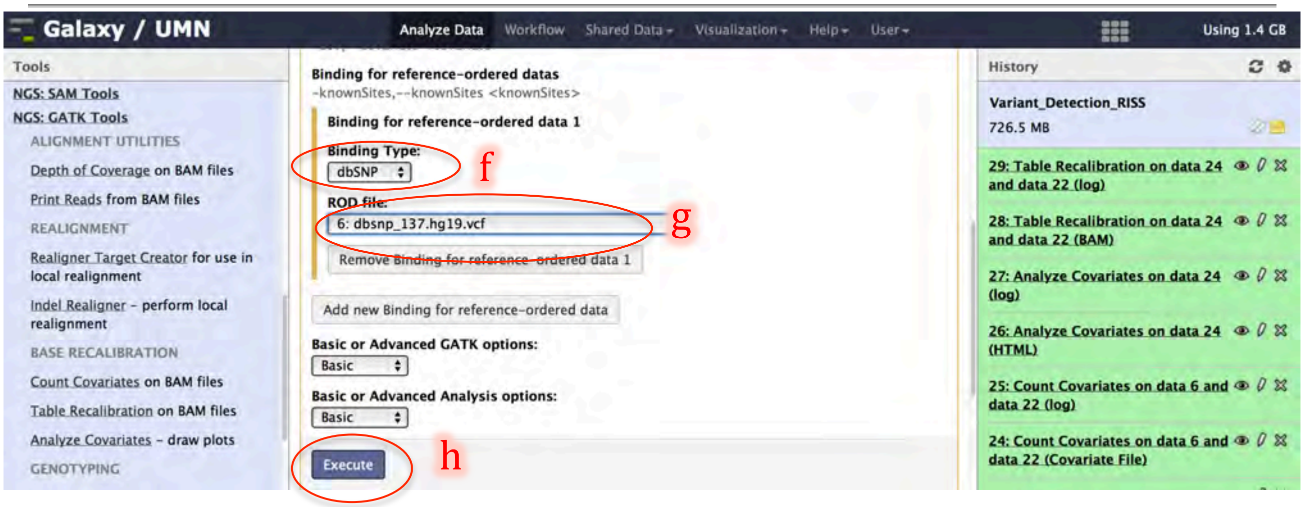

5.12 Analyze Covariates (after base recalibration)

a) Load *analyze covariates* tool from the tool pane: "NGS: GATK Tools -> Analyze Covariates - draw plots"
b) Covariates table recalibration file: -> "Count covariates…." (be sure to select the file generated after base quality recalibration)
c) Click "Execute"

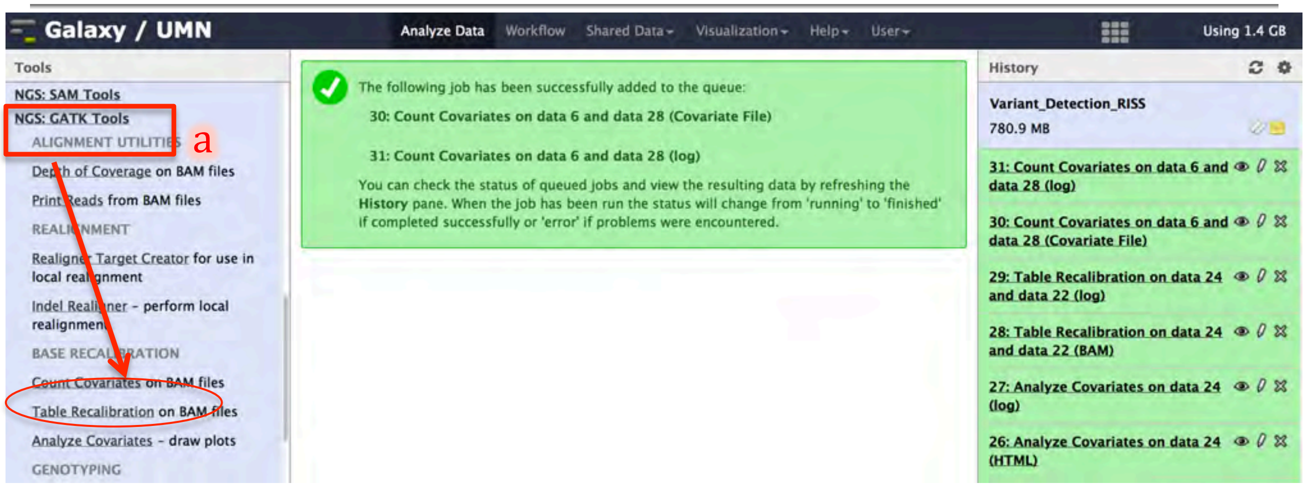

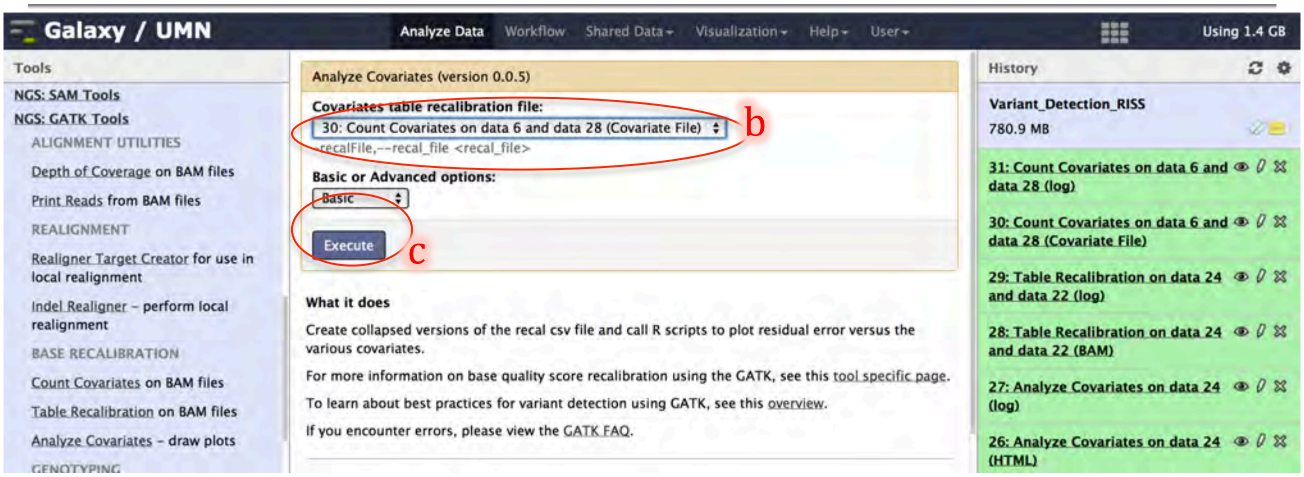

5.13 Review Covariate plots (after base quality recalibration)

a) In the history pane click the eye icon next to the name of the *analyze covariates* HTML output file to display the file in the center pane
b) Click on the pdf output file *"NA_10858_400.QualityScoreCovariate.dat.quality_emp_v_stated. pdf"* showing the difference between reported and empirical base calls as a function of the different covariate

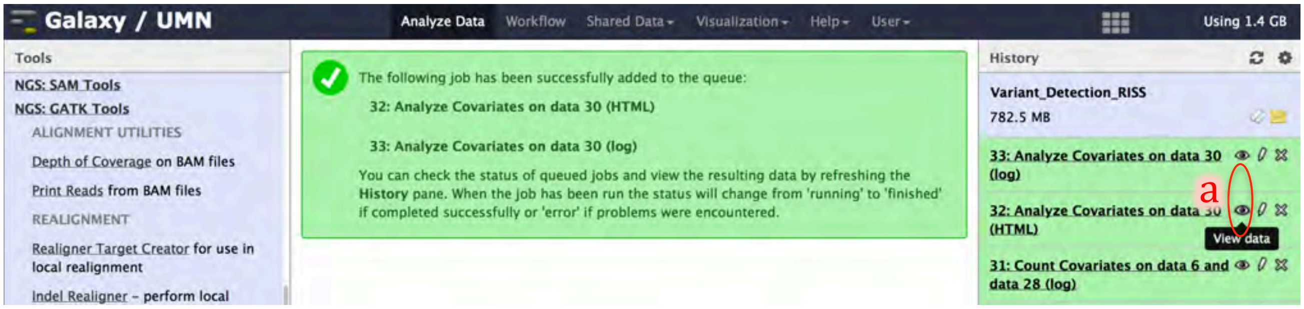

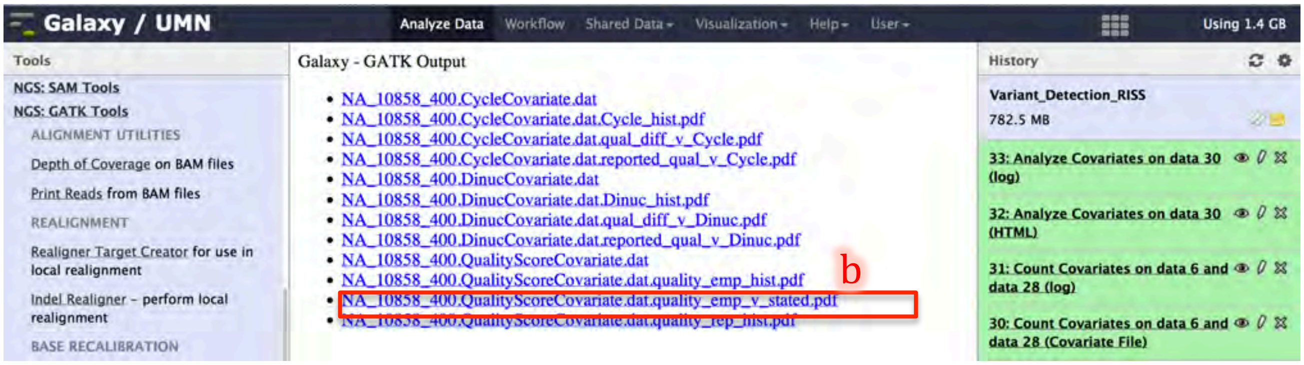

c) Examine plot to evaluate recalibration. Observe most improvement is in the Q30 - Q40 range
d) To determine why most improvement is in the Q30 –Q40 range, examine input data by clicking the eye icon next to the name of the *analyze covariates* HTML output file generated before base recalibration "Analyze…."
e) Click on the histogram showing distribution of reported quality score before recalibration "NA_10858_400.QualityScoreCovariate.dat.quality_rep_hist.pdf"

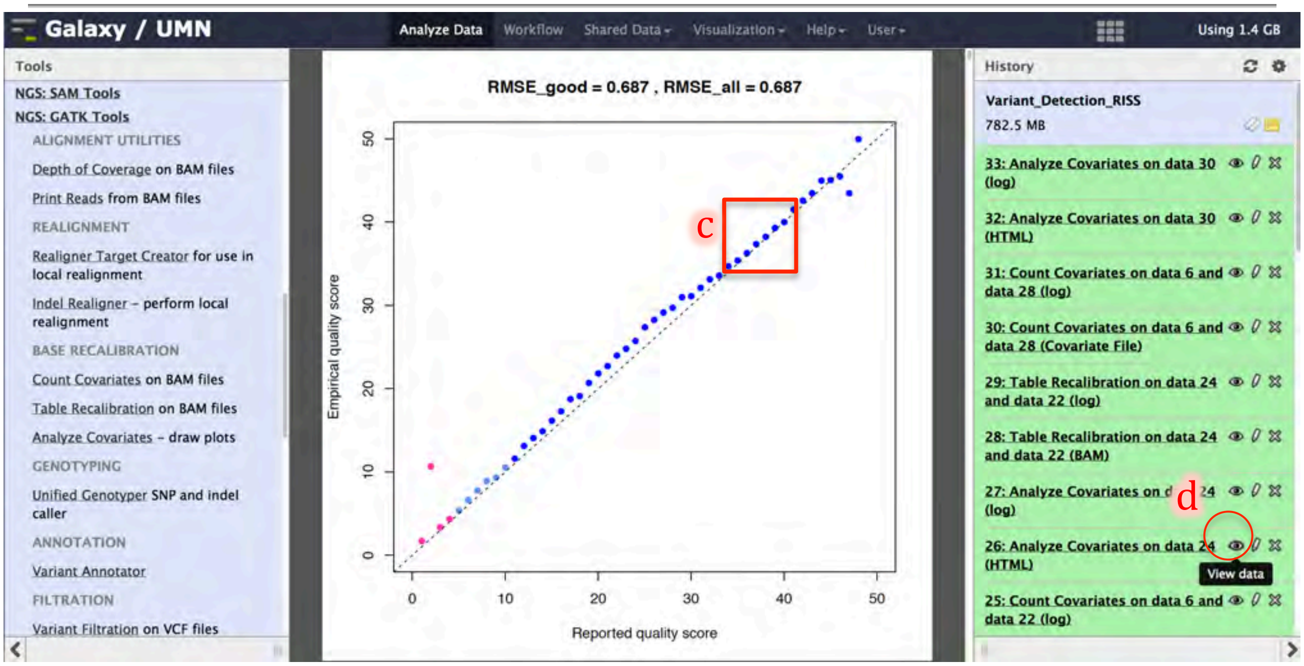

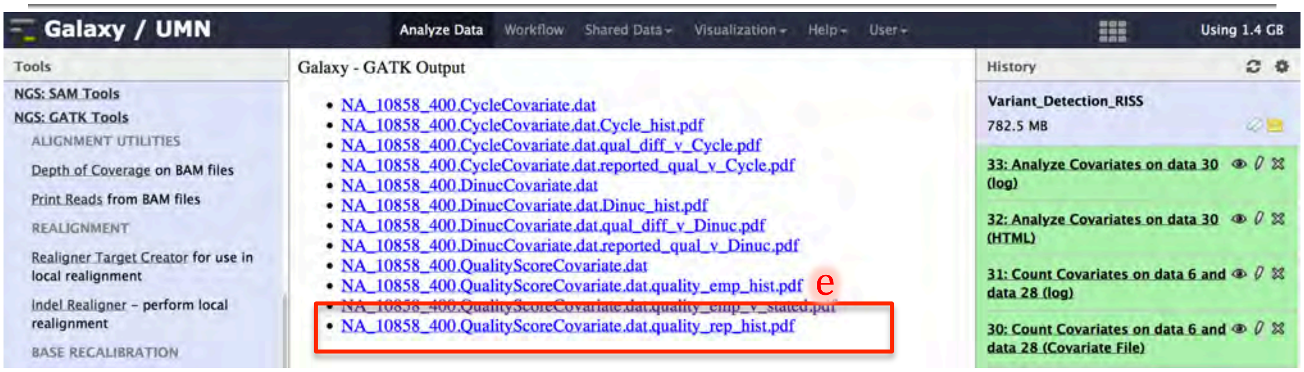

f) Observe input data consisted of scores in the Q30 to Q40 range. Recalibration only as good as training data

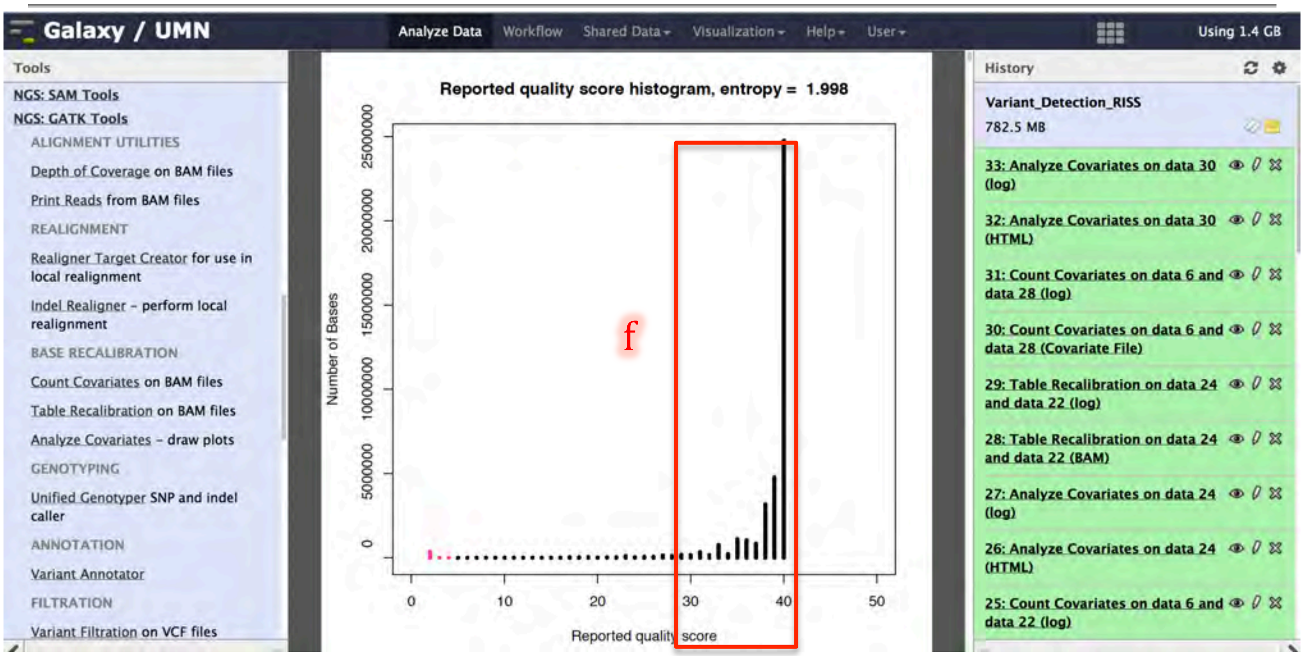

6 GATK Phase 2: Variant Discovery

★ GATK Phase 2 details
The GATK's Unified Genotyper employs a Bayesian model to compute the likelihood for each of the 10 possible bi-allelic diploid genotypes (AA, AC, AG, AT, CC,CG,CT,GG,GT,TT), as described in the equation below. The likelihood is computed across the entire pileup of bases at a position, taking into consideration the associated quality scores. Only "good bases" are considered – typically those satisfying a minimum base quality, read mapping quality, and pair mapping quality. In the formula below, L(G|D) is computed over all 10 possible genotypes. See http://www.broadinstitute.org/gatk/gatkdocs/org_broadinstitute_sting_gatk_walkers_genotyper_UnifiedGenotyper.html for more information.

• Parameter selection and considerations

1. It is useful to provide a reference dbSNP VCF file to the Unified Genotyper, as this will automatically transfer rsIDs from dbSNP onto known variants detected in your samples.
2. Genotype likelihood calculations can be performed for SNP, INDEL or BOTH.
3. Two separate variant call phred-scale quality values are reported: a threshold for *high-quality calling* variants and a potentially less stringent threshold for emitting/outputting lower-confidence borderline calls.
4. The final GATK phase discussed in the next section needs information about each of the variant calls in order to rank the confidence of each one (e.g., FisherStrand to assess strand bias). You will save time and effort by allowing the Unified Genotyper to track these ahead of time under "Annotation Types", rather than do this later.

6.1 Variant detection using Unified Genotyper

a) Navigate back to Galaxy and load *Unified Genotyper* tool from the tool pane: "NGS: GATK Tools -> Unified Genotyper SNP and indel caller"
b) BAM file: -> "...Table Recalibrated......(BAM)"
c) Using reference genome: -> Homo sapiens hg19_canonical (GATK)
d) Click the "Add new Binding for reference-ordered data" button

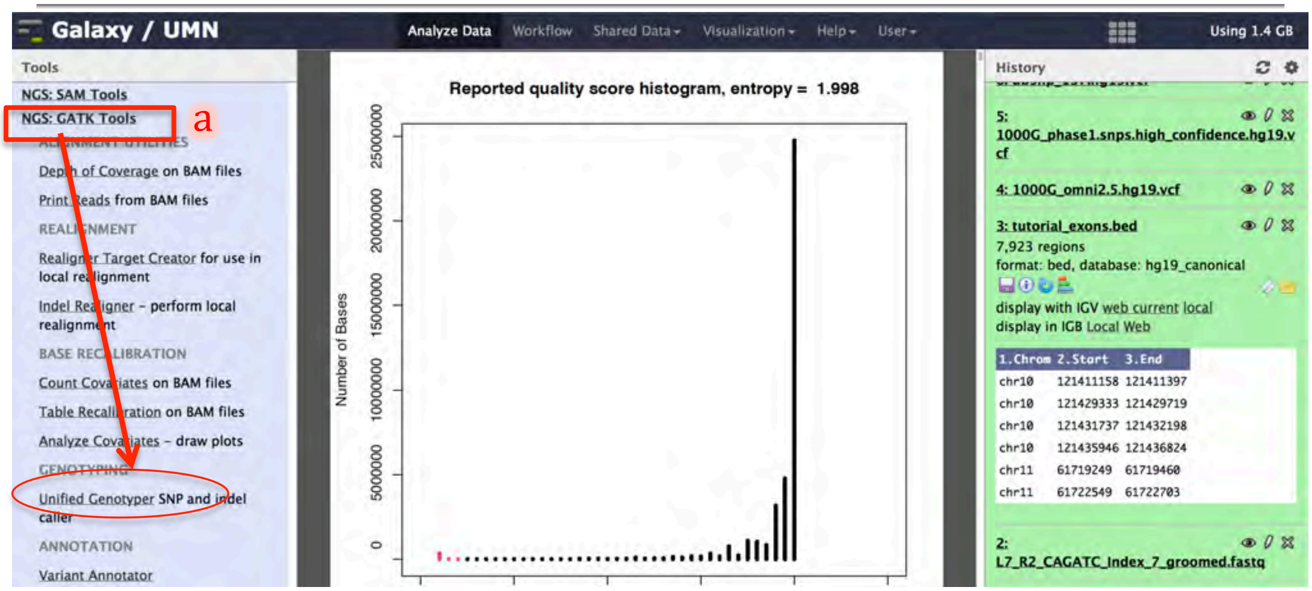

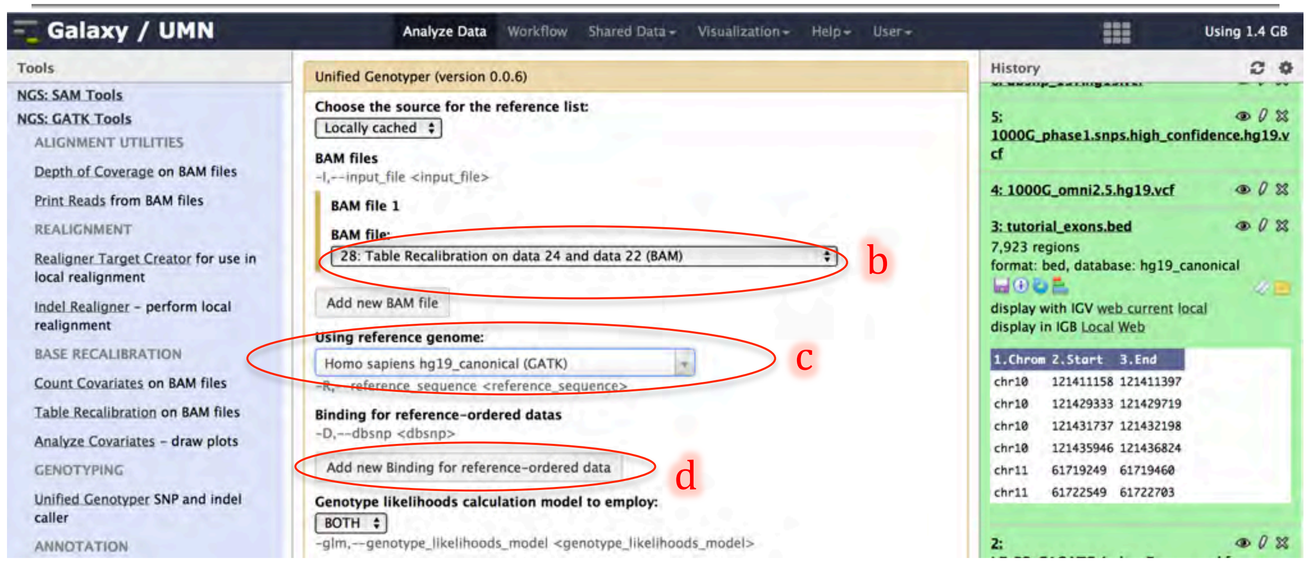

e) Binding Type: -> dbSNP
f) ROD file: -> dbsnp_137.hg19.vcf
g) Genotype likelihoods calculation model to employ: -> BOTH
h) The minimum phred-scaled confidence threshold at which variants not at 'trigger' track sites should be called: -> 20
i) The minimum phred-scaled confidence threshold at which variants not at 'trigger' track sites should be emitted (and filtered if less than the calling threshold): -> 20
j) On the drop down menu below "**Basic or Advanced GATK options:**" select "Advanced"
k) Click the "Add new Operate on Genomic intervals" button

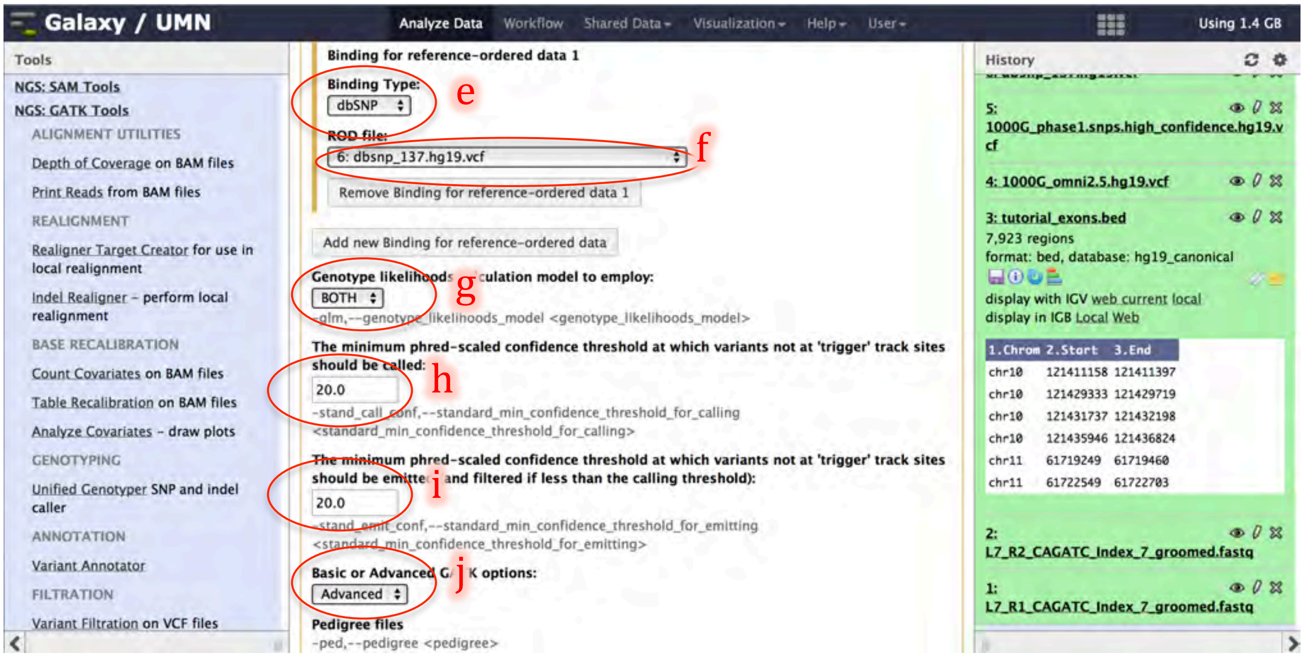

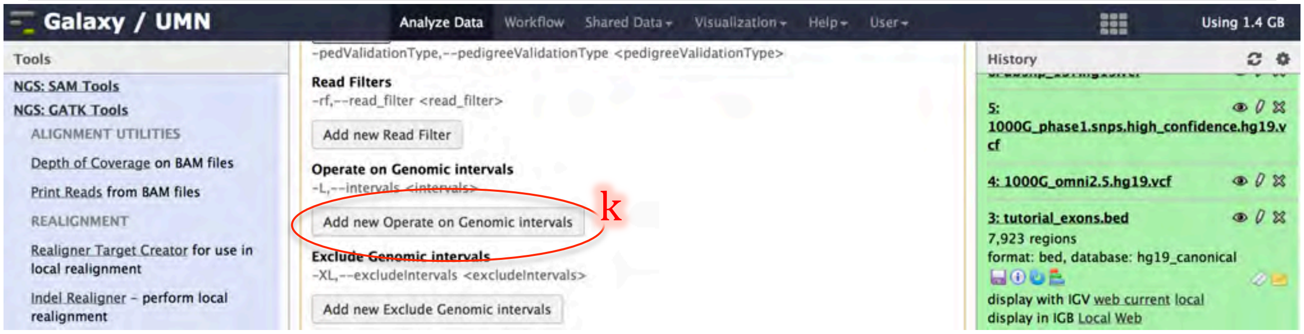

l) Under “**Genomic intervals:**” select the file “tutorial_exons.bed “

m) Basic or Advanced Analysis options: -> Advanced

n) Annotation Types: -> check boxes next to

- ✓ FisherStrand
- ✓ HaplotypeScore
- ✓ HomopolymerRun
- ✓ MappingQualityRankSumTest
- ✓ QualByDepth
- ✓ ReadPosRankSumTest

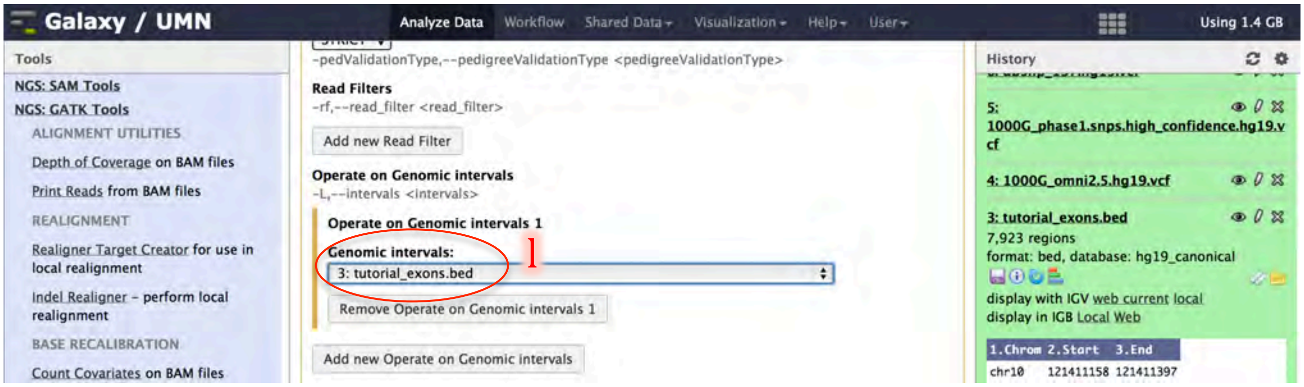

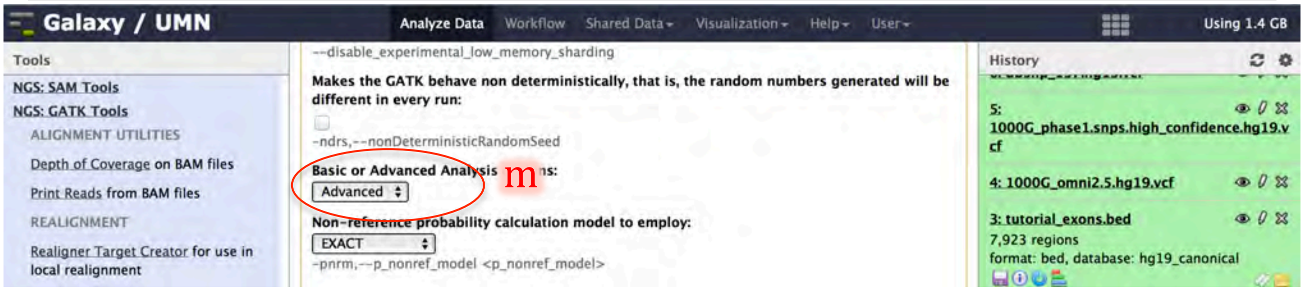

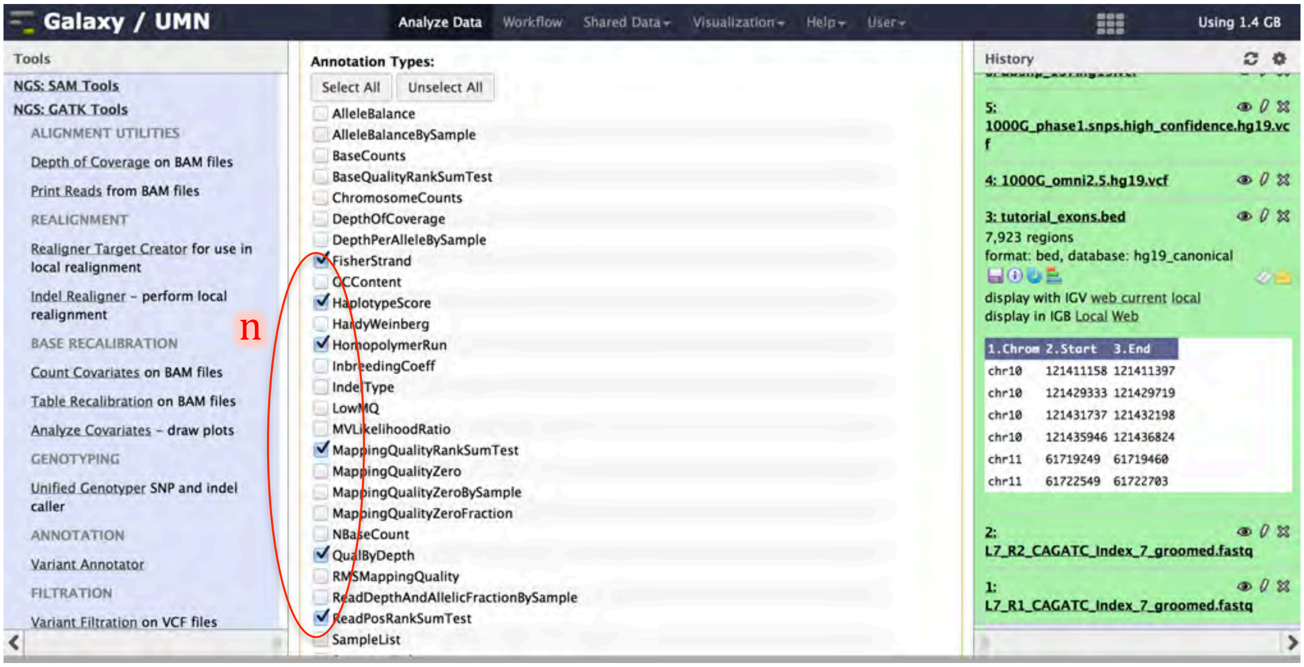

o) Annotation Interfaces/Groups: -> check box next to
   ✓ Standard

p) Click "Execute"

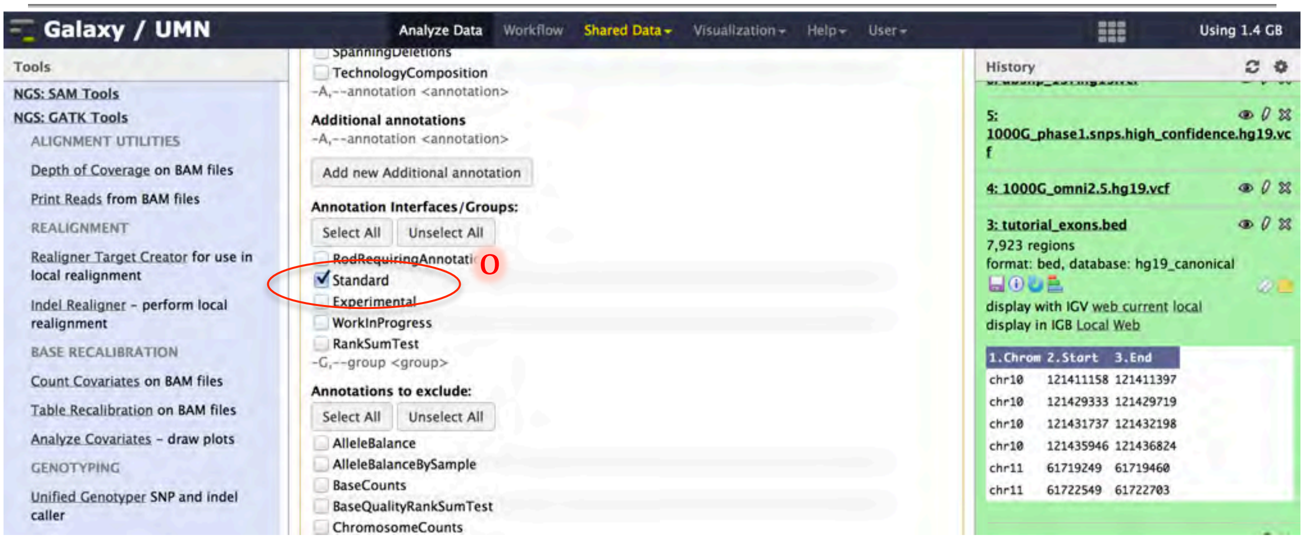

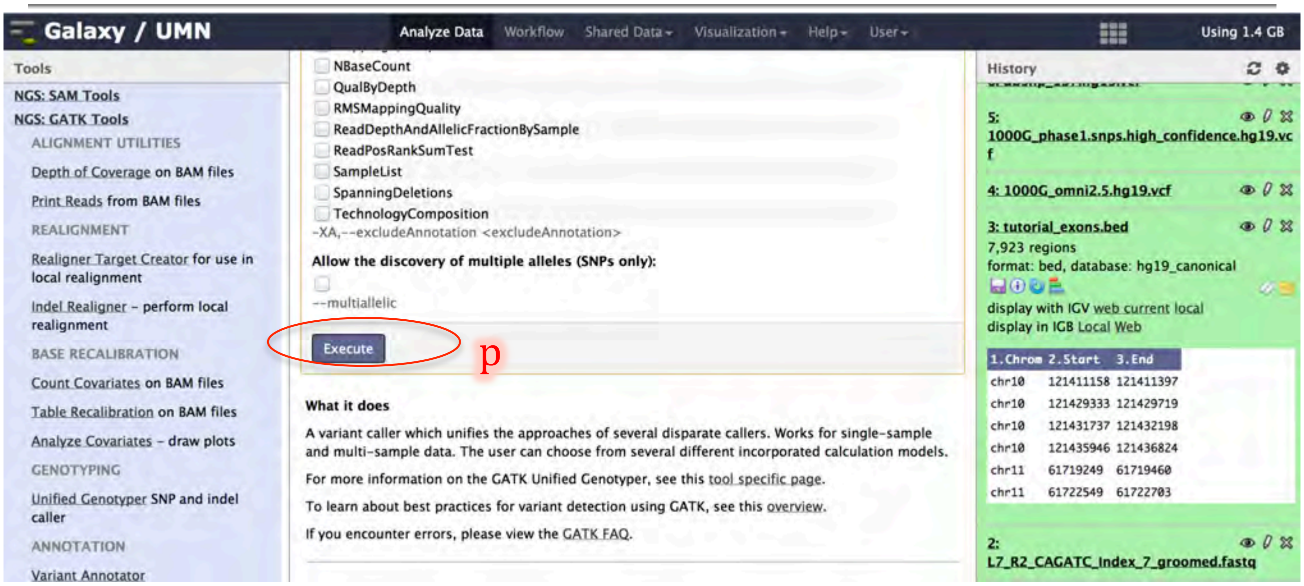

6.2 Review Unified Genotyper results (Raw Variants)

a) In the history pane click the eye icon next to the name of the *Genotyper* output file to display the file in the center pane
b) Click the arrow at the bottom of the *tools pane* to the left of the browser minimize it

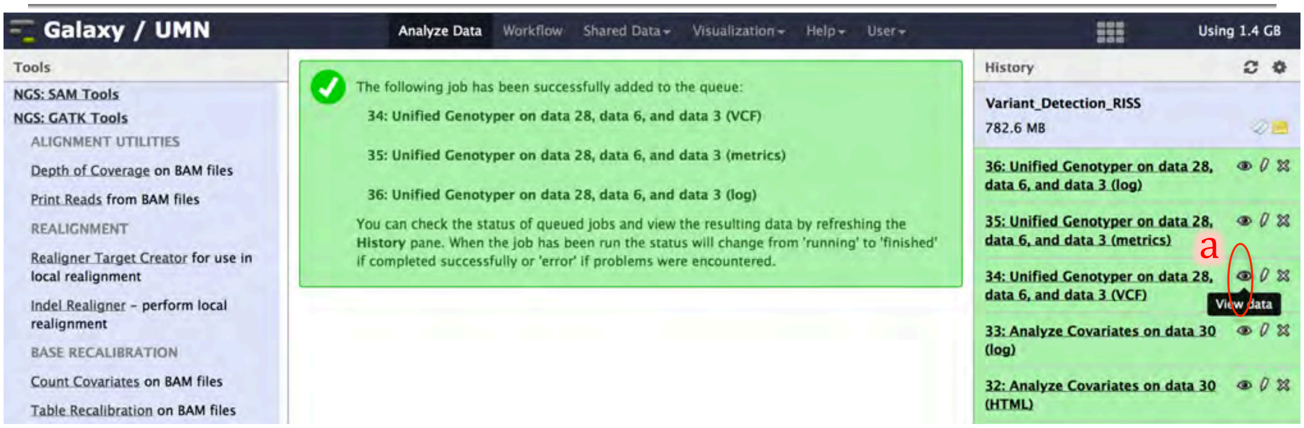

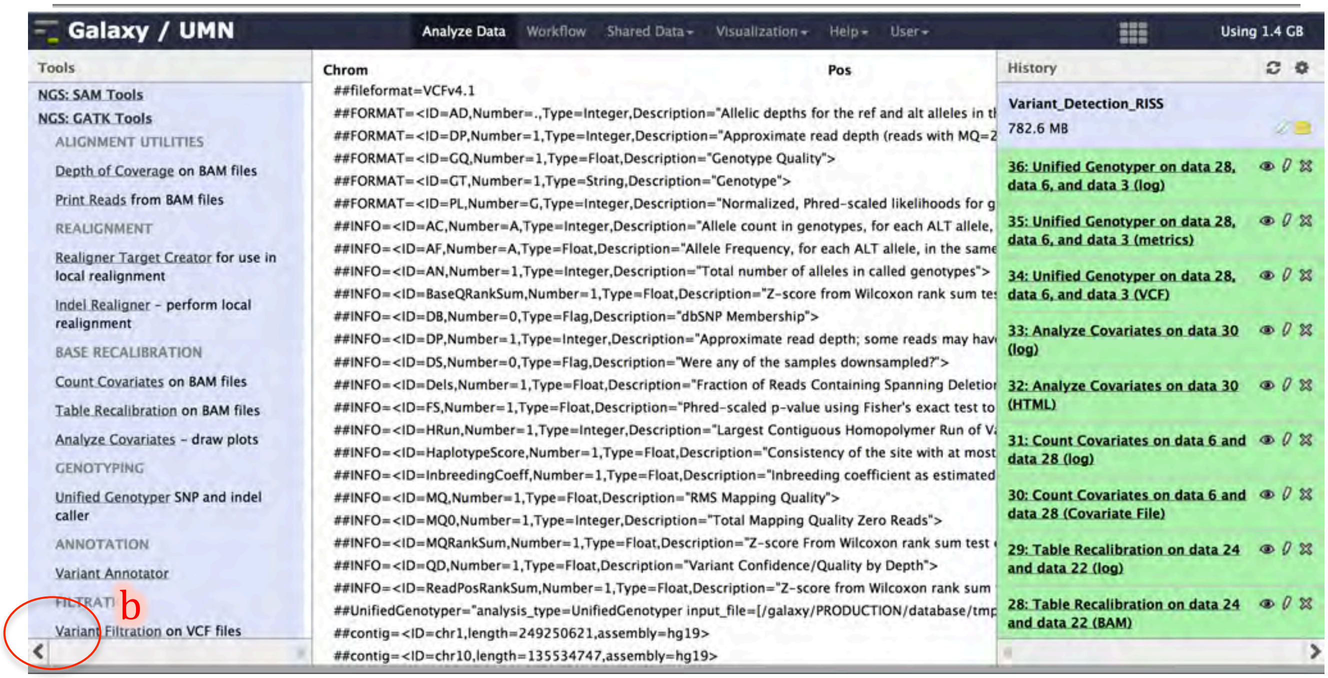

c) Click the arrow at the bottom of the *history pane* to the right of the browser minimize it
d) Scroll to the left and right of the *center pane* to view variants. NOTE: Browser not the ideal application for viewing results
e) Click the arrow at the bottom-left corner of your browser to bring the *tools pane* back to view

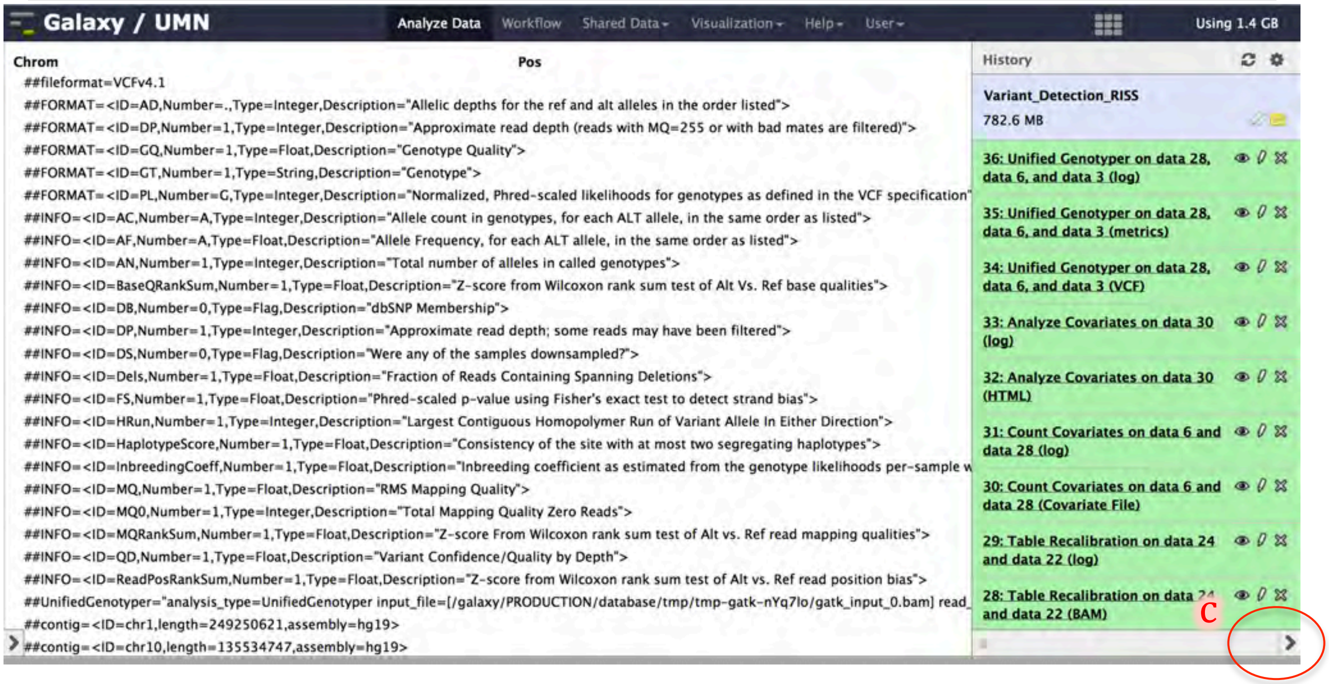

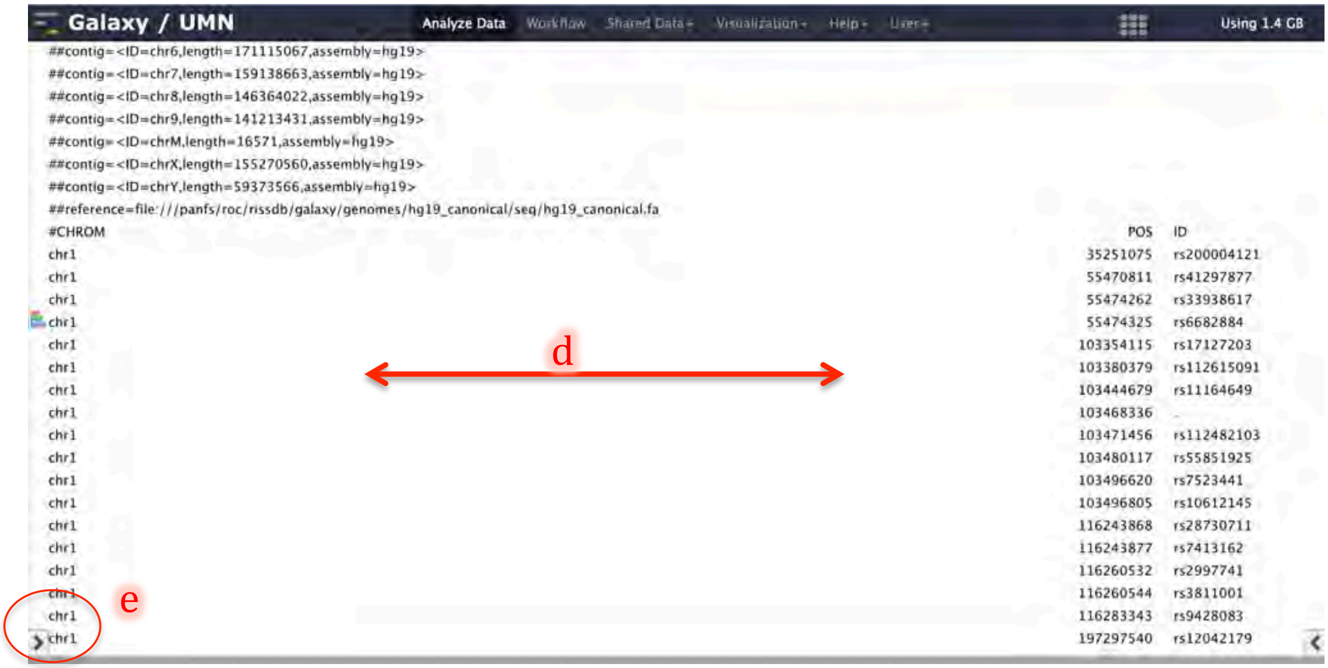

f) Click the arrow at the bottom-right corner of your browser to bring the *tools pane* back to view

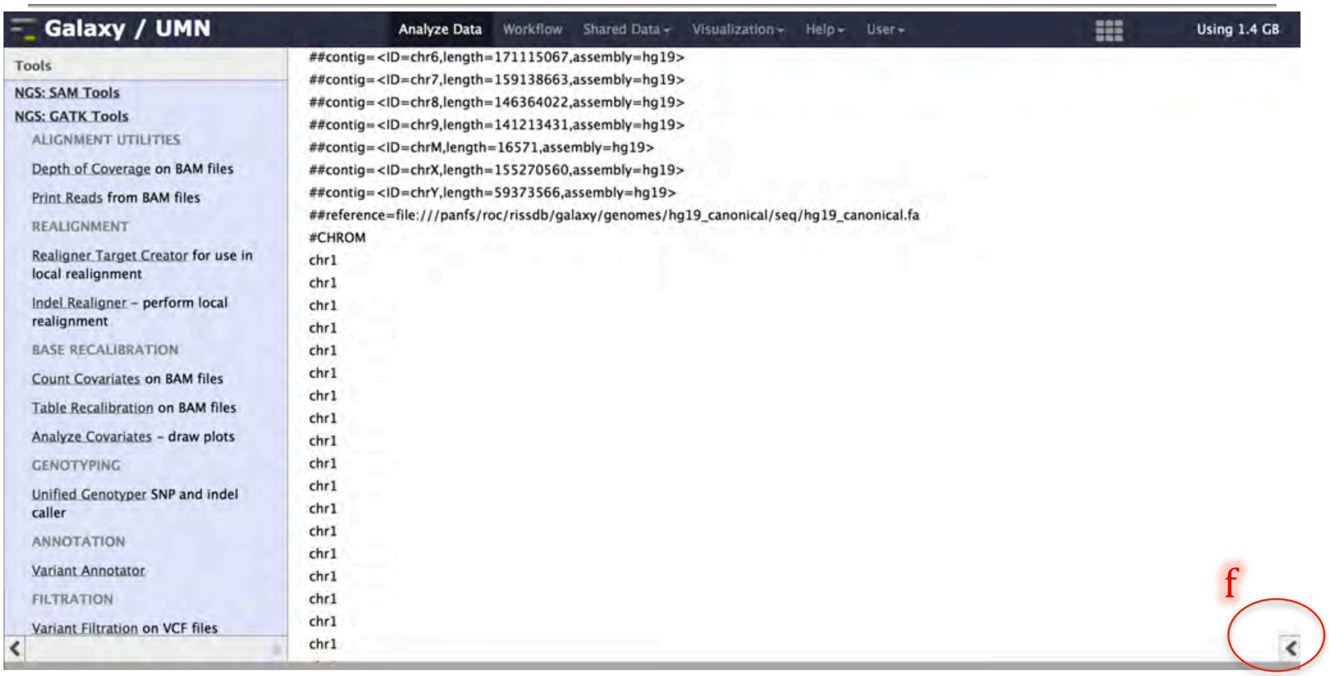

7 GATK Phase 3: Preliminary Analysis

★ **GATK Phase 3 details**

★ **Variant Recalibration**
Any pipeline that takes raw sequencing reads, maps them to a reference and attempts to make genotype calls will have inherent systematic errors leading to false-positive variant calls. *The challenge is to separate true genotype calls from machine artifacts*. The GATK's Variant Quality Score Recalibrator attempts to separate raw variant calls into different confidence levels, or tranches, based on training from "truth" data – usually variants that have been verified. Training is done using a Gaussian Mixture model. A wide variety of external evidence can be used to help train the recalibrator:

• **Known dbSNP rates**
As a result of the 1000 Genomes project, it is estimated that 99% of all variants have been cataloged for Caucasian samples. The numbers are nearly as high for some African and Asian populations. Therefore, it stands to reason that SNPs at known sites are more likely to be real. Even more confidence may be associated with carefully validated sets like the HapMap project or the 1000 Genomes OMNI-chip validation set. If a sample has a very high rate of novel variants, yet comes from a well-sampled population, the quality of those calls is circumspect.

• **Transition (Ti)/Transversion (Tv) rates are non-random**
Transition (A<->G, C<->T) and transversion (A<->C, A<->T, C<->G, G<->T) ratios are not random (0.5). Selection pressure works against transversions in coding DNA and in other structurally or functionally-relevant regions. High throughput validation studies have established that Whole genome sequencing typically yields Ti/Tv rates ~2.0-2.1, and exome data around ~3.0-3.3. Lower rates for samples are highly indicative of poor-quality calls.

• **Population-specific heterozygosity should hold**
If you are running the GATK with multiple samples, you have the opportunity to infer heterozygosity rates. Significant deviations from Hardy-Weinberg equilibrium (e.g., observing all AT calls with no AA or TT calls at a locus) often indicate a systematic problem.

• **FisherStrand (FS)**
Variants identified in regions where nearly all reads are on one strands are more likely to be artifacts of poor mapping.

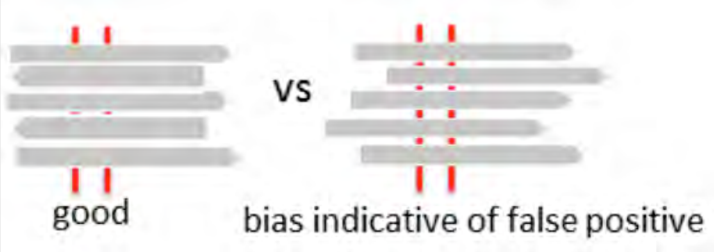

• **HomopolymerRun (HRun)**
Long tracts of single-nucleotide repeats are prone to error arising during library creation (e.g., template slippage) and mapping.

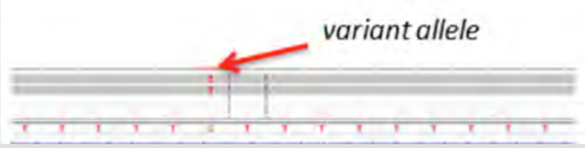

• MappingQualityRankSumTest (MQRankSum)
True heterozygous calls should have reference calls and alternate calls with comparable mapping quality. Suspicion is raised if the alternate calls preferentially appear in poorer-quality mapped reads.

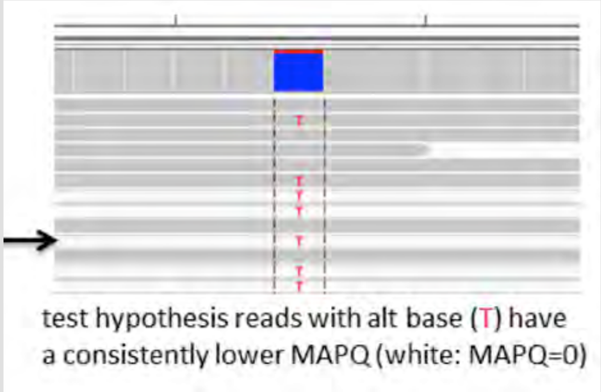

• QualByDepth (QD)
One would expect that the deeper coverage you get, the higher the confidence you should have in the variant call. But this isn't strictly correct. It is only true if the majority of the bases are of high quality and they fall in reads that are confidently mapped. Recall, the Unified Genotyper only makes genotype calls based on the "good bases" at a locus. In other words, the low quality bases or bases that occur in poorly mapped reads do not contribute to the raw genotype quality score. So, we should penalize pileups that have a high proportion of "bad bases". This is achieved here by taking the raw confidence assigned by the unified genotyper for a variant site *divided by* the *unfiltered* depth. This measure properly penalizes huge pileups with poorly mapped reads or basecalls (usually due to collapsed repeats).

• ReadPosRankSum
One would expect variant calls to be randomly distributed in position along a read, and not preferentially appear near the beginning or end of reads. Bias in the positioning of the alternate could be evidence of mismapping.

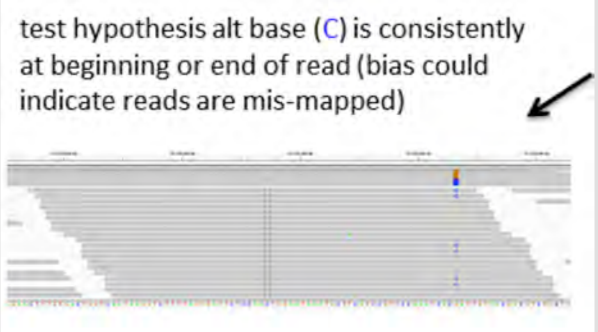

★ Variant Annotation
Once you obtain a list of variants in VCF format, it is highly desirable to know the potential effect of the variant on surrounding genes. For example, a variant might be upstream, downstream or intronic relative to specific genes, or it might be a synonymous coding SNP, non-synonymous coding or splice-site altering SNP, etc. Additionally, it may be desirable to predict whether an SNP is potentially deleterious based on the conservation level of the affected sequence, and protein 3D structure elements, etc. This information may be obtained using the widely-popular tool annovar (http://www.openbioinformatics.org/annovar/). Although this tool is free, licensing prohibits it from being wrapped and re-distributed in Galaxy. Galaxy includes an alternative tool called snpEff that is better integrated with Galaxy and the GATK, and has nearly the same level of functionality.

Variant Recalibration

7.1 Select SNPs

a) Load *Select variants* tool from the tool pane: “NGS: GATK Tools -> Select Variants from VCF files”
b) Variant file to select: -> “…Unified Genotyper….(VCF)”
c) Using reference genome: -> Homo sapiens hg19_canonical (GATK)
d) Basic or Advanced Analysis options: -> Advanced

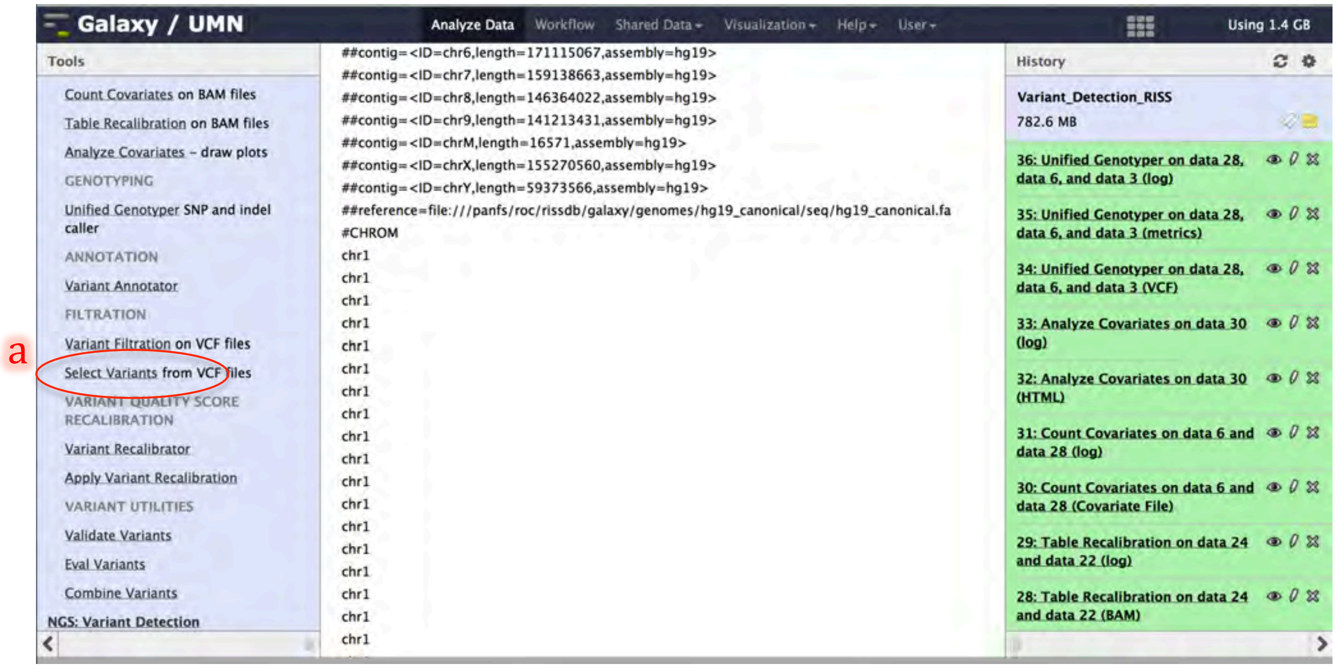

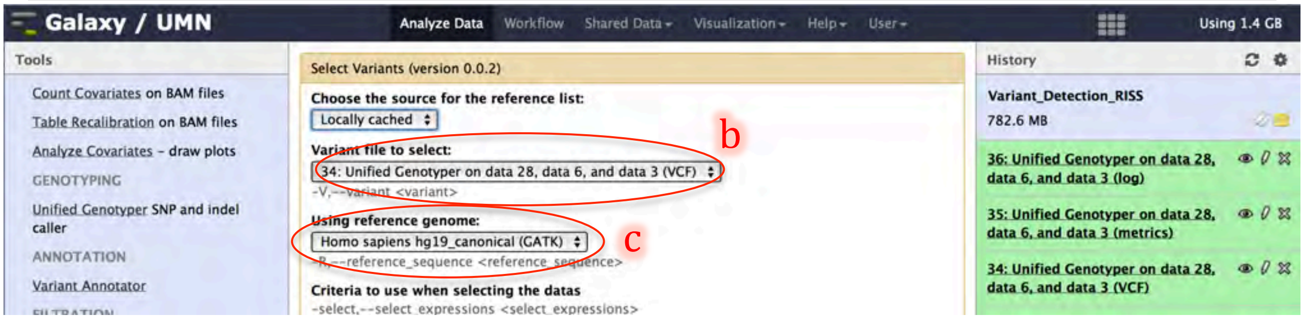

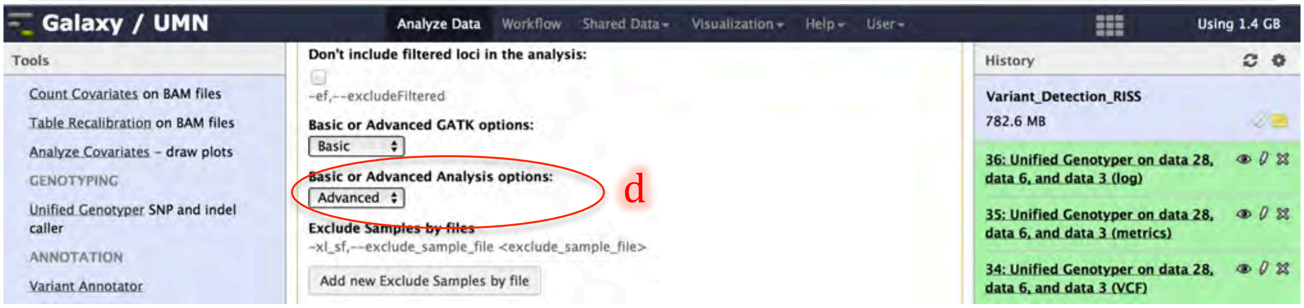

e) Select only a certain type of variants from the input file: -> check box next to
   ✓ SNP
f) Click "Execute"
g) Click the pencil icon next to the output file to edit attributes

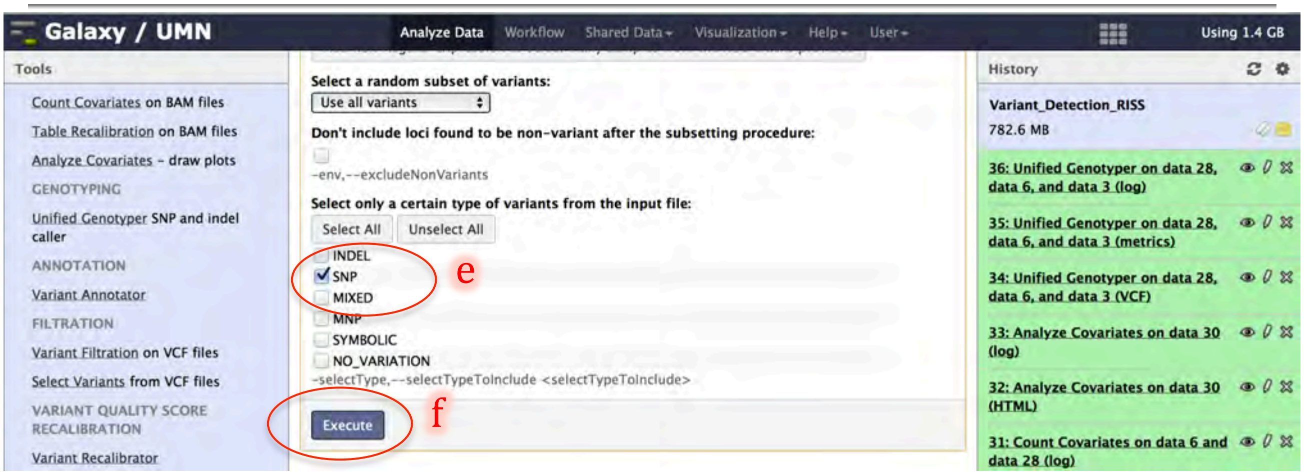

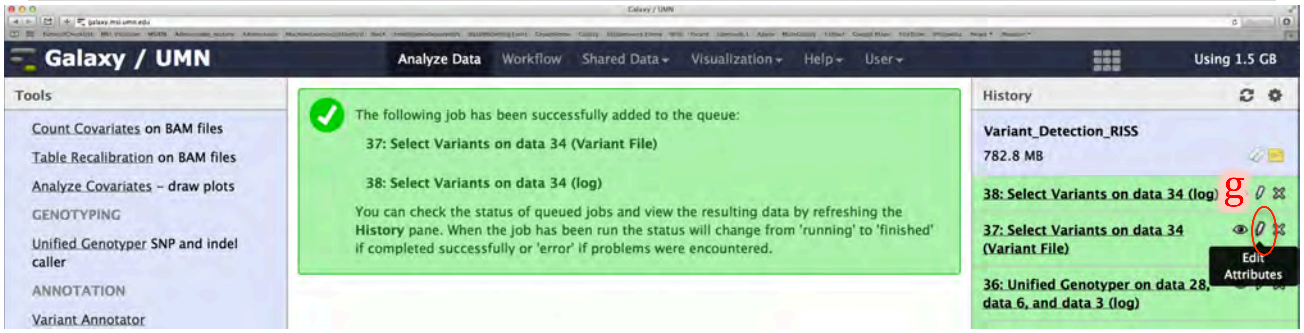

h) Enter “SNPs” under **Name:**
i) Click “Save”

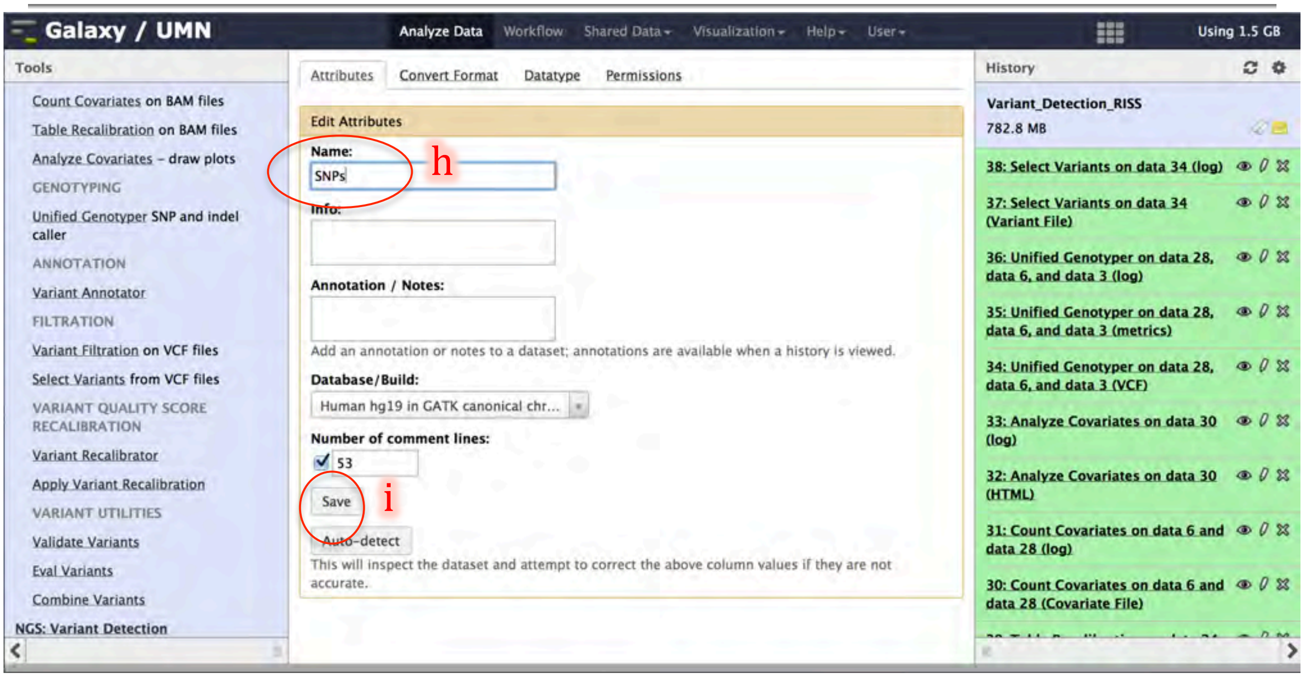

7.2 Recalibrate SNPs

a) Load *variant recalibration* tool from the tool pane: "NGS: GATK Tools -> Variant Recalibrator"
b) Variant file to recalibrate: -> "SNPs"
c) Using reference genome: -> Homo sapiens hg19_canonical (GATK)
d) Click on "Add new Binding for reference-ordered data"

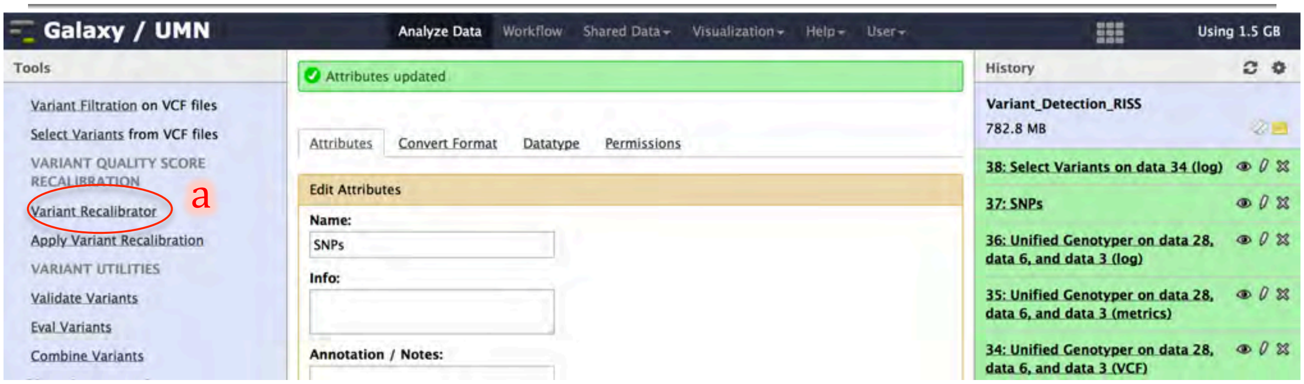

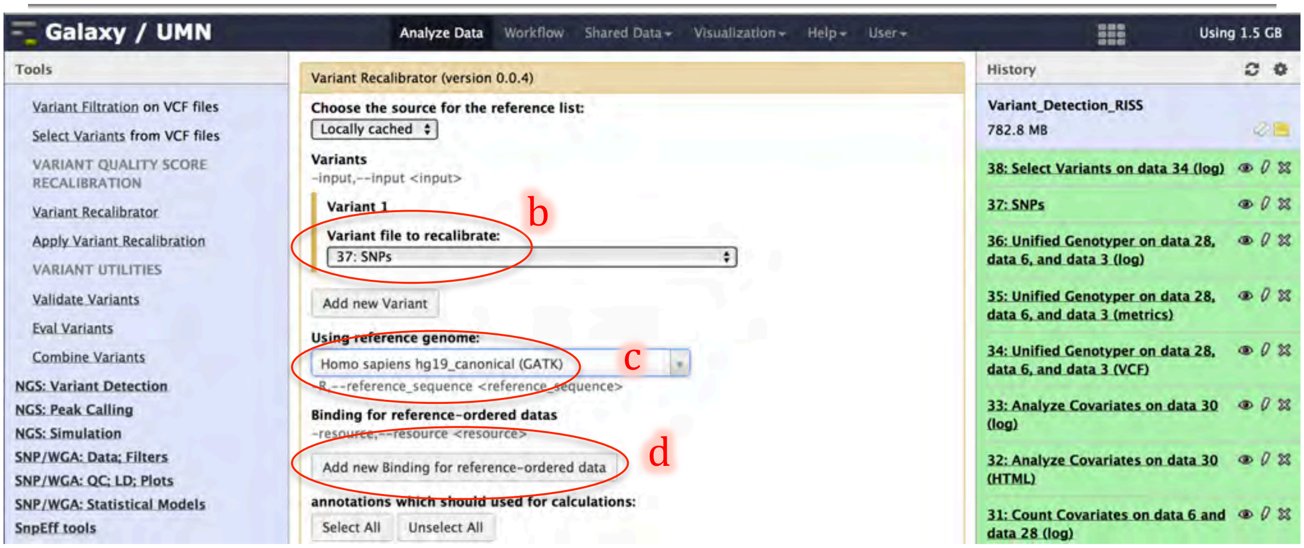

e) Binding Type: -> HapMap
f) ROD file: -> hapmap_3.3.hg19.vcf
g) Use as training/truth/known sites: -> Set training/truth/known sites
   Is Training Site: -> ✓
   Is Truth Site: -> ✓
h) prior probability of being true: -> 15.0
i) Click on "Add new Binding for reference-ordered data"
j) Binding Type: -> OMNI
k) ROD file: -> 1000G_omni2.5.hg19.vcf
l) Use as training/truth/known sites: -> Set training/truth/known sites
   Is Training Site: -> ✓
   Is Truth Site: -> ✓
m) prior probability of being true: -> 12.0
n) Click on "Add new Binding for reference-ordered data"

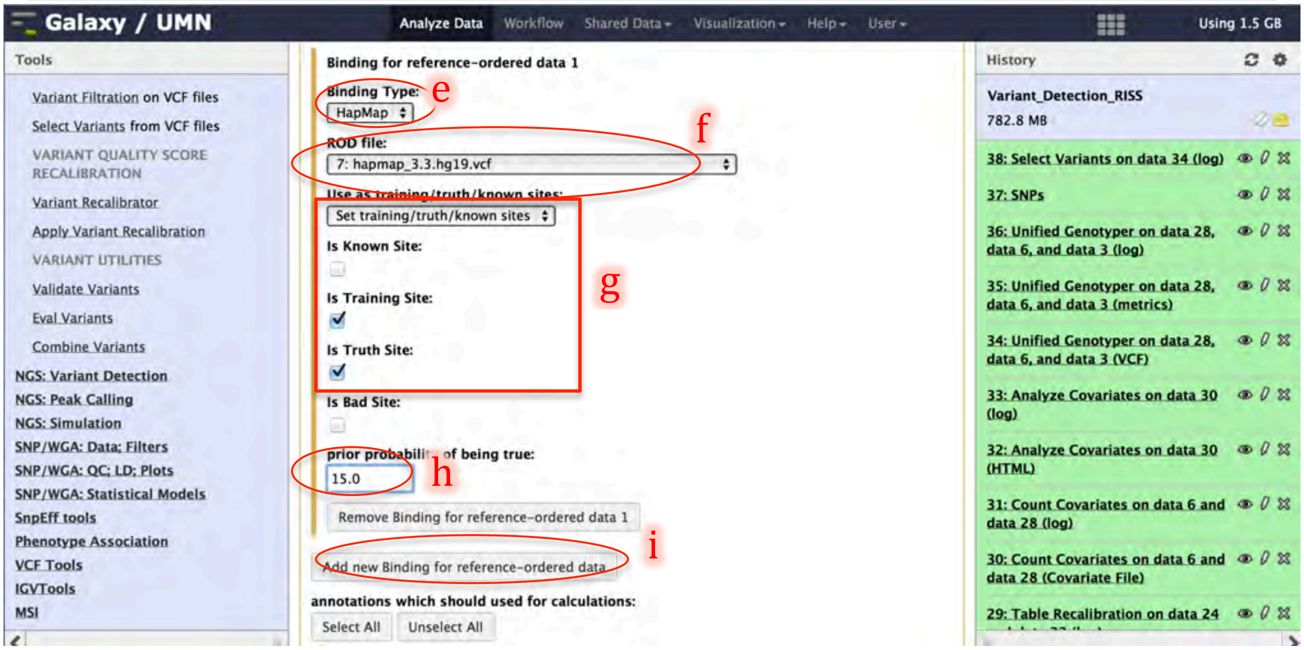

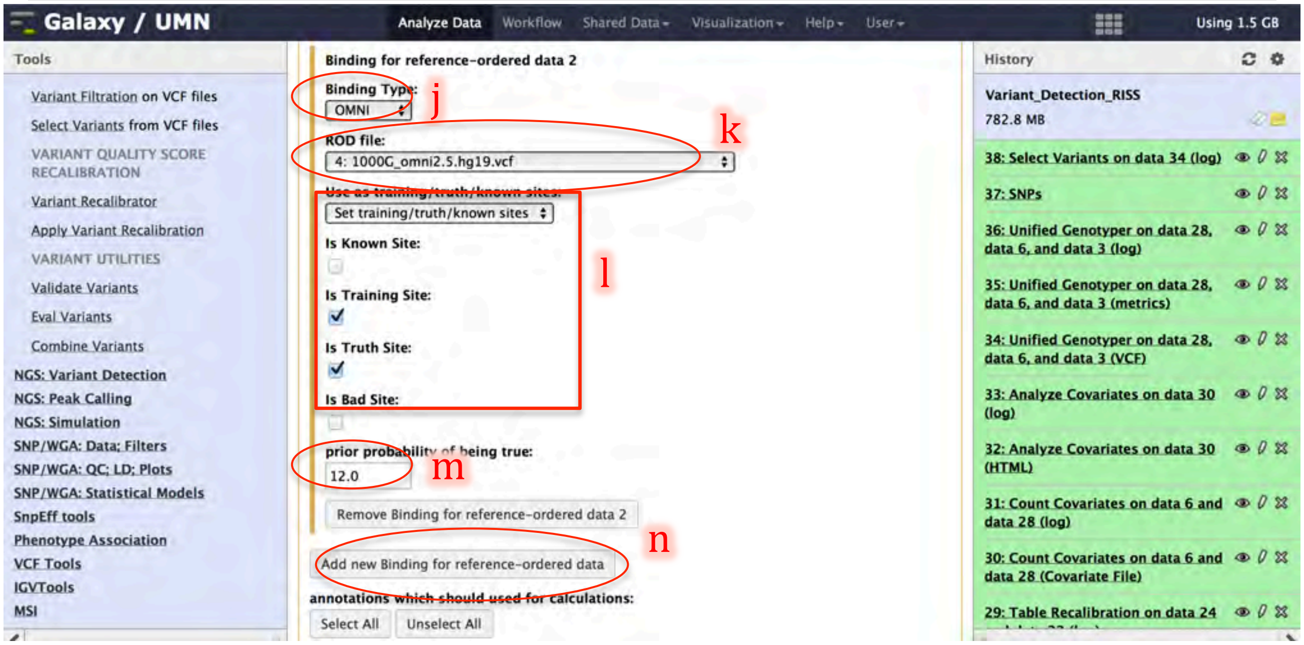

o) Binding Type: -> 1000G
p) ROD file: -> 1000G_phase1.snps.high_confidence.hg19.vcf
q) Use as training/truth/known sites: -> Set training/truth/known sites
Is Training Site: -> ✓
r) prior probability of being true: -> 10.0
s) Click on "Add new Binding for reference-ordered data"
t) Binding Type: -> dbSNP
u) ROD file: -> dbsnp_137.hg19.vcf
v) Use as training/truth/known sites: -> Set training/truth/known sites
Is Known Site:-> ✓
w) prior probability of being true: -> 2.0

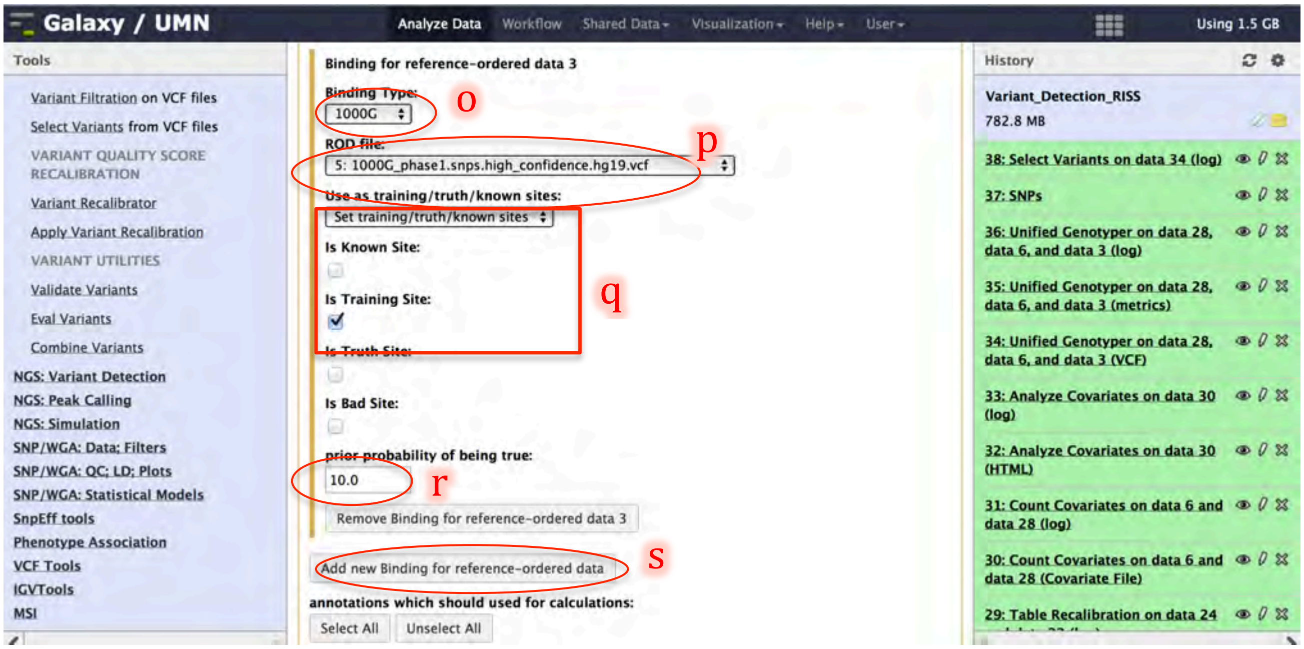

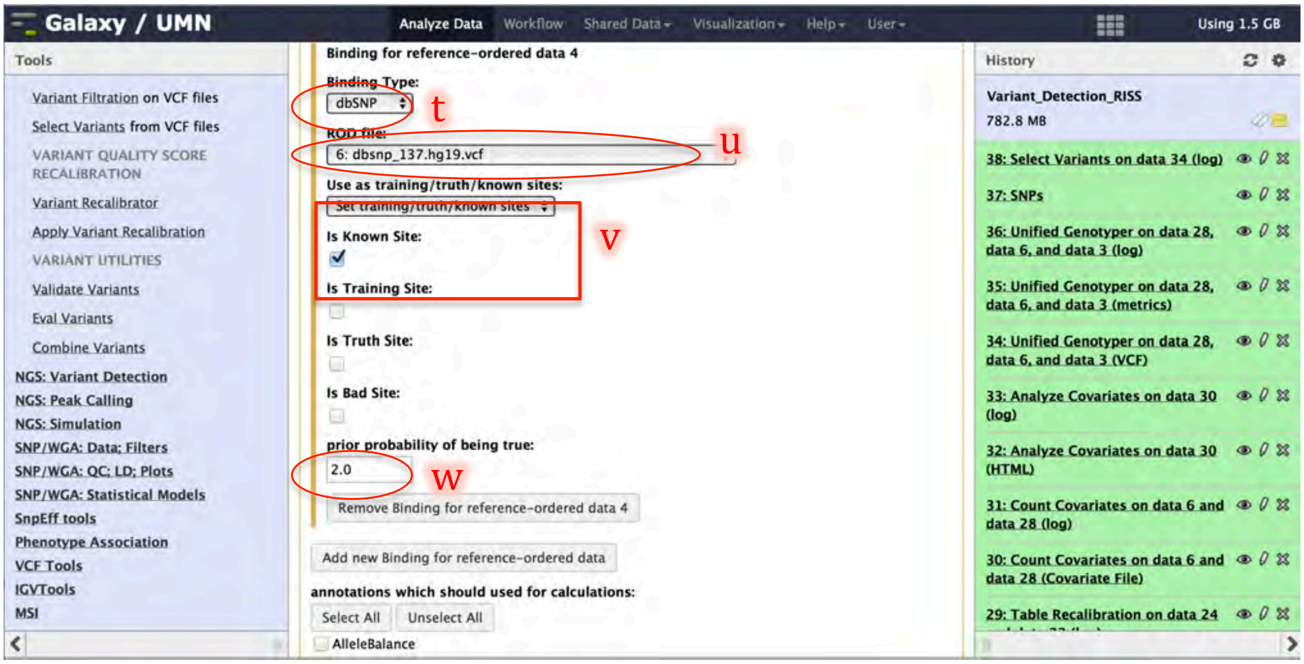

x) Click on “Add new Addition Annotations” **five times**

y) Add the annotations below

Annotation name: -> “FS”
Annotation name: -> “HRun”
Annotation name: -> “MQRankSum”
Annotation name: -> “QD”
Annotation name: -> “ReadPosRankSum”

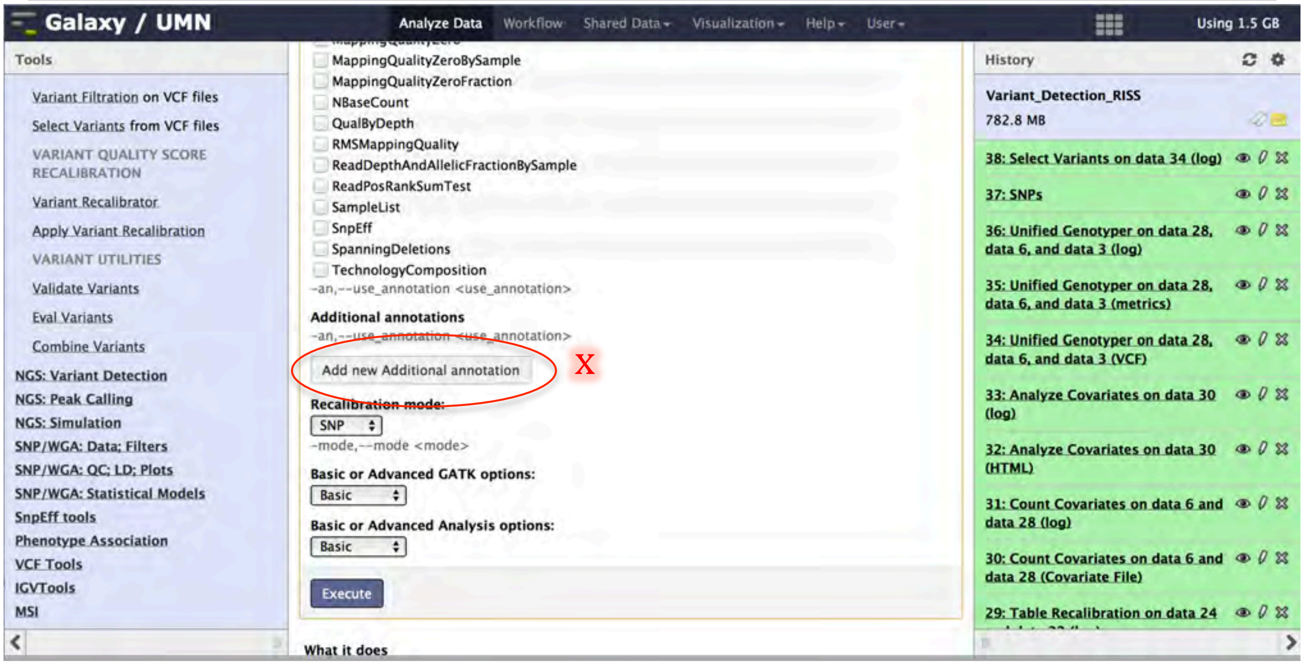

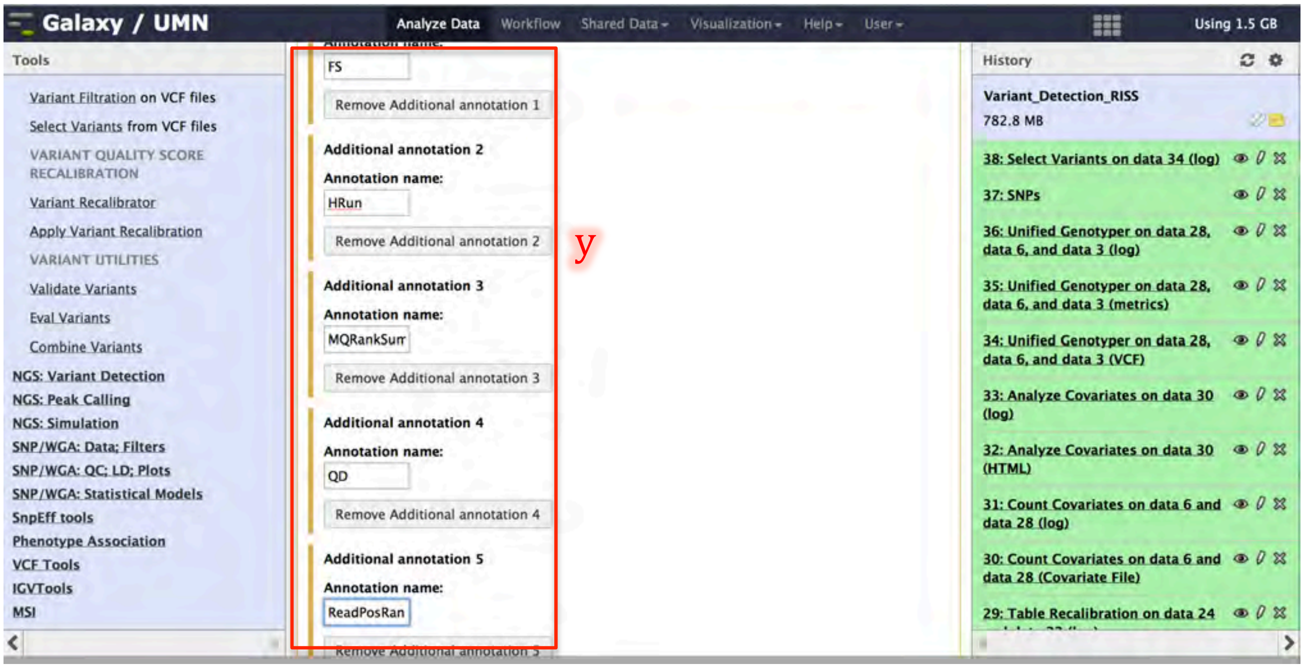

z) Recalibration mode: -> SNP
aa) Basic or Advanced GATK options: -> Advanced
bb) Click the "Add new Operate on Genomic intervals" button
cc) Genomic intervals: -> "tutorial_exons.bed"

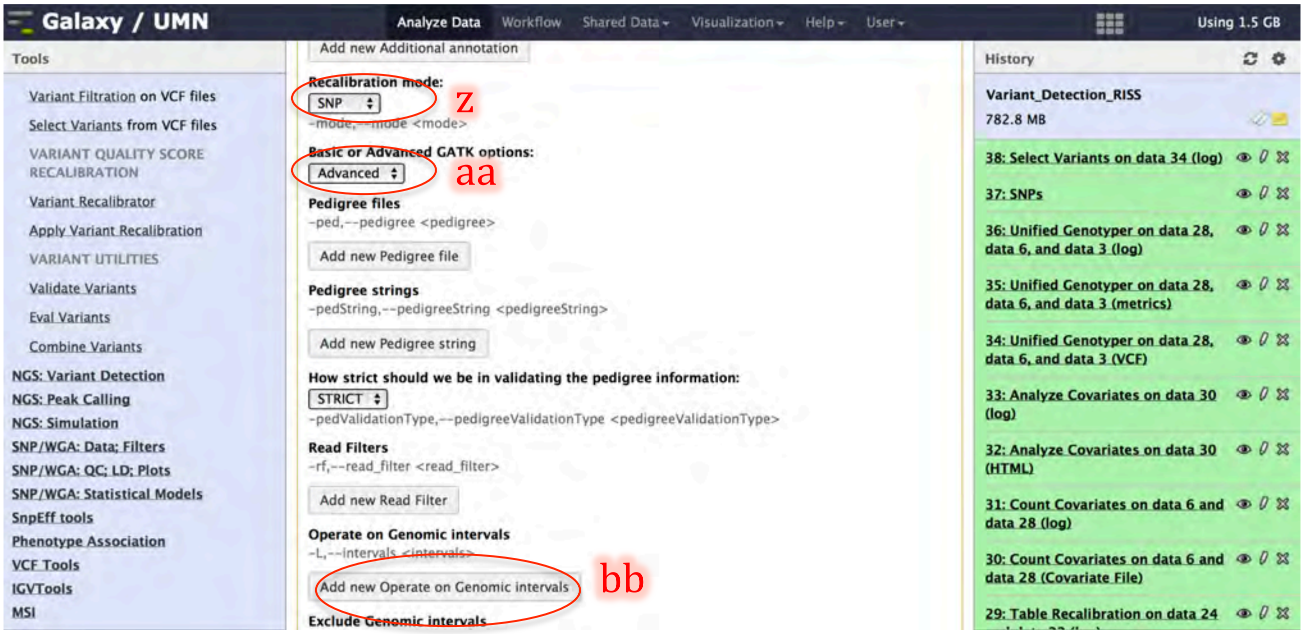

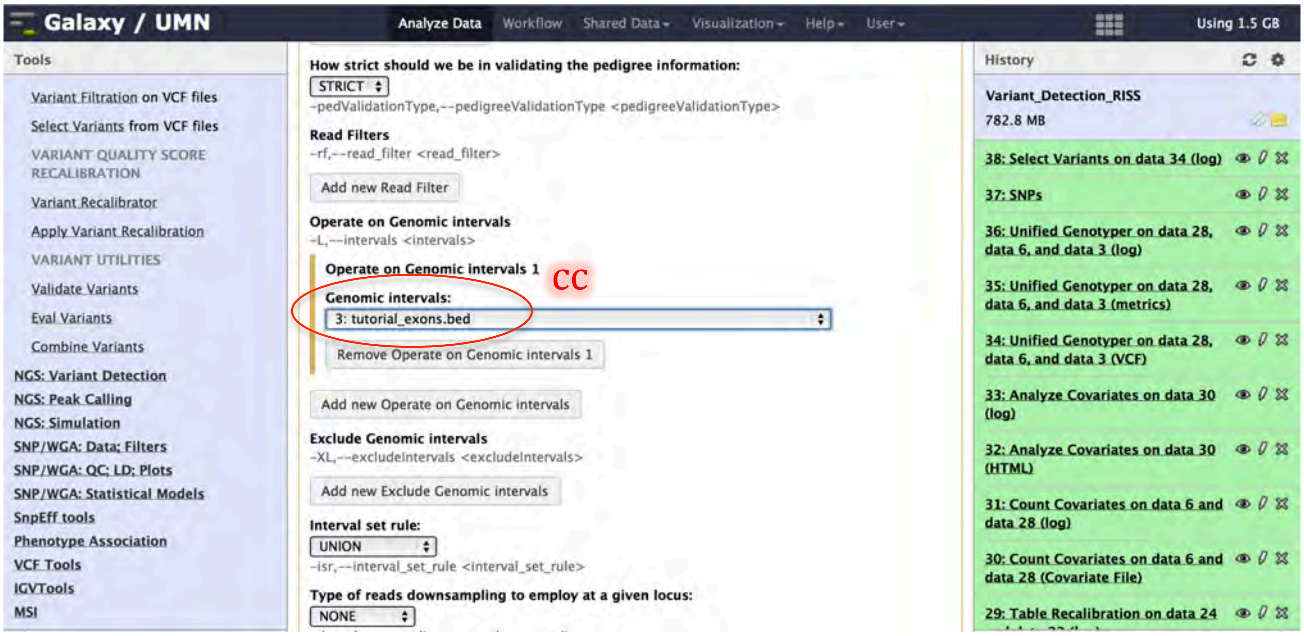

dd) Basic or Advanced Analysis options: -> Advanced
ee) maximum number of Gaussians to try during variational Bayes Algorithm <maxGaussians>: -> 1
ff) How to specify bad variants: -> Number
gg) minimum amount of worst scoring variants to use when building the Gaussian mixture model of bad variants. Will override -percentBad arugment if necessary <minNumBadVariants>: -> 50
hh) Click “Execute”

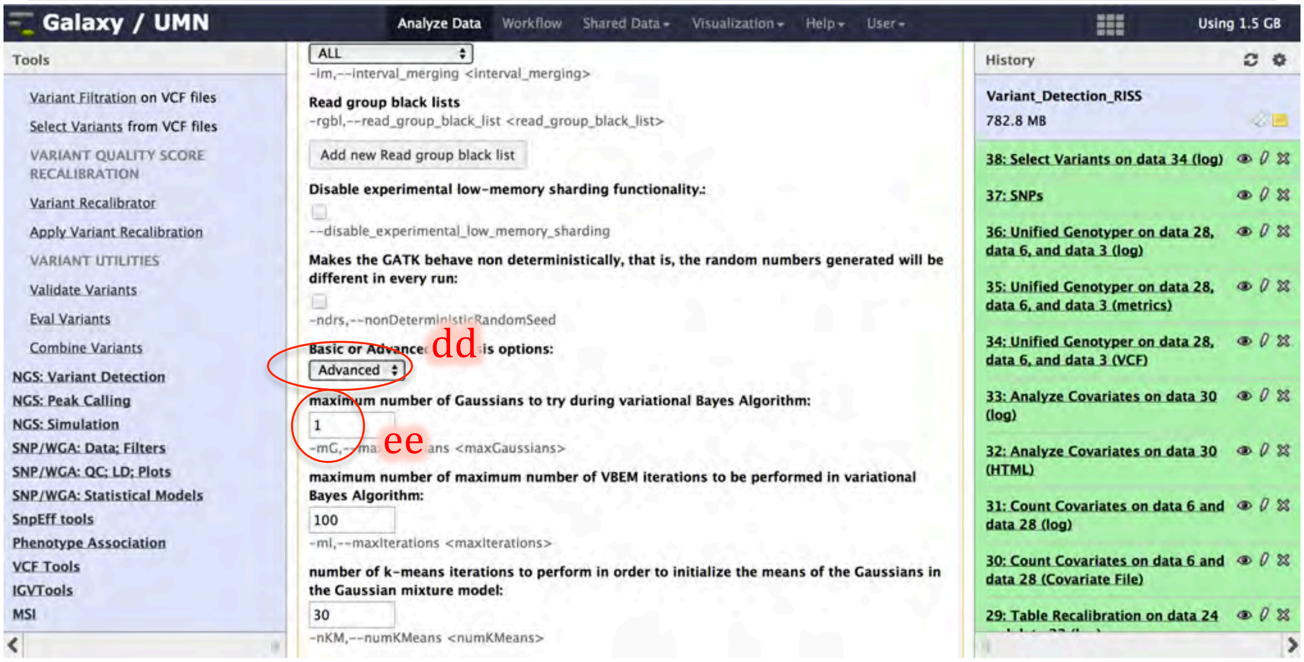

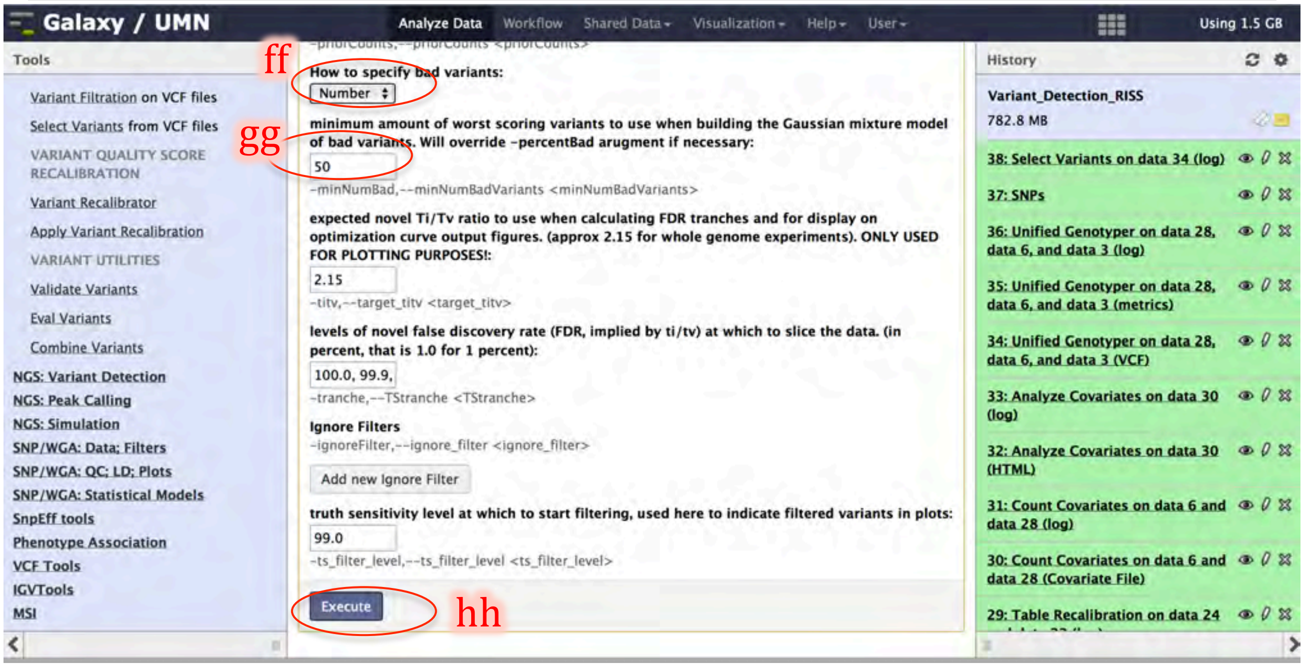

7.3 Apply recalibration

a) Load Apply Variant Recalibration tool from the tool pane: "NGS: GATK Tools -> Apply Variant Recalibration"
b) Variant file to annotate: -> "SNPs"
c) Using reference genome: -> Homo sapiens hg19_canonical (GATK)
d) Recalibration mode: -> SNP
e) Click "Execute"

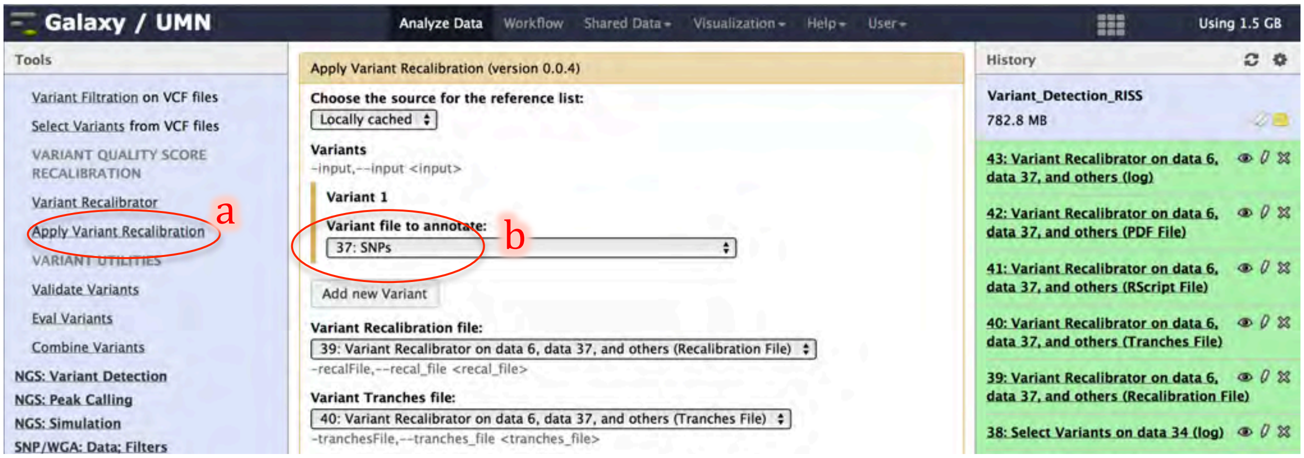

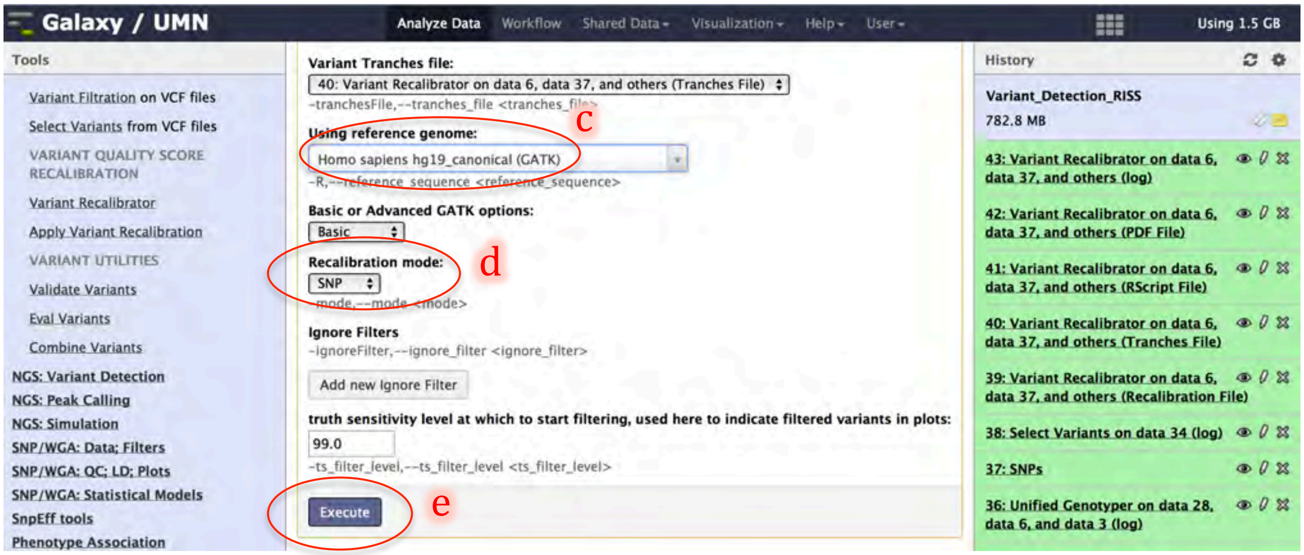

7.4 Review Variant Recalibration Models

a) In the history pane click the eye icon next to the name of the *Variant Recalibrator* pdf , “Variant Recalibrator…(PDF File)” file to display the file in the center pane
b) Move the *center pane* up and down to examine how well the models are discriminating between positive (known variants/SNPs) and negative (bad variants) training data.

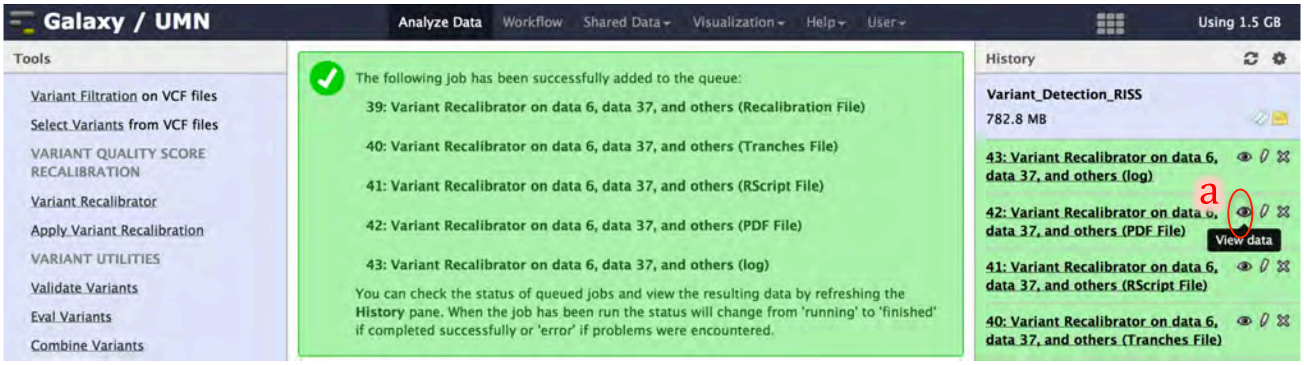

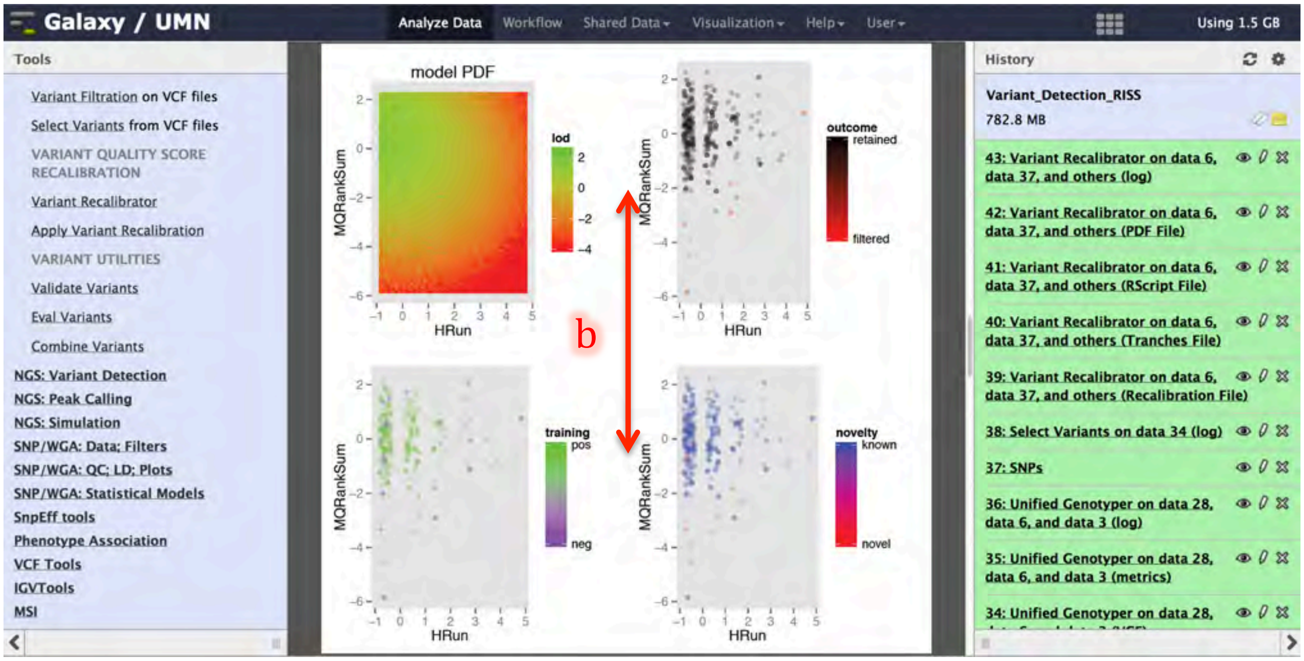

7.5 Review Recalibrated Variants (SNPs)

a) In the history pane click the eye icon next to the variant file, "*Apply Variant ….. (Variant File)*", produced by the "NGS: GATK Tools -> Apply Variant Recalibration" tool
b) Click the arrow at the bottom of the *tools pane* to the left of the browser minimize it

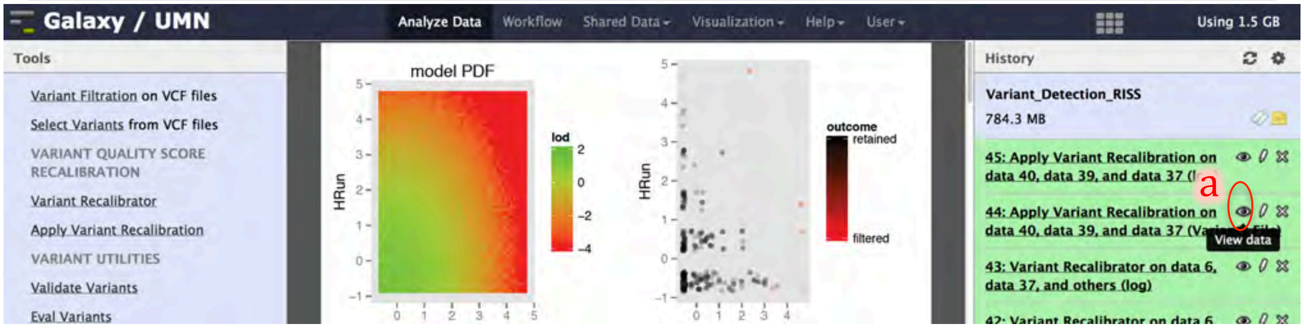

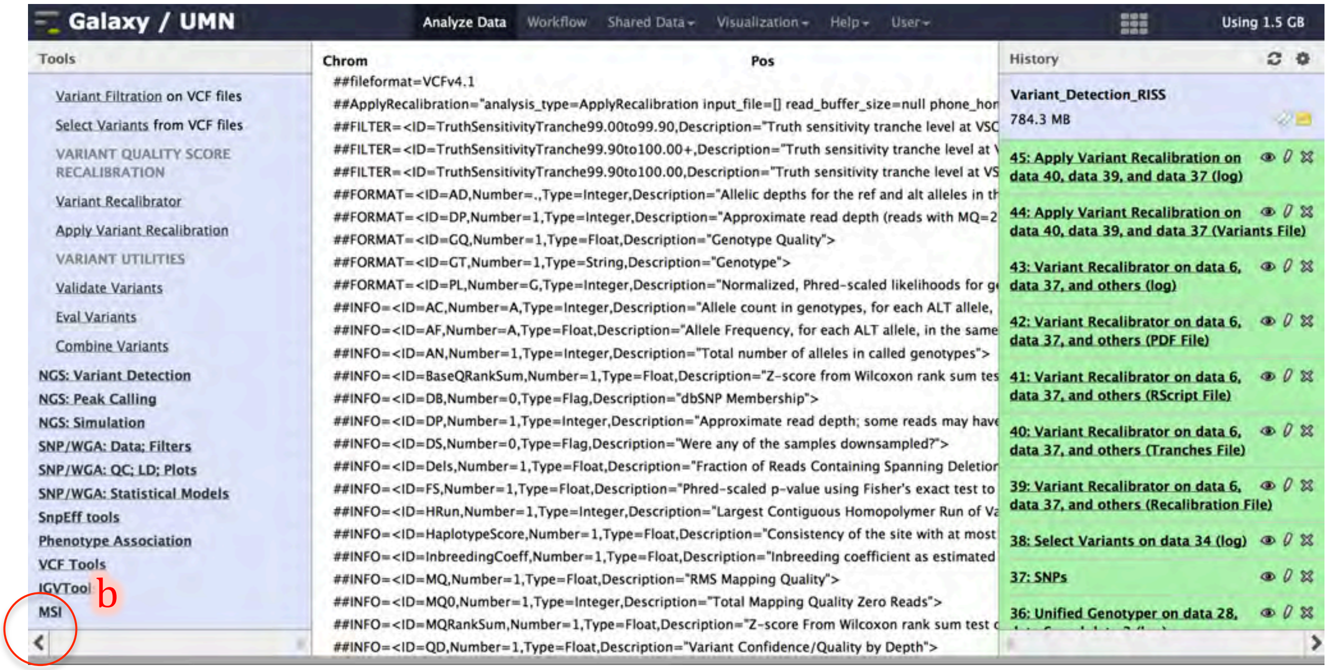

c) Click the arrow at the bottom of the *history pane* to the right of the browser minimize it
d) Scroll to the right and look at the column labeled "FILTER". Compare raw variant file produced by the Genotyper
e) Click the arrow at the bottom-left corner of your browser to bring the *tools pane* back to view

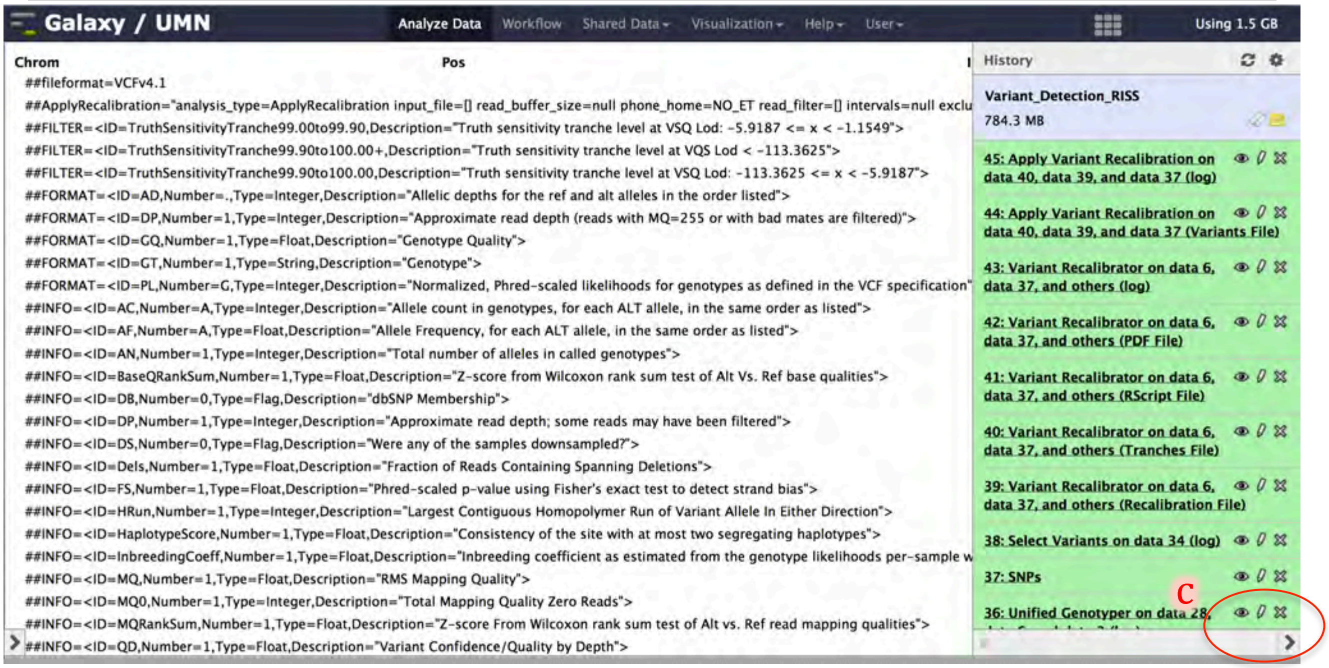

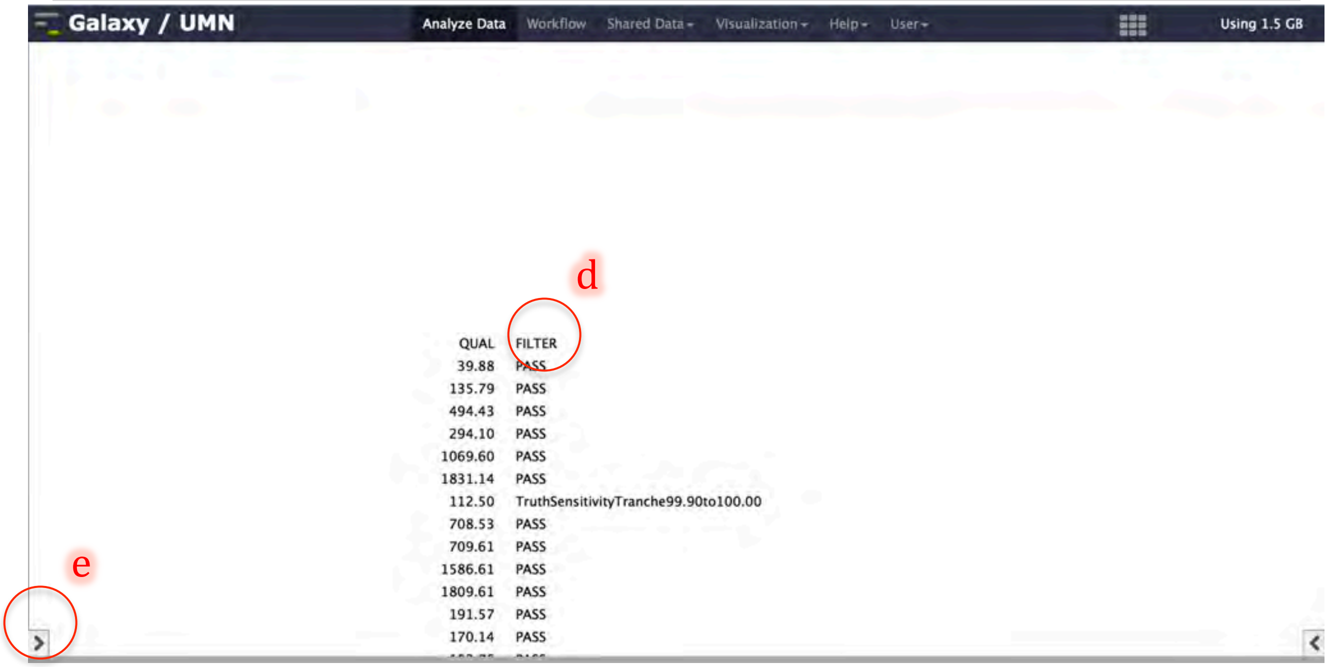

f) Click the arrow at the bottom-right corner of your browser to bring the *tools pane* back to view

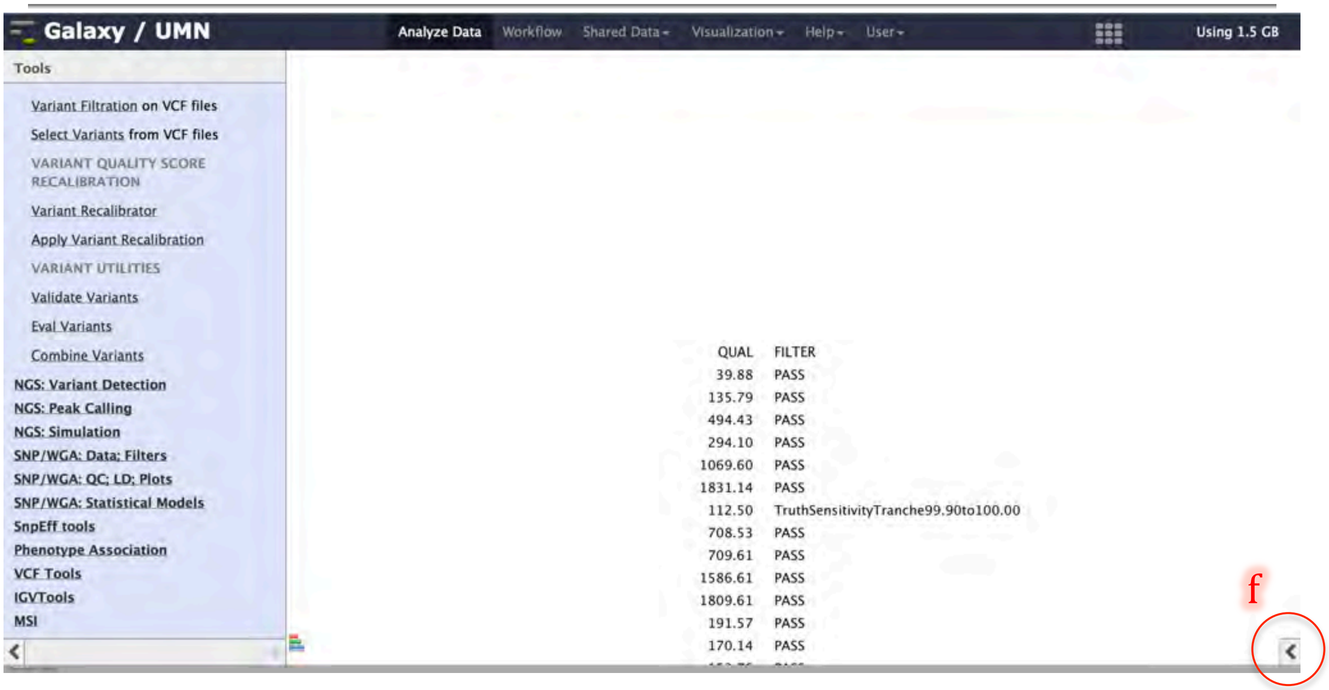

7.6 Select INDELs

a) Load *Select variants* tool from the tool pane: “NGS: GATK Tools -> Select Variants from VCF files”
b) Variant file to select: -> “...Unified Genotyper....(VCF)”
c) Using reference genome: -> Homo sapiens hg19_canonical (GATK)
d) Basic or Advanced Analysis options: -> Advanced

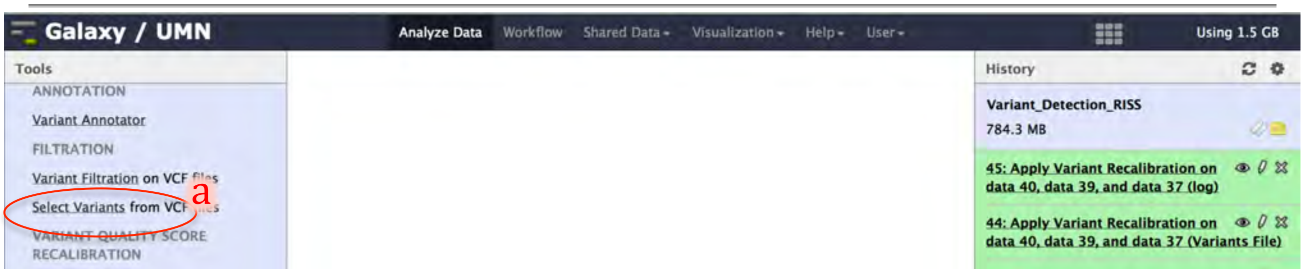

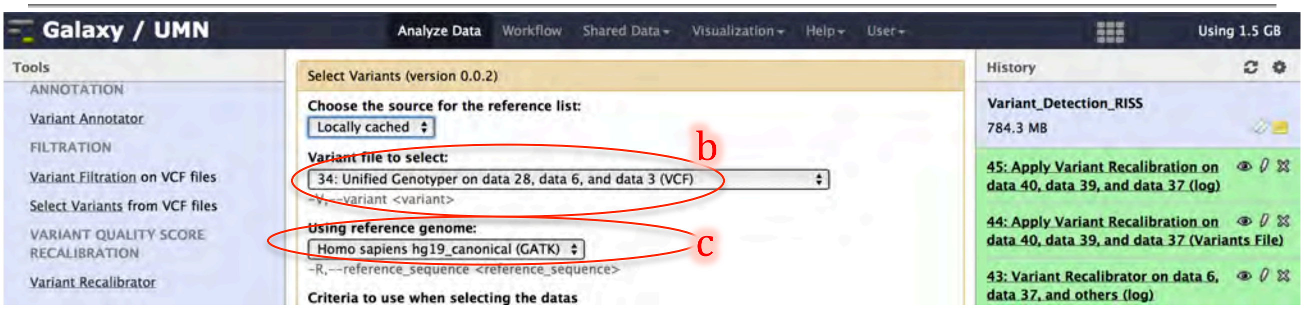

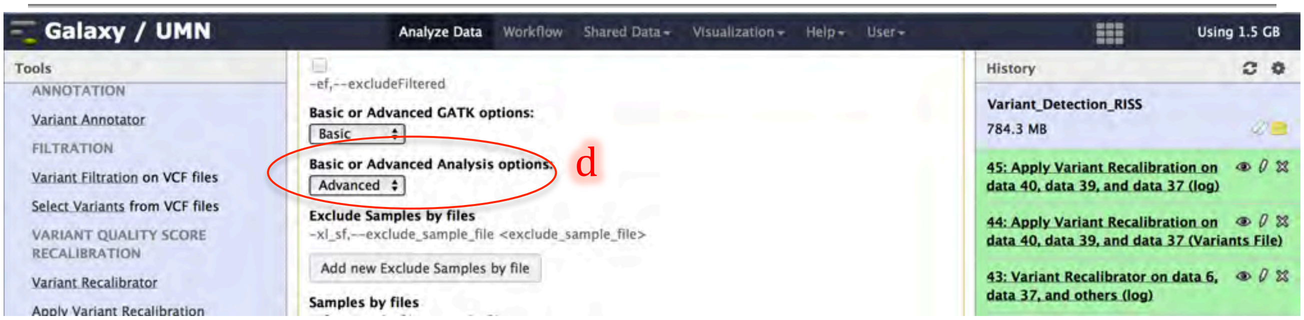

e) Select only a certain type of variants from the input file: -> check box next to
   - ✓ INDEL

f) Click "Execute"

g) Click the pencil icon next to the output file to edit attributes

h) Enter "INDELs" under **Name:**

i) Click "Save"

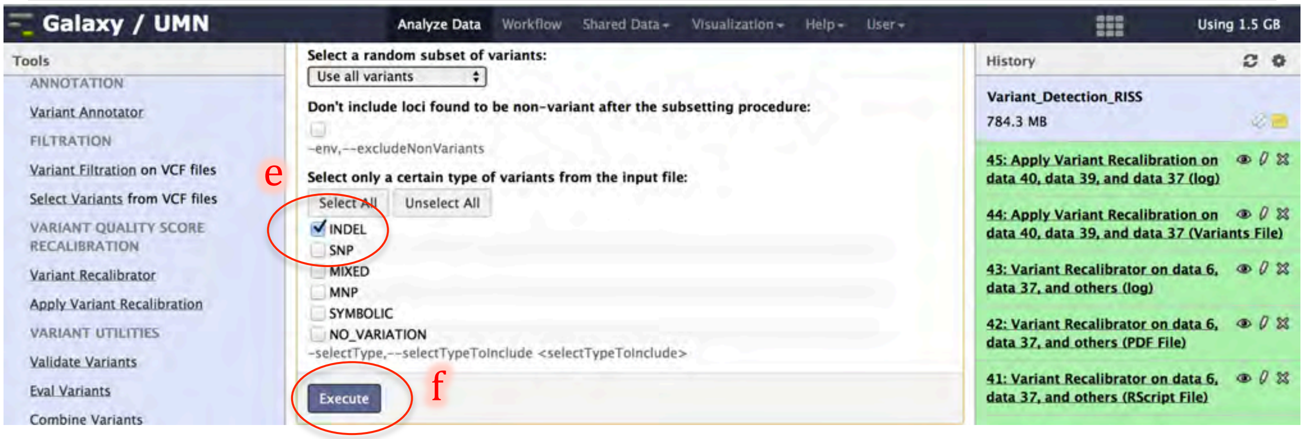

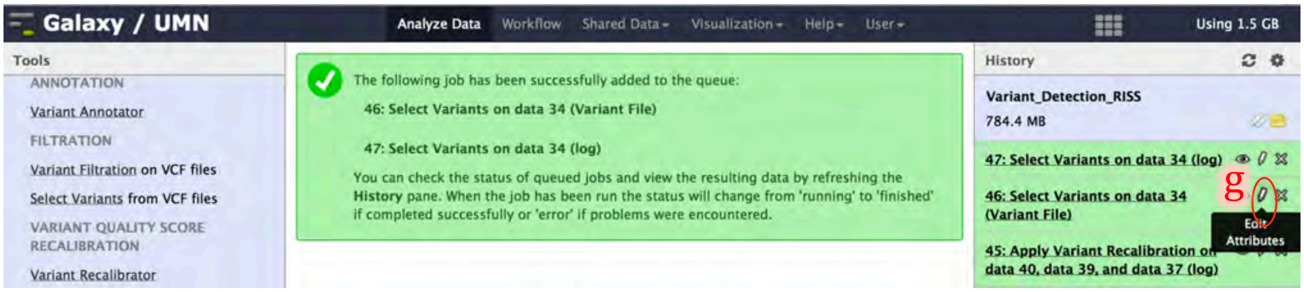

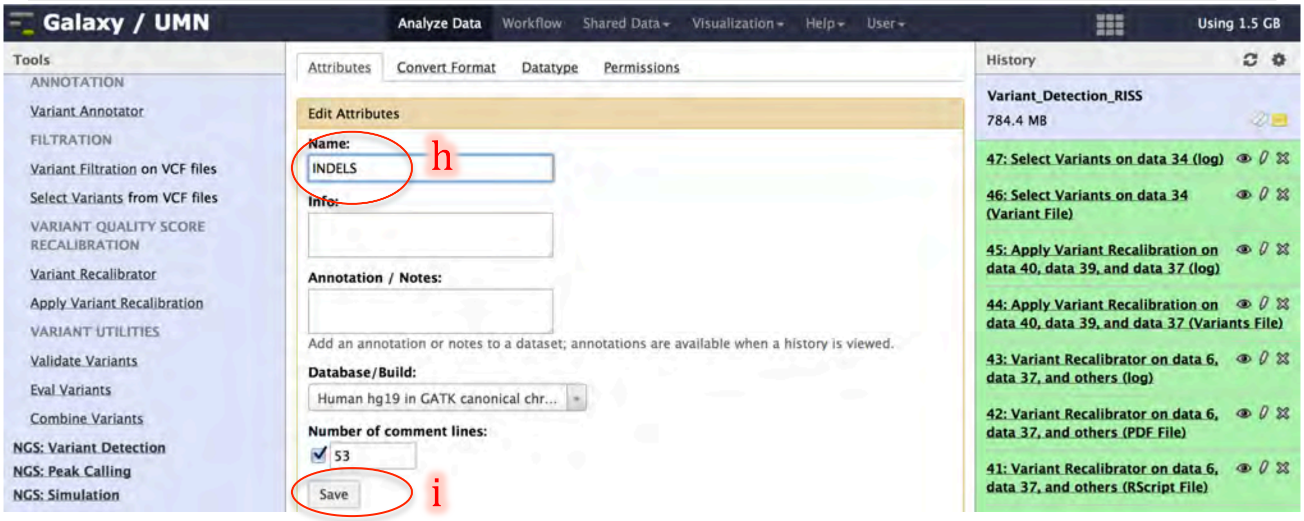

7.7 Recalibrate INDELs

a) Load *variant recalibration* tool from the tool pane: “NGS: GATK Tools -> Variant Recalibrator”
b) Variant file to recalibrate: -> “....INDELs”
c) Using reference genome: -> Homo sapiens hg19_canonical (GATK)
d) Click on “Add new Binding for reference-ordered data”
e) Binding Type: -> Custom
f) ROD Name: -> mills
g) ROD file: -> Mills_and_1000G_gold_standard.indels.hg19.vcf
h) Use as training/truth/known sites: -> Set training/truth/known sites
    Is Training Site: -> ✓
    Is Truth Site: -> ✓
i) prior probability of being true: -> 12.0
j) Click on “Add new Binding for reference-ordered data”

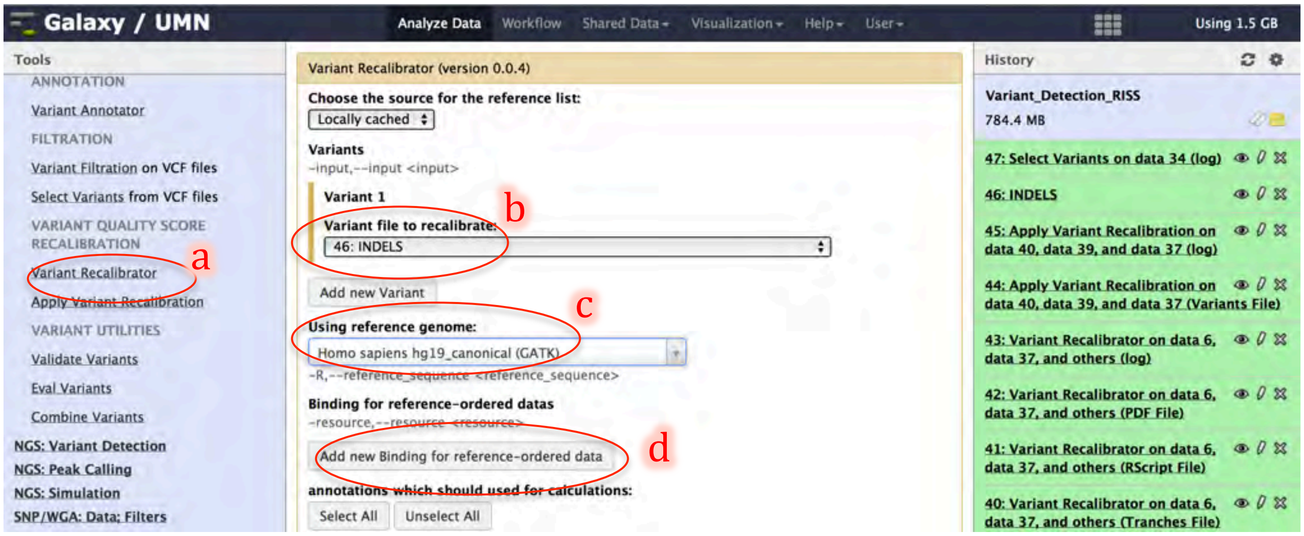

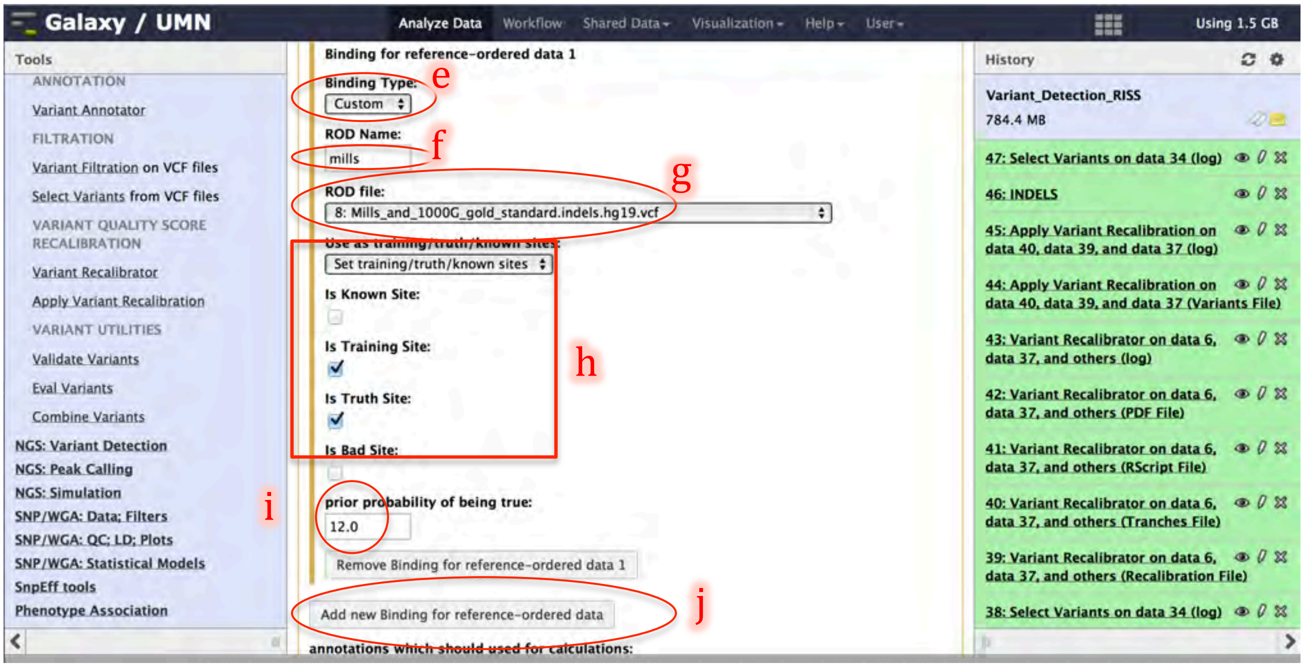

k) Binding Type: -> dbSNP
l) ROD file: -> dbsnp_137.hg19.vcf
m) Use as training/truth/known sites: -> Set training/truth/known sites
Is Known Site:-> ✓
n) prior probability of being true: -> 2.0
o) Click on "Add new Addition Annotations" **three times**

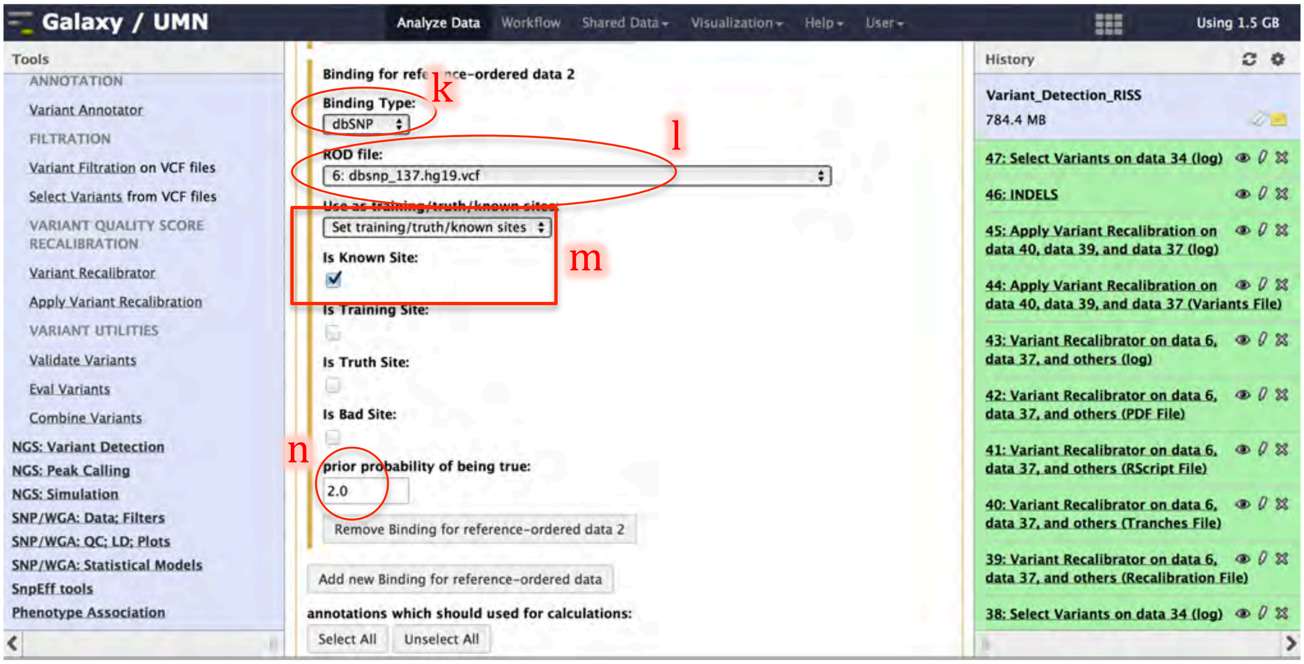

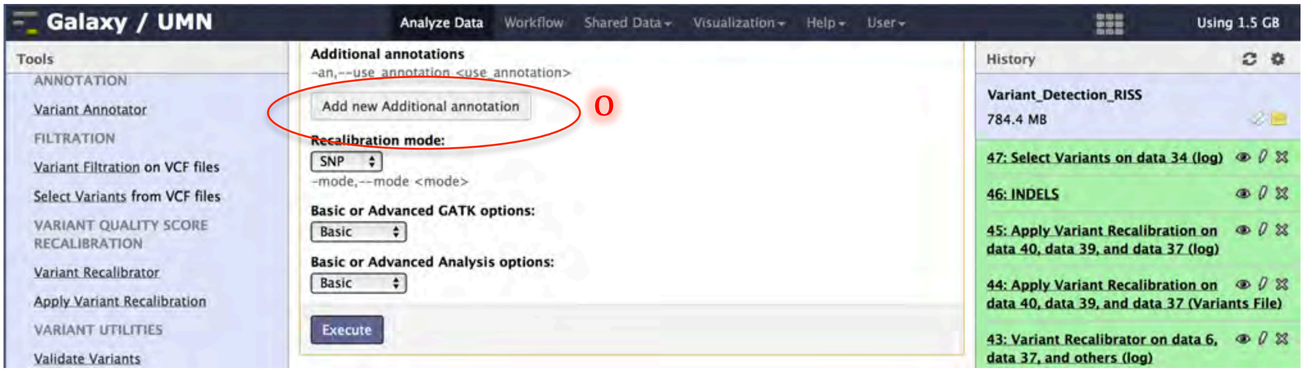

p) Add the annotations below
Annotation name: -> "FS"
Annotation name: -> "MQRankSum"
Annotation name: -> "ReadPosRankSum"
q) Recalibration mode: -> INDEL
r) Basic or Advanced GATK options: -> Advanced
s) Click the "Add new Operate on Genomic intervals" button

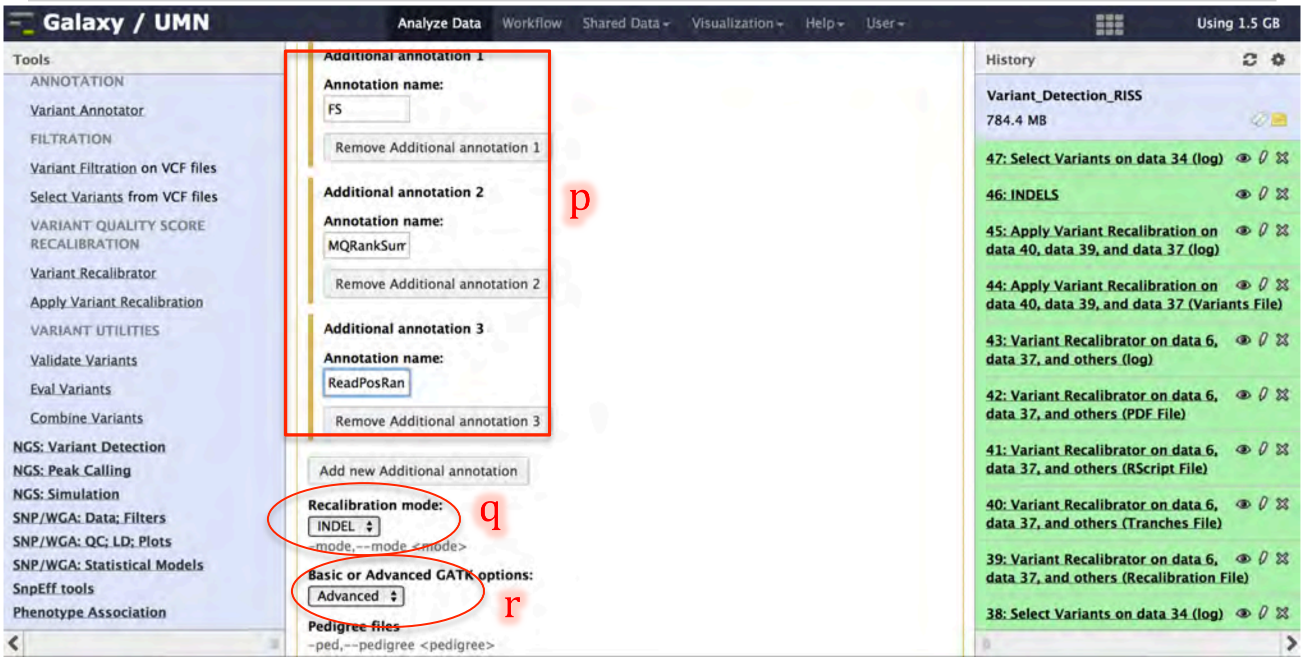

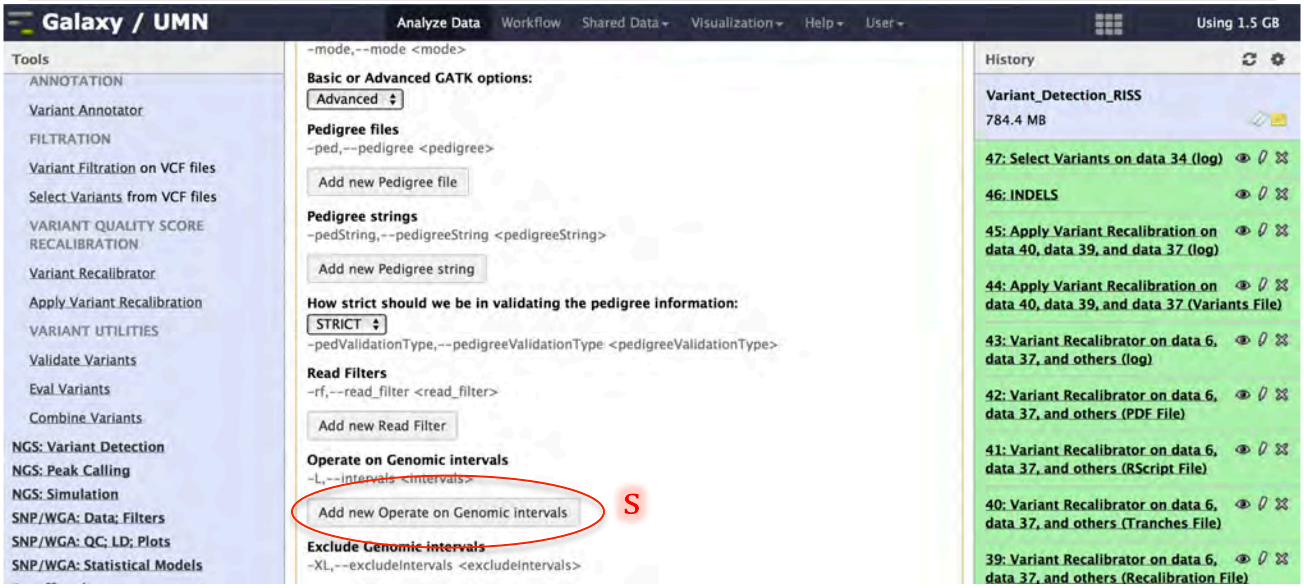

t) Genomic intervals: -> "tutorial_exons.bed"
u) Basic or Advanced Analysis options: -> Advanced
v) maximum number of Gaussians to try during variational Bayes Algorithm <maxGaussians>: -> 1
w) How to specify bad variants: -> Number
x) minimum amount of worst scoring variants to use when building the Gaussian mixture model of bad variants. Will override -percentBad arugment if necessary <minNumBadVariants>: -> 5
y) Click "Execute"

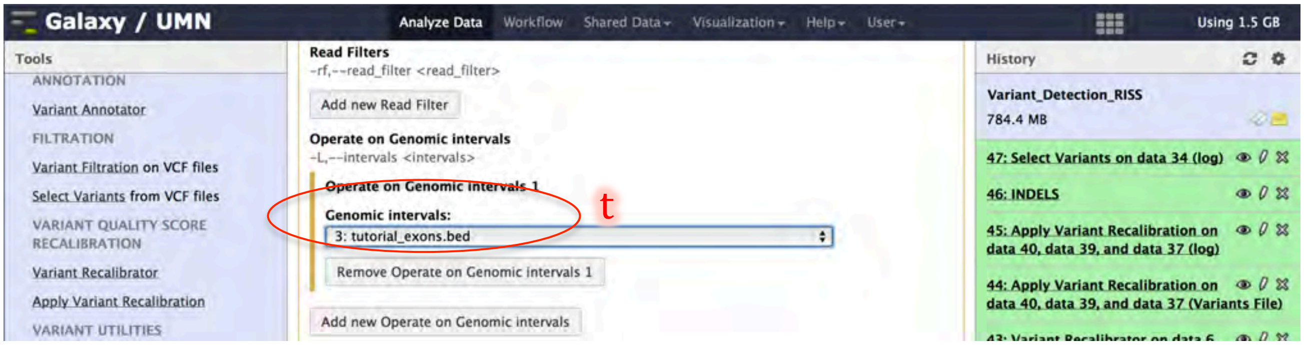

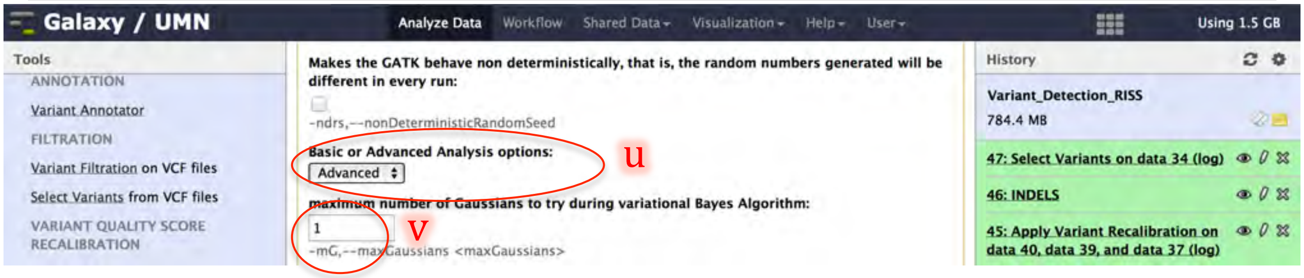

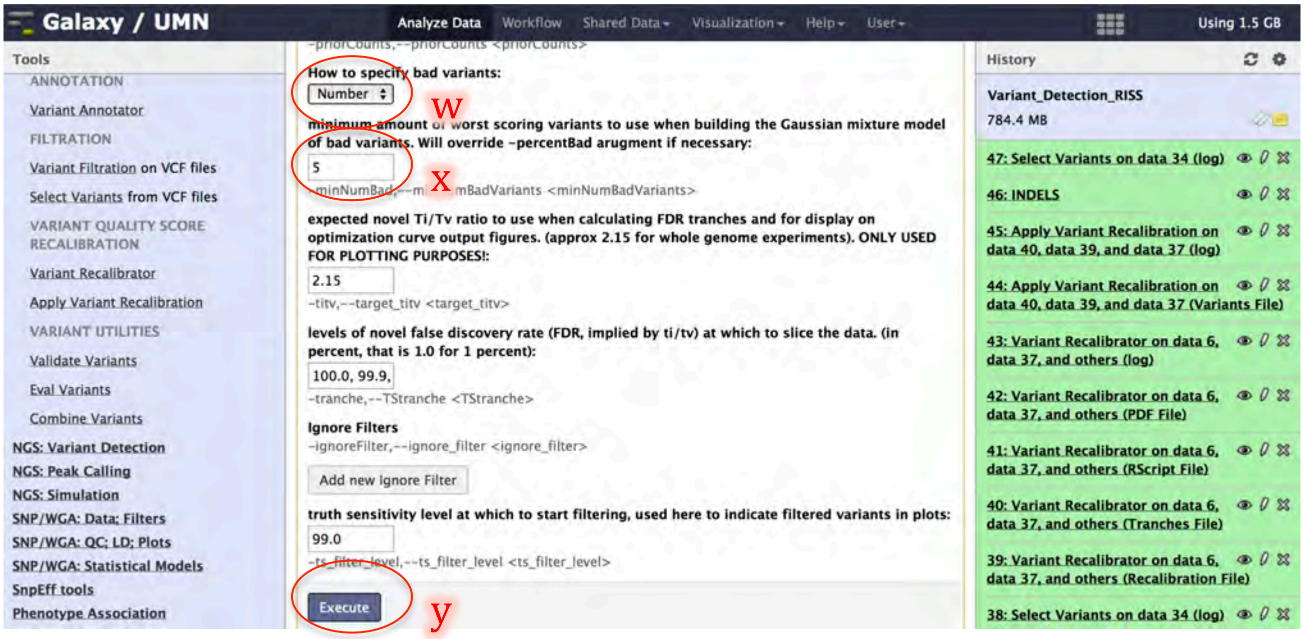

7.8 Apply recalibration

a) Load Apply Variant Recalibration tool from the tool pane: "NGS: GATK Tools -> Apply Variant Recalibration"
b) Variant file to annotate: -> "INDELs"
c) Using reference genome: -> Homo sapiens hg19_canonical (GATK)
d) Recalibration mode: -> INDEL
e) Click "Execute"

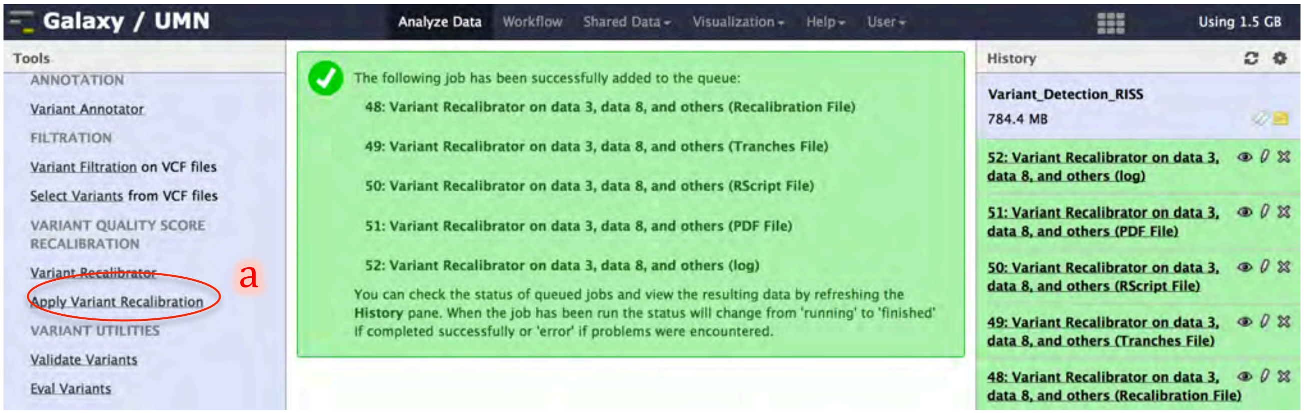

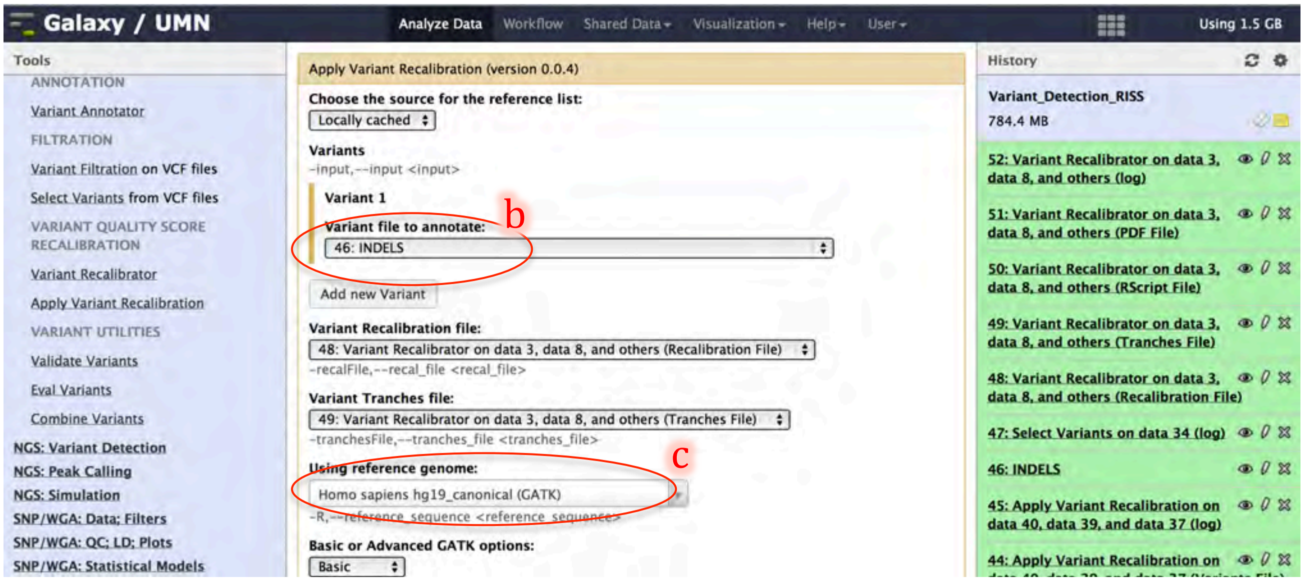

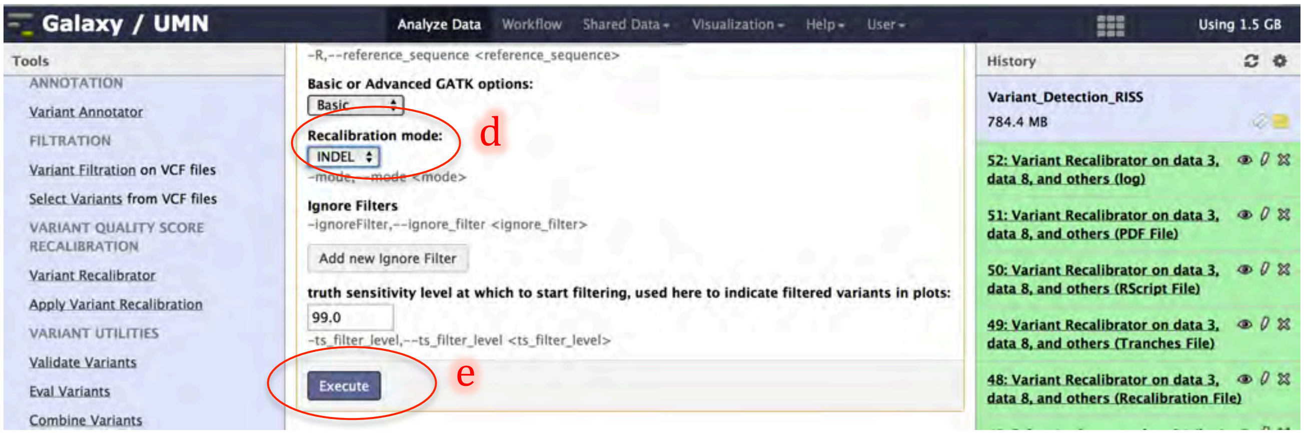

7.9 Review Recalibrated Variants (INDELs)

a) In the history pane click the eye icon next to the variant file, "*Variant Filtration ..... (Variant File)"*, produced by the "NGS: GATK Tools -> Variant Filtration on VCF files" tool
b) Click the arrow at the bottom of the *tools pane* to the left of the browser minimize it

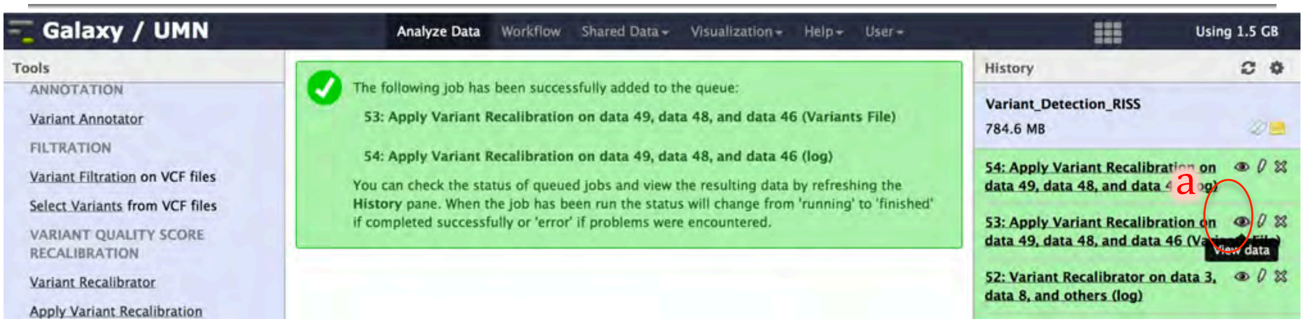

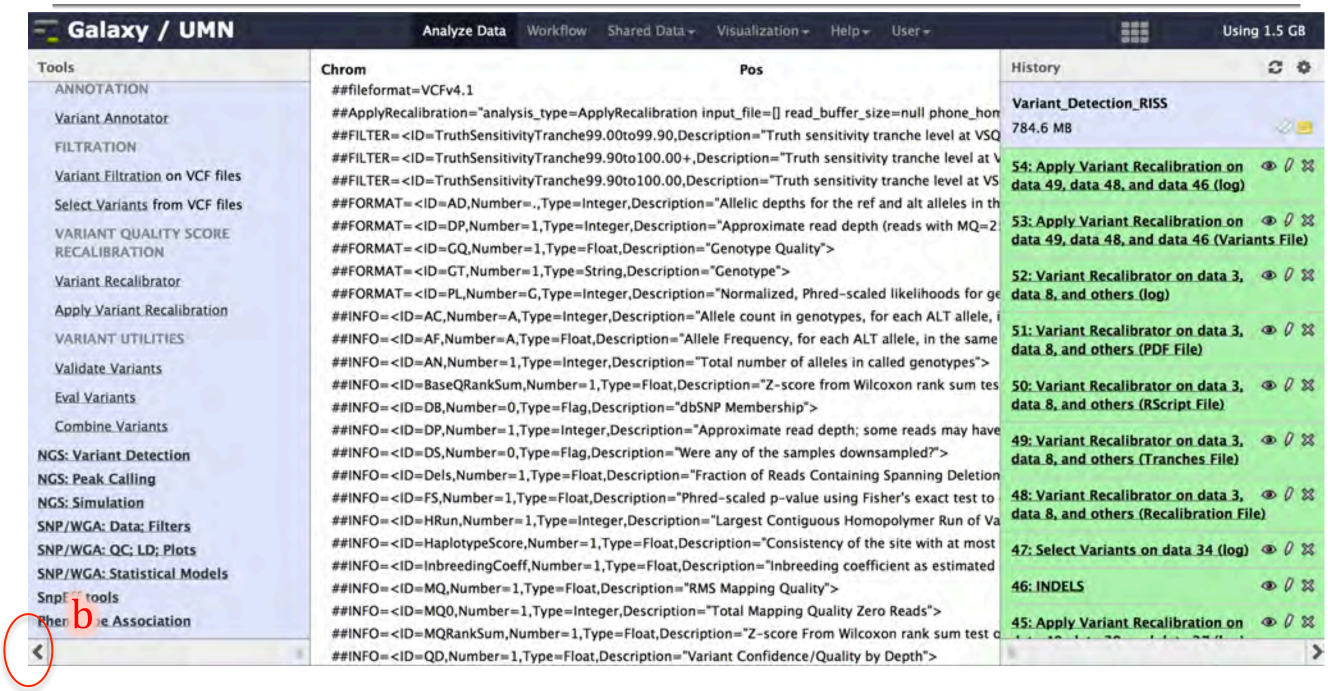

c) Click the arrow at the bottom of the *history pane* to the right of the browser minimize it
d) Scroll to the left and right to inspect recalibrated INDELs
e) Click the arrow at the bottom-left corner of your browser to bring the *tools pane* back to view

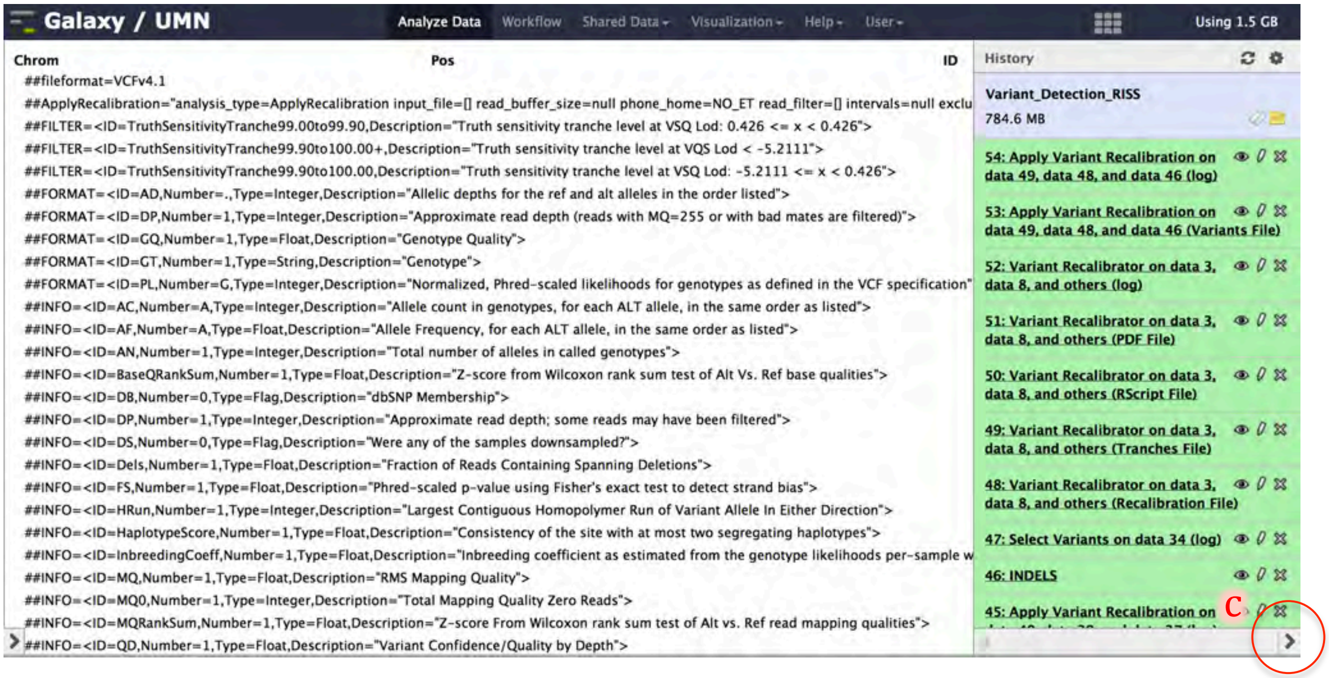

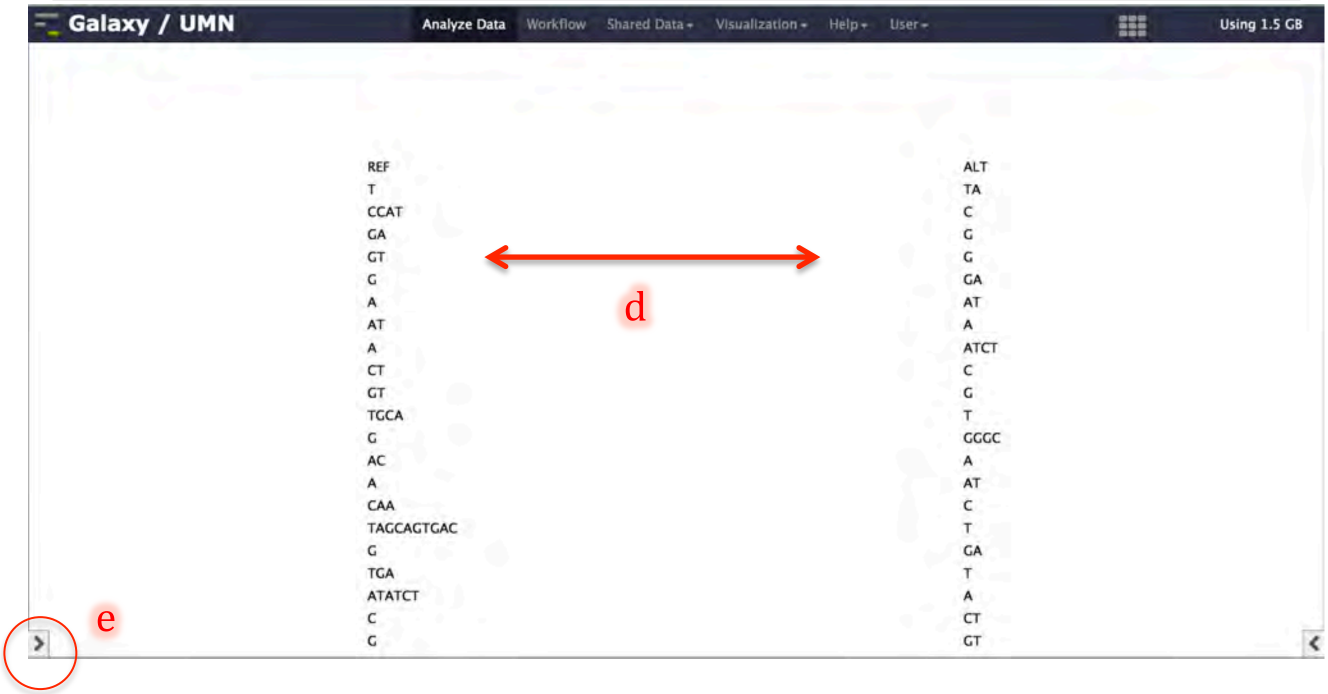

f) Click the arrow at the bottom-right corner of your browser to bring the *tools pane* back to view

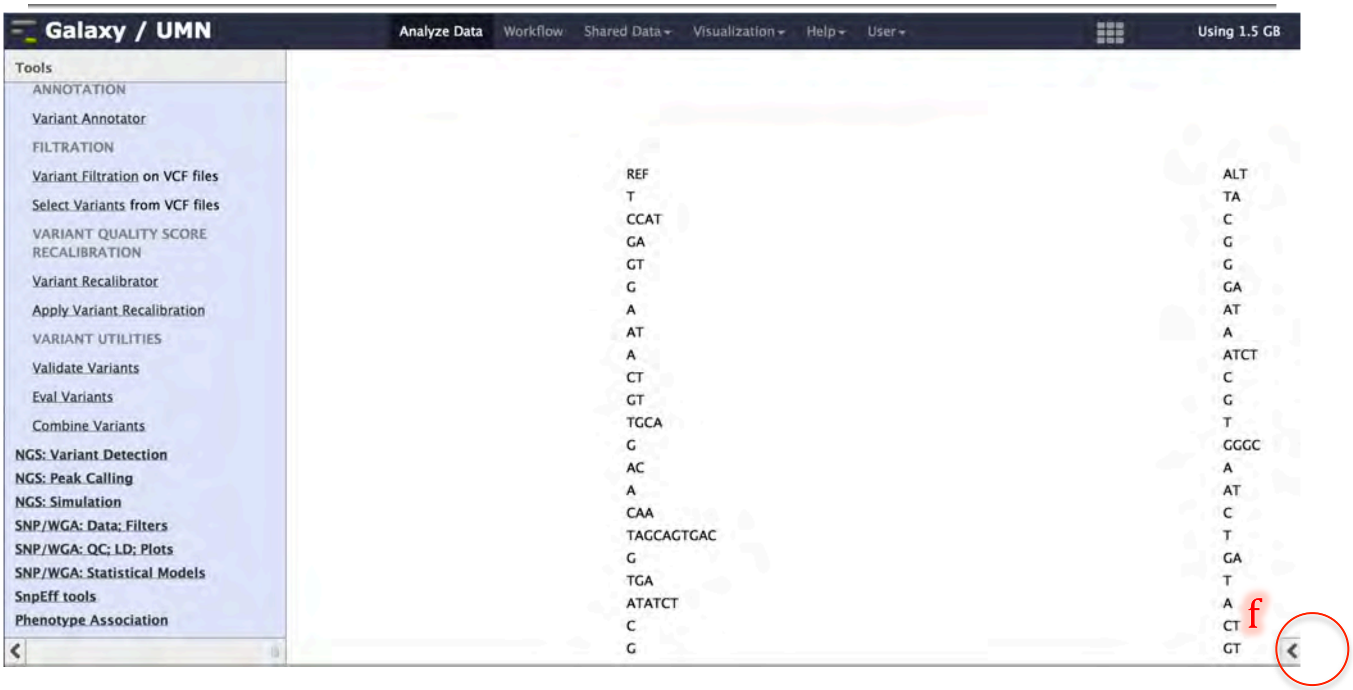

7.10 Combine SNPs and INDELs

a) Load *combine variants* tool from the tool pane: "NGS: GATK Tools -> Combine Variants"
b) Input variant file: **->** "..SNPs ..." (recalibrated SNP vcf)
c) Variant name: -> "snps"
d) Click "Add new Variants to Merge"
e) Select the INDEL file (Input variant file: **->** "..INDELs ..." (recibrated INDEL vcf))
f) Variant name: -> "indels"
g) Using reference genome: -> Homo sapiens hg19_canonical (GATK)
h) Click "Execute"

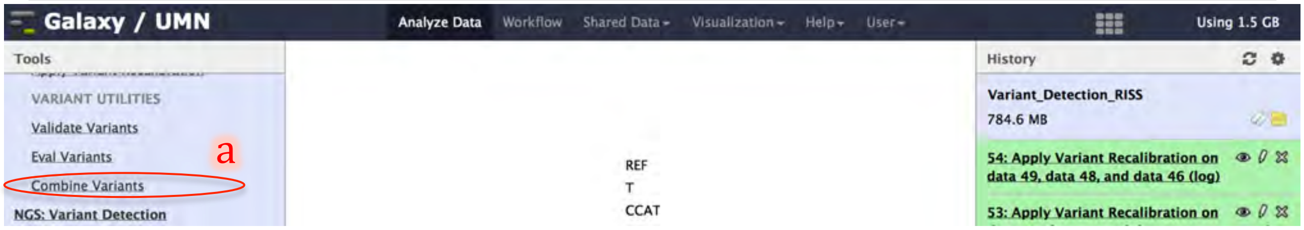

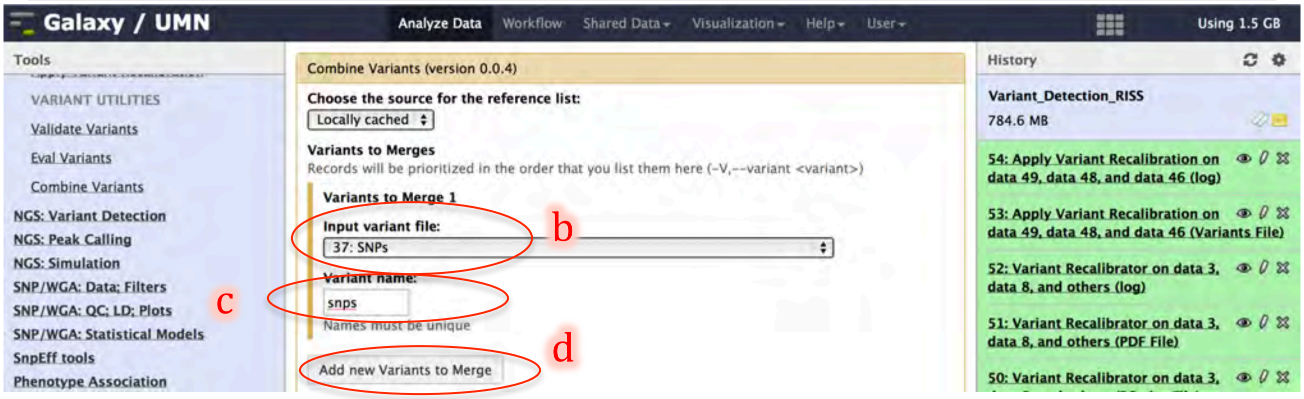

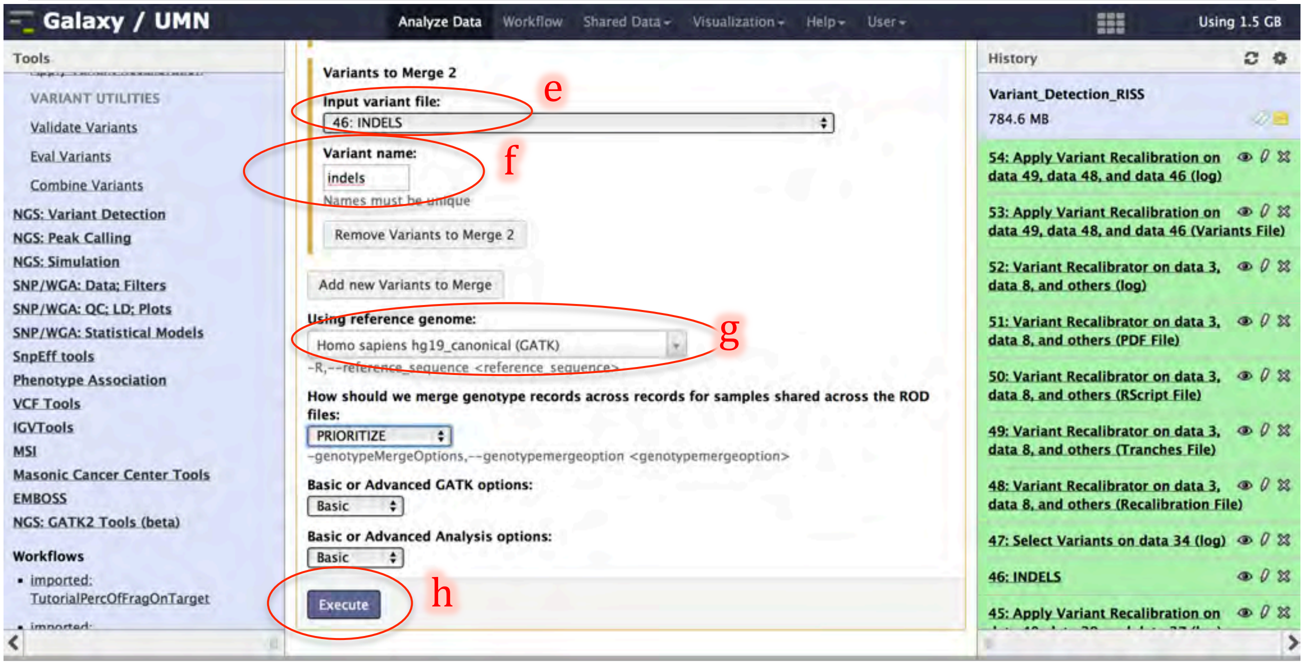

Variant Annotation

7.11 Annotate variants using SnpEff

a) Load *variant annotation* tool from the tool pane: “SnpEff tools -> SnpEff Variant effect and annotation”
b) Sequence changes (SNPs, MNPs, InDels): -> “Combine Variants…. (variants)
c) Genome: -> hg19
d) Upstream / Downstream length: -> 200 bases

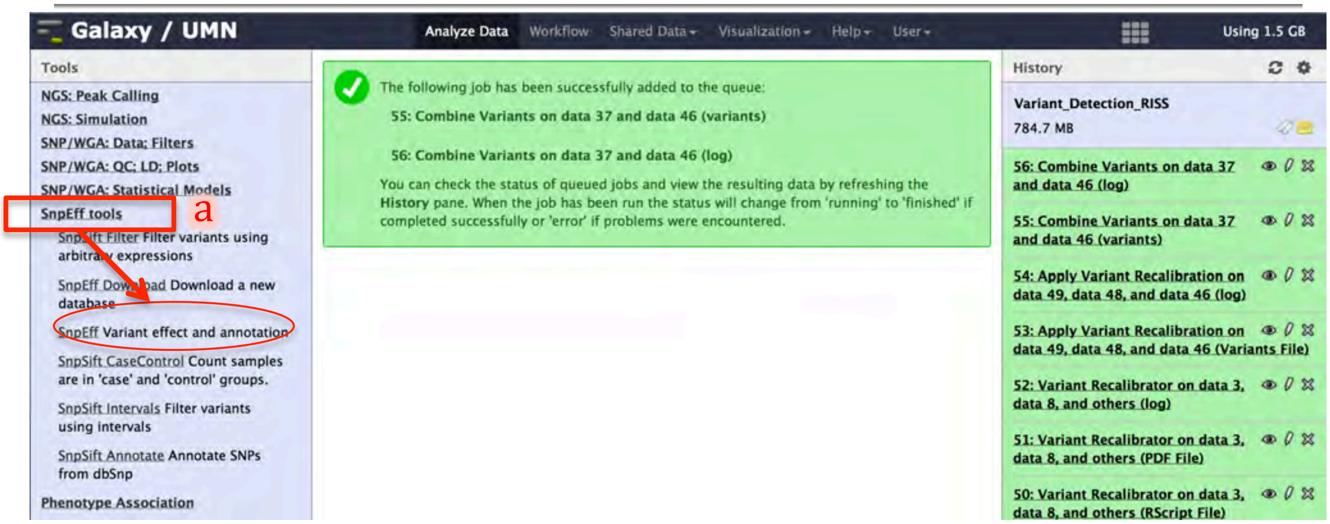

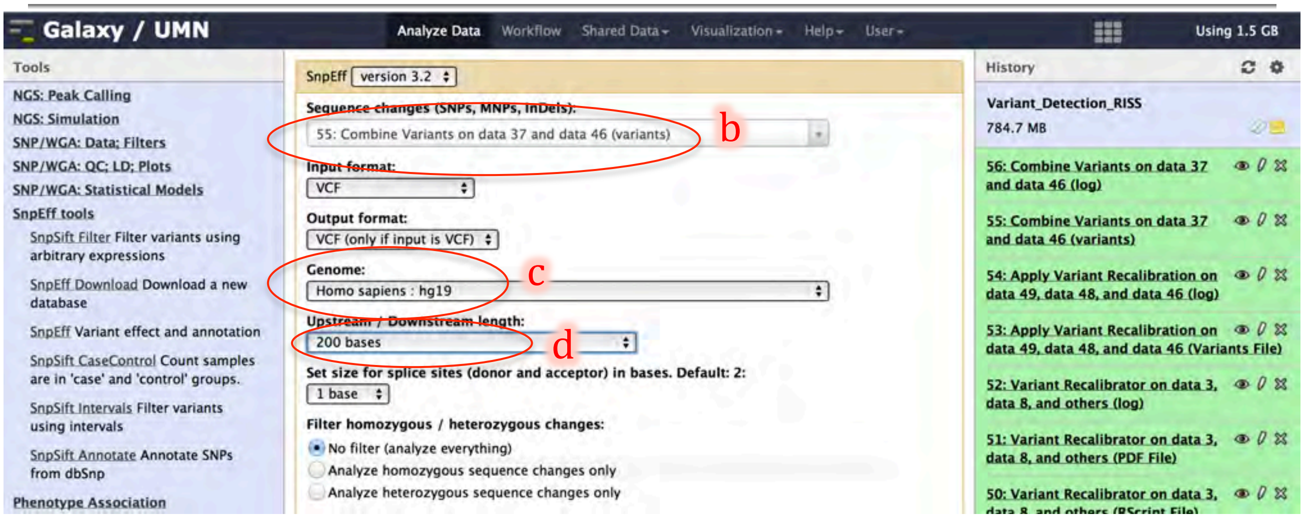

e) Click “Execute”

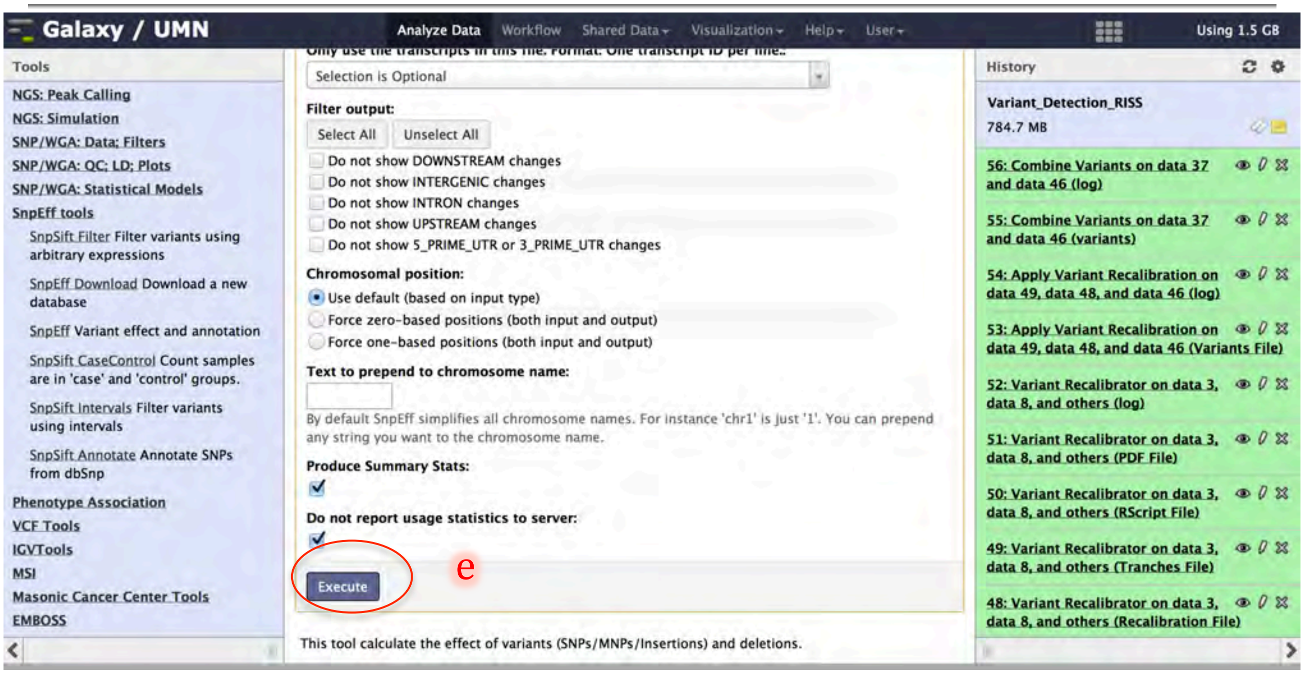

7.12 Review Annotated Variants

a) In the history pane click the eye icon next to the VCF file produced by the "SnpEff tools -> SnpEff Variant effect and annotation" tool
b) Notice the **INFO** is now appended with annotation information such as effect of the variant e.g., NON_SYNONYMOUS CODING

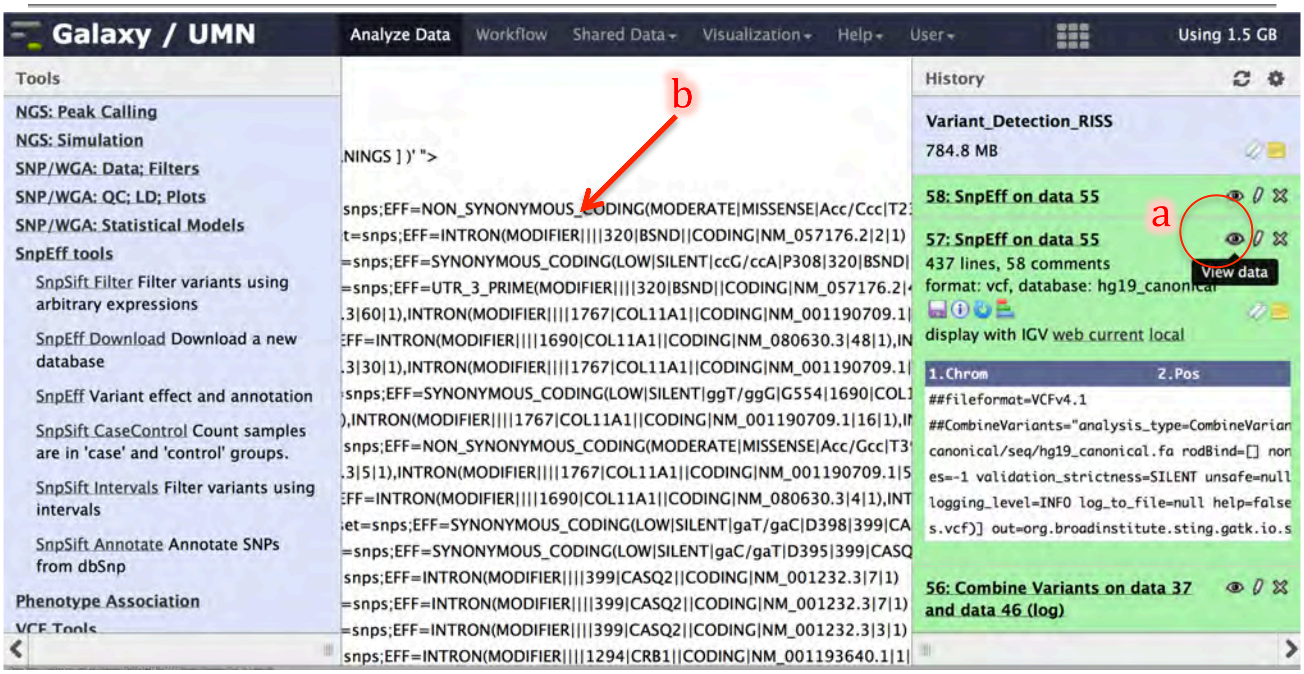

Supplement: Additional file 1 — Galaxy workflow Guide for Variant detection. [file 1756-0500-7-314-S1.pdf]
